# Transcriptome Analysis Reveals Endogenous Hormone Changes during Spike Development in *Phalaenopsis*

**DOI:** 10.3390/ijms231810461

**Published:** 2022-09-09

**Authors:** Zuo Li, Wenfang Xiao, Heming Chen, Genfa Zhu, Fubing Lv

**Affiliations:** Guangdong Key Laboratory of Ornamental Plant Germplasm Innovation and Utilization, Environmental Horticulture Research Institute, Guangdong Academy of Agricultural Sciences, Guangzhou 510640, China

**Keywords:** axillary bud, moth orchid, plant hormone, RNA sequencing, spike initiation

## Abstract

*Phalaenopsis* orchids are popular worldwide due to their high ornamental and economic value; the spike and inflorescence formation of their flowers could be efficiently controlled under proper conditions. In this study, transcriptomic profiles and endogenous hormone changes were investigated to better understand the spike formation of *Phalaenopsis*. Morphological observations revealed four spike initiation statuses (i.e., S0: the status refers to axillary buds remaining dormant in the leaf axils; S1: the status refers to the 0.5 cm-long initial spike; S2: the status refers to the 1 cm-long spike; S3: the status refers to the 3 cm-long spike) during the process of spike development, while anatomical observations revealed four related statuses of inflorescence primordium differentiation. A total of 4080 differentially expressed genes were identified based on pairwise comparisons of the transcriptomic data obtained from the S0 to S3 samples; high levels of differential gene expression were mostly observed in S1 vs. S2, followed by S0 vs. S1. Then, the contents of 12 endogenous hormones (e.g., irindole-3-acetic acid (IAA), salicylic acid (SA), abscisic acid (ABA), gibberellins, and cytokinins) were measured. The results showed that the ABA content was decreased from S0 to S1, while the gibberellic acid 1 (GA1) content exhibited an opposite trend, indicating the reduction in ABA levels combined with the increase in GA1 levels in S0 promoted the axillary bud dormancy breaking, preparing for the following spike initiation. The *GA20 oxidase* and *ABA 8′-hydroxylase* genes, which are involved in endogenous hormone metabolism and signaling pathways, displayed similar expression patterns, suggesting they were probably the key genes participating in the GA and ABA regulation. Taken together, the findings of this study indicate that GA and ABA may be the key endogenous hormones breaking the dormancy and promoting the germination of axillary buds in *Phalaenopsis*.

## 1. Introduction

*Phalaenopsis*, belonging to the Orchidaceae family and well known for its flowers, is the most important potted plant in the world. *Phalaenopsis* orchids demonstrate various floral colors and patterns, and the spike and inflorescence formation of their flowers can be controlled under proper conditions. For example, a high temperature (>28 °C) was used to promote vegetative growth and inhibit spike initiation of young *Phalaenopsis* plants during production [1]. A moderately low temperature can promote *Phalaenopsis* spiking, unlike some other plants, which need colder temperatures to initiate vernalization [2]. After treatment at 25–20 °C/20–15 °C (day/night) for six weeks, the axillary buds of *Phalaenopsis* broke dormancy and protruded from the leaf base to develop into floral spikes [3]. The key to precisely control the flowering date of *Phalaenopsis* is the start time of low-temperature induction, and a number of studies have also indicated that a moderately low temperature at night is necessary for *Phalaenopsis* flowering [1,3].

Besides temperature, other factors such as light and sucrose concentration might also affect *Phalaenopsis* axillary bud maturation and spike initiation. Lowering irradiance can delay the spike initiation of *Phalaenopsis* [4]. Several studies have illustrated a positive relationship between sucrose concentration and spiking of *Phalaenopsis* [5,6,7,8,9]. The shortened stem of *Phalaenopsis* as a pool can store enough sucrose from all source leaves, which can provide the carbon source for spiking when *Phalaenopsis* is under suitable flowering conditions and maintain the viability of dormant axillary bud(s) when *Phalaenopsis* is exposed to a spiking inhibition condition [10].

Moreover, plant endogenous hormones may play an important role in *Phalaenopsis* spiking, but the relative molecular evidence is currently scarce. A moderately low temperature may alter the hormone homeostasis, promoting plants to release abscisic acid (ABA) or accumulate auxin and cytokinin to enhance cell proliferation [11]. In addition, the expression of genes regulating flowering time during the reproductive phase change in the gibberellic acid (GA) pathway is also induced by a low temperature [12]. Moreover, protecting *Phalaenopsis* from stress conditions can prevent axillary bud degeneration, maximize axillary bud growth and maturation, and finally, enhance spiking [11]. Overall, spiking of *Phalaenopsis* is controlled by genetic and environmental cues. However, only a few genes, miRNAs, and proteins related to *Phalaenopsis* flowering have been identified and characterized recently [13,14,15,16,17]. Specifically, from comprehensive analyses of the transcriptome, sRNAs, and degradome, the results identified four microRNAs (miRNAs) as low-temperature-induced miRNAs, two of which may be components of a regulatory pathway mediating the transition from the vegetative to the reproductive phase in *Phalaenopsis*; besides, low-temperature-responsive small RNAs (sRNAs) were validated, which suggested that sRNA-directed gene silencing may also regulate ion homeostasis under low-ambient-temperature treatment in *Phalaenopsis* [13,14]. Moreover, proteomic analysis research studies revealed a regulation network of the early development of flower buds in *Phalaenopsis amabilis* under low temperature induction, in which 42 differentially expressed proteins of *Phalaenopsis* flower buds were identified and associated with early floral induction [17]. To date, fewer studies have been conducted on the induction of spike-initiation-related signals under controlled low-temperature conditions. To fill this gap, it is necessary to investigate the changes in the main endogenous hormones and the molecular mechanisms of spiking in *Phalaenopsis*.

## 2. Results

### 2.1. Morphological and Cytological Characterization during Spike Development in Phalaenopsis

**Figure 1:**
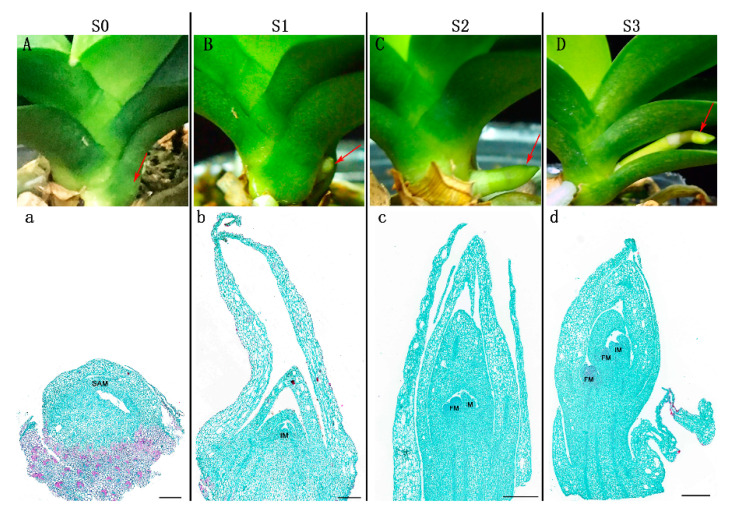
Morphological characterization of the four statuses (S0, S1, S2, and S3) of spike development in *Phalaenopsis*. (**A**) S0: the axillary bud was embedded in leaf axils and remained dormant. (**B**) S1: the spike was 0.5 cm in length. (**C**) S2: the spike was 1 cm in length. (**D**) S3: the spike was 3 cm in length. (**a**–**d**) Paraffin sections of bud/spike tip tissues at the four statuses. Arrows indicate the samples used for paraffin section preparation, transcriptome analyses, and endogenous hormone analysis. SAM: shoot apical meristem, IM: inflorescence primordium, FM: floret primordium. Scale bars in (**a**–**d**) = 500 μm.

Figure 1A illustrates the four statuses (i.e., S0, S1, S2, and S3; see the details in the Materials and Methods Section) of *Phalaenopsis* spike development at a day/night temperature of 26/18 °C, namely from the axillary bud, which is embedded in the leaf axils, after the bud maturation and breaking dormancy under temperature control, the bud developed into the initial spike, then the elongation followed with the spike developing gradually. To reveal the details of how the dormant axillary bud developed into the spike, the bud/spike tissues at the four statuses were prepared for paraffin sections, and the meristematic tissues during spike development were observed (Figure 1). At S0, the shoot apical meristem (SAM) was entirely wrapped and stayed in the dormant bud status. Then, from S1 to S3, with the spike growing and elongated, the meristems became widened and larger gradually. The above observations indicated that the fluctuating day/night temperatures and appropriate low temperatures at night could induce the maturity of axillary buds, break dormancy, and initiate spike development in *Phalaenopsis*.

### 2.2. Quantification of Changes in Endogenous Hormone Contents during Spike Development in Phalaenopsis

**Figure 2:**
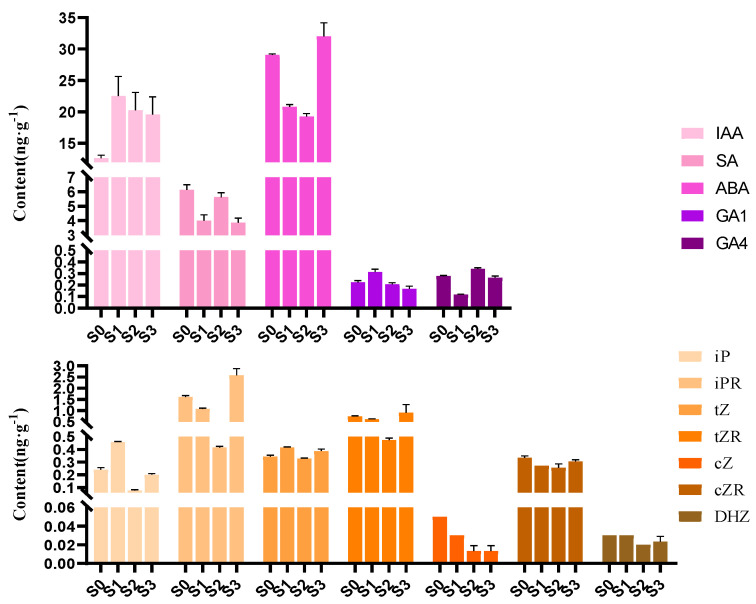
Contents of the 12 endogenous hormones in the four statuses of *Phalaenopsis* spike development. Error bars indicate the standard deviation (SD) of the mean (*n* = 3). IAA (irindole-3-acetic acid), SA (salicylic acid), ABA (abscisic acid), GA1 (gibberellic acid 1), GA4 (gibberellic acid 4), iP (isopentenyl adenine), iPR (isopentenyl adenosine), tZ (*trans*-zeatin), tZR (*trans*-zeatin riboside), cZ (*cis*-zeatin), cZR (*cis*-zeatin riboside), and DHZ (dihydrozeatin).

To investigate the endogenous hormonal changes in the progress of spike formation in *Phalaenopsis*, the contents of 12 endogenous hormones were measured in the four developmental statuses (Figure 2; Supplementary Table S1; Supplementary Figure S1), respectively, including irindole-3-acetic acid (IAA), salicylic acid (SA), ABA, two GAs (GA1 and GA4), and seven cytokinins (CTKs), namely isopentenyl adenine (iP), isopentenyl adenosine (iPR), *trans*-zeatin (tZ), *trans*-zeatin riboside (tZR), *cis*-zeatin (cZ), *cis*-zeatin riboside (cZR), and dihydrozeatin (DHZ). We also tried to measure the contents of GA3, dihydrozeatin riboside (DHZR), and jasmonic acid (JA), which have been examined in previous research, but none of them were detected in the present study.

Significant differences were observed among the contents of the 12 endogenous hormones (Figure 2). Of all the 12 hormones examined, ABA content was the highest at all four statuses, exhibiting a trend of first decreasing (S0 to S2) and then increasing, with the highest value found at S3. GA1 content illustrated the opposite trend to ABA content, which showed a trend of first increasing (S0 to S1) and then decreasing, with the lowest value found at S3. These results indicated that the reduction in ABA content coupled with the increase in GA1 content from S0 to S1 promoted the axillary bud dormancy breaking, preparing for the following spike initiation.

IAA is the most common, naturally occurring, plant hormone of the auxin class. IAA content increased sharply from S0 to S1 and decreased from S1 to S3, representing the same trend as that of GA1 content during the four statuses. It was speculated that IAA stored energy in the apical meristems for bud germination. IAA content decreased when the spike began rapid elongation (from S1 to S3), resulting in apical dominance, which promoted spike growth. In addition, the lowest contents of all seven CTKs were detected at S2.

### 2.3. Transcriptome Analysis

**Figure 3:**
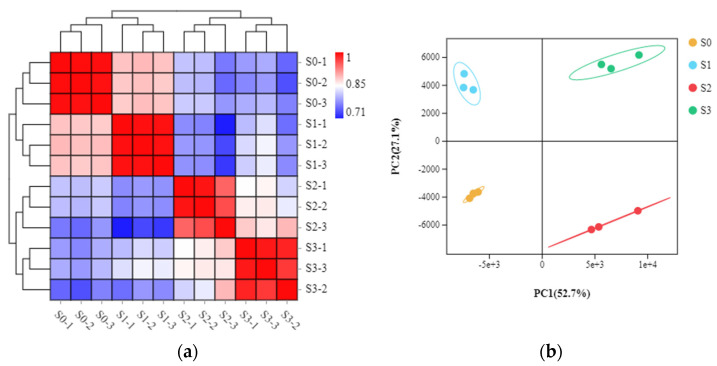
Pairwise Pearson correlation coefficients of the sequencing data from three replicates of each sample collected at the four statuses (**a**). Principal component analysis (PCA) of transcriptome data of the samples collected at the four statuses (**b**).

The differential physiological features in the four statuses prompted us to further investigate the molecular basis of spike formation. A total of 12 libraries were constructed from the same samples above and subjected to RNA sequencing (RNA-seq) analysis, including three biological replicates for each of S0 (denoted as S0-1-3), S1 (denoted as S1-1-3), S2 (denoted as S2-1-3), and S3 (denoted as S3-1-3). The sequencing and assembly information is summarized in Table 1. More than 38 million raw reads were obtained for each library, with the number of raw reads ranging from 38,957,350 to 56,316,414. The Q20 values of all test samples were >98.405%, and the Q30 values were >95.05%. Across the 12 libraries, the average GC content was 50.68%, and the average uniquely mapped reads accounted for 7.23%. All known genes identified by mapping reads to the *Phalaenopsis equestris* genome were found in each of the 12 libraries: 17,623, 17,615, 17,553, 17,690, 17,783, 17,590, 15,751, 15,805, 15,735, 14,988, 15,294, and 14,862 (Table 1). In addition, annotation using the NCBI non-redundant protein (NR) database enabled identification of novel transcripts in each of the 12 libraries (Table 1). Pearson correlation analysis indicated high correlations among the three replicates of each sample (Figure 3a), and principal component analysis (PCA) clustered samples into four groups, corresponding to the four statuses of spike development (Figure 3b). This indicated that the transcriptome data were of high quality.

### 2.4. Differentially Expressed Genes during Spike Development

**Figure 4:**
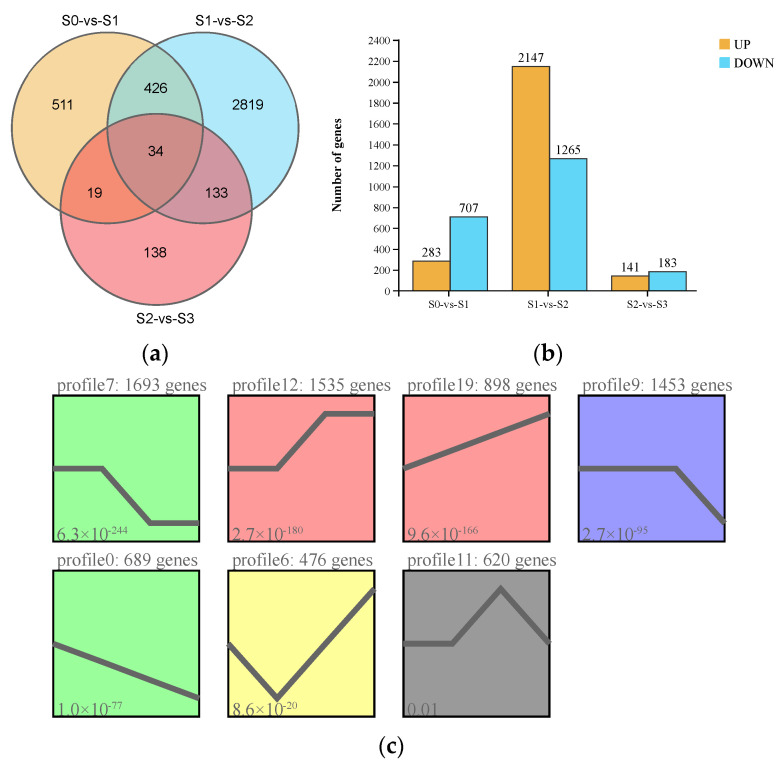
Characterization of differentially expressed genes (DEGs) identified by pairwise comparisons among the four statuses of spike development (S0, S1, S2, and S3; for an explanation, see Figure 1) in *Phalaenopsis*. (**a**) Venn diagram of the DEG numbers in the three pairwise comparisons (S0 vs. S1, S1 vs. S2, and S2 vs. S3). (**b**) Numbers of upregulated and downregulated DEGs in each pairwise comparison. (**c**) Gene expression patterns of the seven profiles identified by Short Time-series Expression Miner (STEM); different colors indicate different expression patterns (*p* ≤ 0.05).

Pairwise comparisons of the transcriptome data of samples collected at the four statuses of spike development were conducted. A total of 4080 DEGs were identified, with the most DEGs (3412) observed in S1 vs. S2 and the fewest (324) seen in S2 vs. S3 (Figure 4a; Supplementary Tables S2–S4). In addition, 34 genes showed significantly differential expression among the four statuses. In S0 vs. S1, 283 and 707 genes were upregulated and downregulated, respectively; in S1 vs. S2, the largest numbers of DEGs were identified, with 2147 and 1265 genes being upregulated and downregulated, respectively, while the fewest numbers of DEGs were seen in S2 vs. S3, with 141 and 183 genes being upregulated and downregulated, respectively (Figure 4b). The number of downregulated DEGs was higher than that of upregulated DEGs in both S0 vs. S1 and S2 vs. S3, while the opposite pattern was observed in S1 vs. S2 (Figure 4b). The 4080 DEGs were clustered into seven significant expression profiles with a *p*-value less than 0.05 by Short Time-series Expression Miner (STEM) analysis (Figure 4c; Supplementary Table S5). Among the seven significant clusters, Profile 7 with the lowest *p*-value included 1693 DEGs, showing rapid downregulation between S1 and S2, but no changes between S0 and S1 and between S2 and S3, respectively. Further, most genes assigned to the “metabolic pathway” were highly represented in Profile 7, suggesting metabolism activities occur during spike development.

### 2.5. GO and KEGG Enrichment Analyses of DEGs

DEGs identified from S0 vs. S1 and S1 vs. S2, the two comparisons with the most DEGs, were annotated against the Gene Ontology (GO) and Kyoto Encyclopedia of Genes and Genomes (KEGG) databases.

**Figure 5:**
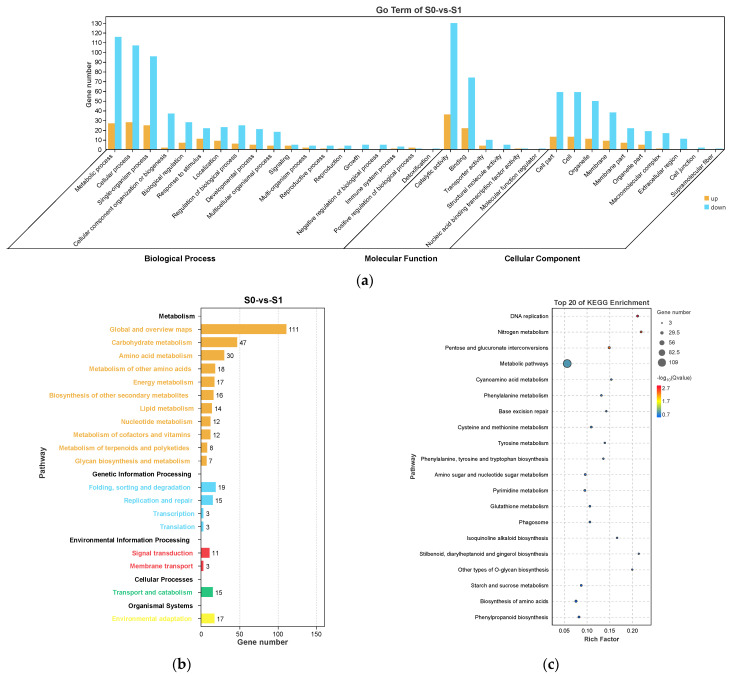
Gene Ontology (GO) enrichment (**a**), KEGG pathway annotation (**b**), and the top 20 KEGG pathways (**c**) of the differentially expressed genes (DEGs) in S0 vs. S1. The area of the bubbles indicates the number of enriched DEGs, while the color of bubbles represents the Q-value indicated in the panel to the right.

In S0 vs. S1, the most enriched GO terms were “metabolic process”, “cellular process”, and “single-organism process” in biological process; “catalytic activity” and “binding” in molecular function; and “cell part”, “cell”, and “organelle” in cellular component (Figure 5a). For the KEGG enrichment analysis, these DEGs were classified into five KEGG categories. “Metabolism” was the most significantly enriched pathway (*p* < 0.05) and included “global and overview maps”, “carbohydrate metabolism”, and “amino acid metabolism” (Figure 5b). The top enriched KEGG pathways included “metabolic pathways”, “amino sugar and nucleotide sugar metabolism”, and “biosynthesis of amino acids”, suggesting these DEGs were involved in the progress of axillary bud induction to spike development (Figure 5c).

**Figure 6:**
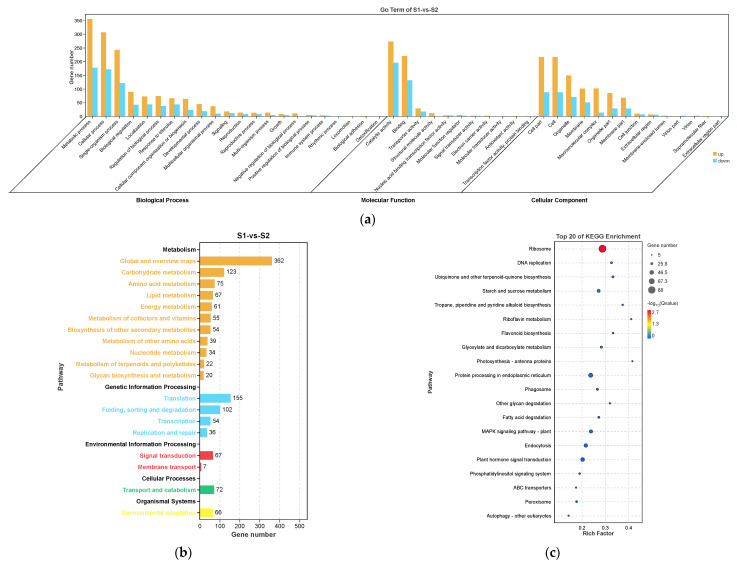
Gene Ontology (GO) enrichment (**a**), KEGG pathway annotation (**b**), and the top 20 KEGG pathways (**c**) of the DEGs in S1 vs. S2. The area of bubbles indicates the number of enriched DEGs, while the color of bubbles indicates the Q-value.

S1 and S2 are the key statuses of spike development. In S1 vs. S2, the most enriched GO terms were similar to those found in S0 vs. S1 (Figure 6a). The most enriched KEGG categories (*p* < 0.05) were “metabolism” and “genetic information processing”, which included “global and overview maps”, “translation”, and “carbohydrate metabolism” (Figure 6b). The top enriched KEGG pathways included “ribosome”, “protein processing in endoplasmic reticulum”, “plant hormone signal transduction”, “endocytosis”, “MAPK signaling system”, and “starch and sucrose metabolism”, suggesting their involvement in the progress of spike elongation (Figure 6c).

### 2.6. DEGs Related to Endogenous Hormone Metabolism and Signaling

**Figure 7:**
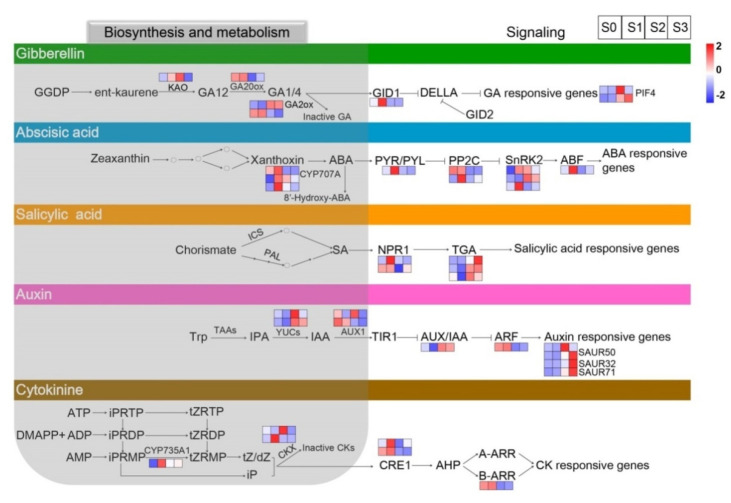
Heatmaps of differentially expressed genes (DEGs) related to plant hormone metabolism and signaling at the four statuses of spike formation of *Phalaenopsis*. From top to bottom, the five panels show DEGs involved in the GAs’, ABA’s, SA’s, auxin’s, and CTKs’ biosynthesis, metabolism, and signal transduction pathways, respectively. Red squares indicate upregulation, whereas blue squares indicate downregulation. The color scale corresponds to the average log_10_ (FPKM+1) and Z-score (normalized by the R software) values. GA, gibberellic acid; ABA, abscisic acid; SA, salicylic acid; CTK, cytokinin; FPKM, fragments per kilobase of transcript per million mapped reads.

Thirty-eight DEGs identified from pairwise comparisons among the four statuses of spike formation were assigned to endogenous-hormone-related biosynthesis, metabolism, and signal transduction pathways (Figure 7; Supplementary Table S6).

In the GA metabolism pathway, *ent*-kaurenoic acid oxidase (KAO), GA20ox, and GA2ox are the main enzymes that synthesize GAs. Among them, *KAO*, as an early GA biosynthesis gene, was upregulated from S0 to S2 and then downregulated at S3. *GA20ox*, coding for a soluble 2-oxoglutarate-dependent dioxygenase (2ODD) in the later stage of GA biosynthesis, was upregulated from S0 to S1 and then downregulated from S1 to S3. GA2ox, as a major GA inactivation enzyme, could control the concentration of bioactive GAs. The GA signaling pathway involved there DEGs, in which the GA receptor gene *GID1* was upregulated from S0 to S1 and then downregulated from S1 to S3. These results indicated that the expression of the genes involved in GA biosynthesis and signal transduction pathways changed with spike development.

In the ABA biosynthesis and metabolism pathways, three DEGs of *ABA8′-hydroxylase* (*CYP707A*) exhibited different fluctuating trends during spike formation, while the main regulated trend was according to one of the *CYP707A* genes with the highest FPKM value, shown in Figure 7, ABA panel, on the top square layer of the three *CYP707A* genes (the gene-id of ncbi_110030934 is detailed in Supplementary Table S6). The *CYP707A* gene showed upregulation at S0 and S1 and then downregulation from S2 to S3. Moreover, seven DEGs involved in ABA signaling were all upregulated at S1 of spike development.

In the IAA biosynthesis and metabolism pathways, two *YUC*s as major genes convert indole-3-pyruvate (IPA) to IAA. The two *YUC*s were downregulated at S0 and S1 and then upregulated from S2 to S3. AUX1 is an important auxin transporter during IAA biosynthesis, and the *AUX1* gene was upregulated from S0 to S1 and then downregulated from S2 to S3. In the IAA signaling pathway, six DEGs were related to auxin-responsive or auxin-transport, including *auxin*/*IAA* (*Aux/IAA*), *auxin response factors* (*ARFs*), and four *small auxin-up RNA*s (*SAUR*s). Among them, the four *SAUR*s showed downregulation during S0 and S1 and upregulation during S2 and S3.

Besides GA, ABA, and auxin, DEGs related to CTKs and SA were also identified. CTKs regulate cell differentiation and play key roles in mediating cell proliferation. In the CTK biosynthesis and metabolism pathways, *cytochrome P450 monooxygenase* (*CYP735A*) was downregulated at S0 and then sharply upregulated at S1. Two crucial enzymes, namely cytokinin dehydrogenase 5 and cytokinin dehydrogenase 3-like, involved in the synthesis of CTKs were both downregulated at S0. Among the four DEGs related to the CTK signaling pathway, *Arabidopsis type B cytokinin response regulator* (*B-ARR*), one of the key genes encoding *aminoglycoside phosphotransferase* (*APH*), was upregulated during S0 and S1 and then downregulated during S2 and S3. Moreover, five DEGs were assigned to the SA signaling pathway, while no DEG was associated with the SA biosynthesis and metabolism pathways.

### 2.7. qRT-PCR Verification

**Figure 8:**
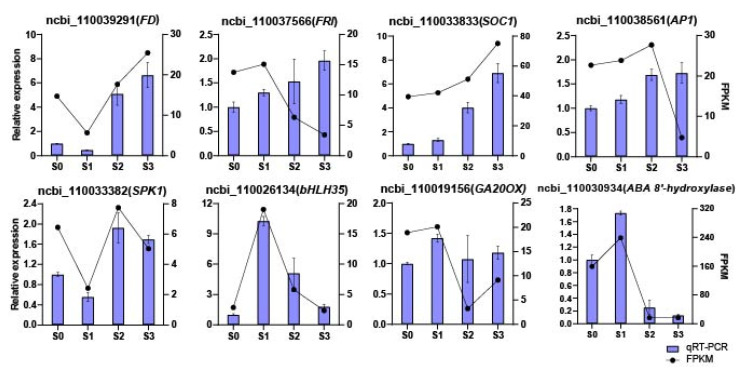
qRT-PCR validation of the expression patterns of 8 differentially expressed genes (DEGs) during spike development. The *x*-axis represents the four statuses; the left *y*-axis represents the relative expression levels normalized from the qRT-PCR results; the right *y*-axis represents the FPKM values from RNA-seq. Error bars are the standard deviation (SD) of the mean (*n* = 3).

Eight DEGs obtained based on the RNA-seq data from the four statuses of spike development were randomly selected for quantitative real-time polymerase chain reaction (qRT-PCR) validation. Among these eight genes, four (i.e., *FD-like*, *FRI*, *SOC1*, and *AP1*) are involved in flower development, two are transcription factors (TFs) involved in spike initiation (*SPK1* and *bHLH35*), and two are key enzymes involved in plant hormone metabolism and signaling (*GA20ox* and *ABA 8′-hydroxylase*) (Figure 8; Supplementary Table S7). The results obtained from qRT-PCR were almost consistent with those from RNA-seq. For example, from S0 to S3, the expression levels of *FD-like*, *FRI*, *SOC1*, and *AP1* were all increased; specifically, *FD-like* and *SOC1* showed higher expression fold changes from S0 to S3, which implied they might play more important roles during spike formation of *Phalaenopsis*. Comparing the two SPIKE-related TFs, namely *SPK1* and *bHLH35*, from S0 to S1, the relative expression level of *SPK1* decreased by 50%, while the relative expression level of *bHLH35* increased dramatically by about 10-fold. Furthermore, the relative expression level of *SPK1* increased from S1 to S2 and then decreased from S2 to S3, while the expression of *bHLH35* continued to decrease from S1 to S3. Moreover, *GA20ox* and *ABA 8′-hydroxylase* displayed similar trends, in which the expression increased from S0 to S1 and then decreased sharply from S1 to S2. Supplementary Table S8 lists the results of qRT-PCR and RNA-seq (based on FPKM values). As shown in Figure 8, the expression patterns of the eight DEGs obtained from qRT-PCR were basically consistent with those from RNA-seq, indicating that the RNA-seq results were reliable.

## 3. Discussion

Axillary buds mainly differentiate and develop from stem apical meristems, and their growth is affected by the interactions among environmental factors, genetic background, and endogenous metabolites. Bud dormancy is a dynamic equilibrium process, and it may break dormancy and germinate if the plant accumulates enough cold energy [18]. During plant growth and development, many physiological activities are controlled by endogenous hormones [19,20]. Endogenous hormones, such as IAA, CTKs, GAs, ABA, SA, and JA, are believed to play a major role in plant flowering progress [21]. Among them, GAs play an important role in the process of floral transition, and the loss of any component activity in GA biosynthesis and signaling may lead to flowering defects [22,23]. In this study, GA1 content was low at the dormant stage of axillary buds (S0) and then increased to the maximum level with germination (S1). Therefore, it was speculated that apical meristem differentiation requires higher GA levels. It would be effective to induce *Phalaenopsis* flowering by applying exogenous GA under low-temperature conditions [24].

Previous studies have demonstrated that different endogenous hormones play different regulatory roles throughout the plant growth and development processes. The dormancy period of plants mainly depends on the balance between the synthesis and metabolism of two key hormones, namely ABA and GA. It was found that dormancy breaking of buds is usually accompanied by a significant decrease in ABA content and an increase in GA content in crops and ornamental plants [25,26]. Studies have shown that ABA is an important signal for inducing dormancy, and in many plants, ABA is involved in the maintenance of plant dormancy [27]. ABA is also an essential endogenous hormone for the establishment and maintenance of seed and shoot dormancy [28]. An increased ABA level in plants may promote dormancy and delay the germination of flower buds [29]. Conversely, it might advance the germination of flower buds. Meanwhile, GA plays a vital role in low-temperature-induced dormancy breaking. For example, long-term low-temperature accumulation could induce the synthesis of GA, which could then promote the degradation of callose and dormancy breaking in poplar trees [18]. *Arabidopsis thaliana cyp707a2* mutant plants inhibited ABA catabolism and affected ABA biosynthesis, resulting in increased ABA accumulation and enhanced dormancy degree [30]. Therefore, it was inferred that GA has an antagonistic effect with ABA and is one of the key endogenous hormones to break dormancy and promote bud germination.

However, the dynamics of endogenous hormones in the processes of the dormancy breaking and germination of axillary buds, as well as spike formation have not been investigated. In our study, the results showed that during the germination and spike formation processes of axillary buds, the expression levels of genes involved in GA biosynthesis were significantly increased, which is consistent with the findings of Yamauchi et al. [31]. In the ABA metabolism pathway, *CYP707A* encodes 8′-hydroxylase, which digests ABA and plays an important role in reducing ABA content in plants. Studies have shown that ABA positively regulates the expression of *CYP707A* family genes at the transcriptional level and initiates its own oxidative inactivation [32,33].

Besides, GA and brassinolide could also positively regulate the expression of the *CYP707A* family of genes at the transcriptional level, indirectly affecting the catabolism of endogenous ABA in plants [33]. In addition, studies have shown that lower auxin content in leaf axil cells is conducive to the initiation of axillary meristems, while the accumulation of auxin in leaf axils inhibits the initiation of meristems [34]. The *Phalaenopsis* buds are derived from the dormant meristem of leaf axils, and the IAA level is low in the early stage of bud differentiation (bud length: 0–2 cm), which is beneficial to the initiation of dormancy breaking and the development of early inflorescence primordium, consistent with the conclusion of a study on *Arabidopsis* [35].

While investigating the molecular mechanism and regulation of floral transition in *Phalaenopsis*, Jiang et al. [36] found an SVP protein binding to the promoter of *FLOWERING LOCUS T* (*FT*) gene to inhibit its expression, thereby affecting the floral transition of *Phalaenopsis*. bHLH TF SPK1 has been identified to play a key role in the early axillary bud germination and spiking of *Phalaenopsis* [11]. Probably, the heterodimer of SPK1/bHLH35 may inhibit the transactivation of the downstream spiking gene(s). It has been reported that FLOWERING LOCUS D (FD) (a flowering activator) can interact with TFL1 (a flowering repressor) to regulate flowering rhythm [37]. FT is a common flowering marker in many plant species. In the SAM, FT interacts with FD to promote flowering and activate genes involved in floral meristem development; therefore, FT may represent a long-distance signal during the flowering process [38]. *Phalaenopsis* FT interacting with FD might regulate downstream genes such as *AP1*, *SOC1*, and *LEAFY* [39]. Moreover, FT and FD are interdependent partners through protein interaction and act at the shoot apex to promote floral transition and to initiate floral development through transcriptional activation of a floral meristem identity gene, *APETALA1* (*AP1*). FT protein is transported to the SAM via the phloem [40]. In this study, the genes related to flower development (*FD-like*, *FRI*, *SOC1*, and *AP1*) and spike initiation (*SPK1* and *bHLH35*) were differentially expressed during spike development. These genes might be the key regulatory genes involved in spike formation, providing a theoretical basis and guidance for subsequent studies.

## 4. Materials and Methods

### 4.1. Plant Materials and Growth Conditions

*Phalaenopsis* Sogo Vivien ‘Sogo F858′ plants at the five-leaf stage were collected from the orchid nursery of the Environmental Horticultural Research Institute of Guangdong Academy of Agricultural Sciences, China (23°23′51″ N, 113°26′28″ E). For spike induction, *Phalaenopsis* plants were acclimatized in a growth chamber with a photoperiod of 12/12 h, a day/night temperature of 26/18 °C, and a relative humidity of 70%. After 8 weeks at the same controlled conditions, the *Phalaenopsis* plants exhibited different spiking statuses. About half of the plants were spiking, but with different spike lengths (0.5–3 cm), and the other half remained non-spiking. Four statuses of spike development were defined: S0, the axillary bud remained dormant in leaf axils; S1, the spike reached 0.5 cm in length; S2, the spike reached 1 cm in length; S3, the spike reached 3 cm in length. Three biological replicates were conducted for each status. All the prepared samples were placed immediately in liquid nitrogen and stored at −80 °C for RNA isolation and endogenous hormone determination.

### 4.2. Tissue Paraffin Section Preparation

*Phalaenopsis* tissue samples of dormant axillary buds (S0) and spikes (S1–S3) were collected. The microstructure changes from the dormant axillary bud to spike were characterized through the observation of paraffin sections. Paraffin sections were prepared using the following protocols. Tissue samples were fixed in formalin acetic acid alcohol solution (FAA, 70% ethyl alcohol: glacial acetic acid: 37% formaldehyde, 18:1:1) for one week. Fixed samples were dehydrated with serial solutions of ethanol (75% for 4 h, 85% for 2 h, 90% for 2 h, 95% for 1 h, and 100% for 30 min twice, *v*/*v*). Next, the samples were immersed in an ethanol and xylene mixture for 10 min and then in a xylene solution for 10 min twice. After the dehydration was complete, the samples were immersed in the melted paraffin and placed three times in a 65 °C incubator, then the samples were embedded in pure paraffin and cooled in a −20 °C frozen platform (Junjie Electronics JB-L5, Wuhan, China) for producing wax blocks. The wax blocks were sliced vertically into sections with a thickness of 4 μm with a microtome (Leica Instrument RM2016, Shanghai, China). The sections were stained with toluidine blue dye (Servicebio G1032, Wuhan, China) and were observed and photographed under an optical transmission microscope (Nikon Eclipse E100, Nikon DS-U3, Nikon Instruments (Shanghai) Co., Ltd., Shanghai, China) in bright field mode.

### 4.3. Determination of the Contents of Endogenous Hormones

The buds/spikes at S0, S1, S2, and S3 (approximately 0.5 g fresh weight per sample) were collected, frozen in liquid nitrogen, and stored at −80 °C. Three biological replicates were performed for each of the four samples. The contents of endogenous hormones were determined by Wuhan Greensword Creation Technology Co., Ltd. (Wuhan, China) according to a previously reported method with slight modifications [41]. Plant hormone analysis was performed on a Thermo Scientific Ultimate 3000 UHPLC System equipped with a TSQ Quantiva-Stage Quadrupole Mass Spectrometer (Thermo Fisher Scientific, Sunnyvale, CA, USA). The Xcalibur v2.1 software (Thermo Fisher Scientific, Sunnyvale, CA, USA) was used for data processing.

### 4.4. RNA-Seq Library Preparation and Sequencing

The buds/spikes at S0, S1, S2, and S3 (approximately 0.5 g fresh weight per sample) were collected, frozen in liquid nitrogen, and stored at −80 °C. Three biological replicates were performed for each of the four samples. Total RNA was extracted using a Trizol reagent kit (Invitrogen, Walthman, MA, USA) according to the manufacturer’s protocol. RNA quality and content were assessed on an Agilent 2100 Bioanalyzer (Agilent Technologies, Santa Clara, CA, USA) and RNase-free agarose gel electrophoresis. The mRNA was enriched by oligo (dT) beads, fragmented, and reverse-transcribed to the first-strand cDNA with random primers. Then, the second-strand cDNA synthesis was carried out using polymerase I. The cDNA fragments were purified using the QiaQuick PCR extraction kit (Qiagen, Venlo, The Netherlands), end-repaired, poly(A)-tailed, and ligated with Illumina sequencing adapters. The ligation products were size-selected using AMPure XP beads and PCR amplification. Then, a total of 12 cDNA libraries were constructed and sequenced using an Illumina HiSeq 2500 platform by Genedenovo Biotechnology, Co., Ltd. (Guangzhou, China).

### 4.5. Analysis of Transcriptomic Data

High-quality clean reads were obtained by removing adapters, reads containing >10% of ambiguous nucleotides (N), and reads containing >50% of low-quality (Q-value ≤ 20) bases. The subsequent analyses were based on high-quality, clean data. The paired-end reads were mapped to the reference genome of *Phalaenopsis equestris* [42] using HISAT2 2.0.4 (Johns Hopkins University, Maryland, USA) [43]. The fragments per kilobase of transcript per million mapped reads (FPKM) value was calculated to represent the gene expression level. DESeq2 [44] was used to identify DEGs between (and by edgeR [45] between two samples), with FDR < 0.05 and absolute log2 (fold-change) ≥ 2 as the threshold. The obtained DEGs were subjected to GO and KEGG enrichment analyses.

### 4.6. Verification of Transcriptome Data Using qRT-PCR

qRT-PCR analysis was used to verify the results obtained from RNA-seq. Total RNA was reverse-transcribed using HiScript II Q RT SuperMix Kit with gDNA wiper (Vazyme, Nanjing, China) according to the manufacturer’s instructions. qRT-PCR was performed using PowerUp SYBR Green Master Mix Kit on a QuantStudio 3 system (Applied Biosystems, Carlsbad, CA, USA). PCR reaction procedures were 50 °C for 2 min, 95 °C for 10 min, followed by 40 cycles of 95 °C for 15 s, and 60 °C for 1 min and then a melt curve stage. PCR reactions were performed in triplicate, using *PACT4* as the reference gene. The relative expression level of target genes was analyzed by the 2^−ΔΔCt^ method [46]. The primers used for qRT-PCR analysis are listed in Supplementary Table S7.

### 4.7. Data Analysis

qRT-PCR results and the contents of endogenous hormones were plotted using GraphPad Prism and Excel software. The data are presented as the mean ± standard deviation (SD).

## 5. Conclusions

*Phalaenopsis* orchids are popular around the world as high-commercial-value ornamental plants; however, their floral organ development and flower-regulating mechanisms remain largely unknown due to the long vegetative growth period and various variations in flowering among species in the genus. In this study, transcriptomic profiles and endogenous hormone changes were investigated to better understand the process of spike development in *Phalaenopsis*. The findings indicated that GA and ABA may be the key endogenous hormones breaking the dormancy and promoting the initiation of the axillary buds of *Phalaenopsis* orchids and would be more effective in regulating spiking than a low ambient temperature only.

## Figures and Tables

**Table 1:** Summary of the transcriptome sequencing data of the 12 libraries constructed using corresponding samples at the four statuses.

Sample	Raw Reads	Clean Reads	Clean Bases	Q20 (%)	Q30 (%)	GC Content (%)	Total Mapped Reads (%)	Uniquely Mapped Reads (%)	Multiple Mapped Reads (%)	Known Genes (%)	Novel Transcripts (%)
S0-1	46,268,476	46,204,170	6.84 G	98.52	95.41	50.43	4,775,886 (10.35%)	4,617,424 (10.01%)	158,462 (0.34%)	17,623 (82.92%)	786 (71.58%)
S0-2	45,653,422	45,595,708	6.75 G	98.54	95.46	50.32	4,713,723 (10.35%)	4,554,978 (10.00%)	158,745 (0.35%)	17,615 (82.89%)	817 (74.41%)
S0-3	42,591,868	42,540,762	6.29 G	98.60	95.61	50.74	4,409,513 (10.39%)	4,263,167 (10.04%)	146,346 (0.34%)	17,553 (82.59%)	781 (71.13%)
S1-1	47,366,732	47,310,098	7.01 G	98.41	95.09	50.18	6,711,949 (14.20%)	6,485,835 (13.72%)	226,114 (0.48%)	17,690 (83.24%)	855 (77.87%)
S1-2	56,316,414	56,249,948	8.36 G	98.47	95.22	50.17	8,420,230 (15.00%)	8,152,425 (14.52%)	267,805 (0.48%)	17,783 (83.68%)	856 (77.96%)
S1-3	38,957,350	38,913,044	5.72 G	98.40	95.07	51.01	5,514,499 (14.19%)	5,326,216 (13.71%)	188,283 (0.48%)	17,590 (82.77%)	841 (76.59%)
S2-1	46,758,952	46,713,386	6.96 G	98.53	95.40	50.27	1,266,904 (2.71%)	1,226,651 (2.63%)	40,253 (0.09%)	15,751 (74.12%)	574 (52.28%)
S2-2	49,996,596	49,951,736	7.44 G	98.63	95.66	50.40	1,343,225 (2.69%)	1,302,030 (2.61%)	41,195 (0.08%)	15,805 (74.37%)	568 (51.73%)
S2-3	47,591,140	47,541,360	7.08 G	98.49	95.30	50.43	1,495,941 (3.15%)	1,456,948 (3.07%)	38,993 (0.08%)	15,735 (74.04%)	570 (51.91%)
S3-1	49,067,312	49,002,090	7.29 G	98.60	95.59	51.37	1,074,665 (2.20%)	1,045,590 (2.14%)	29,075 (0.06%)	14,988 (70.53%)	534 (48.63%)
S3-2	51,300,046	51,170,158	7.53 G	98.51	95.36	51.37	1,210,066 (2.37%)	1,174,323 (2.30%)	35,743 (0.07%)	15,294 (71.96%)	586 (53.37%)
S3-3	47,534,374	47,481,972	7.07 G	98.40	95.05	51.44	987,029 (2.08%)	959,350 (2.02%)	27,679 (0.06%)	14,862 (69.93%)	523 (47.63%)

## Data Availability

Not applicable.

## Supplementary Materials

The Supporting Information can be downloaded at: https://www.mdpi.com/article/10.3390/ijms231810461/s1.
