# Supplementary material for: Transcriptome Analysis Reveals Endogenous Hormone Changes during Spike Development in Phalaenopsis
Source: Int J Mol Sci. 2022 Sep 9;23(18):10461. doi: 10.3390/ijms231810461 (PMC9499595; doi:10.3390/ijms231810461)
Supplement: Supplementary file 1 [file ijms-23-10461-s001.zip › Table S7.pdf]

Table S7 Primers for qRT-PCR analysis

Gene ID	Symbol	Forward primer (5'→3')	Reverse primer (5'→3')	Product（bp）
ncbi_110039291	*FD*	GCCCGAAAACAGGCATACAC	TAAAATGGAGCGGTTGAGGT	161
ncbi_110037566	*FRI*	GGAAAATGGTGTTCAGGCG	CCAAACGCAGCAAGCACA	198
ncbi_110033833	*SOC1*	GCTGGAAGAGCAGGTAGTG	ACAGAGATAAGGTTGTGTGAGG	101
ncbi_110038561	*AP1*	GATGAGGAGGAACGGGCAAG	ACAGCCGAAGCAGACACAA	93
ncbi_110033382	*SPK1*	AAGAAGACAAAGCACCAGCA	GGGCGTAAGAAGCAGGCA	192
ncbi_110026134	*bHLH35*	CAGCAGGAAGAGACACGCA	CAAAGGCTCACGCCACTCAT	222
ncbi_110025156	*GA20OX*	GAGGTGTCTGGGTGATGGTT	CCTTCTGCTGCTTGGCTTCC	370
ncbi_110030934	*CYP707A*	TAATACCGAAGGGGTGGAAGG	GAGGTGGTGGAGAAAGACGAG	206
	PACT4	AAACTGCCAAGACGACCTCA	CCCTGCTGCTTCCATACCAA	137
