# Supplementary figures and images for: Transcriptome Analysis Reveals Endogenous Hormone Changes during Spike Development in Phalaenopsis
Source: Int J Mol Sci. 2022 Sep 9;23(18):10461. doi: 10.3390/ijms231810461 (PMC9499595; doi:10.3390/ijms231810461)

Figure S1 Peak plot of HPLC

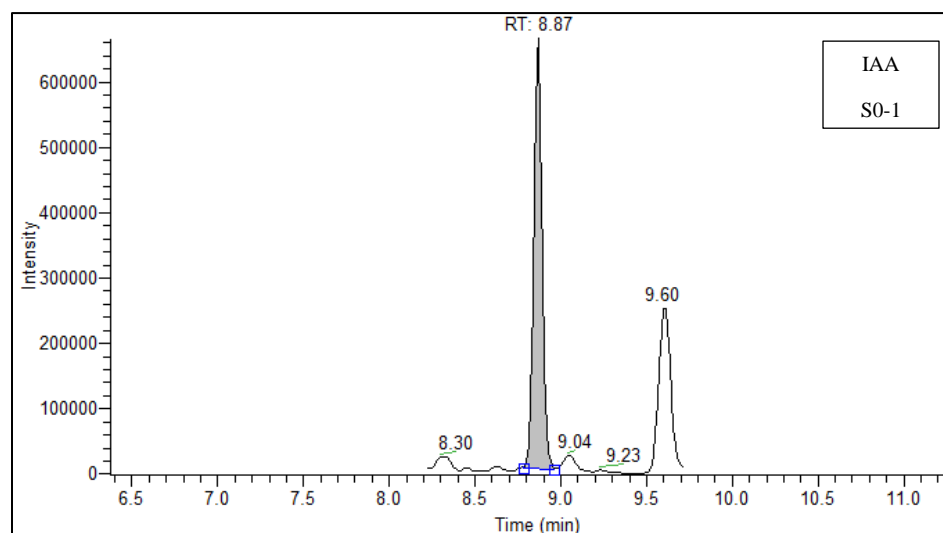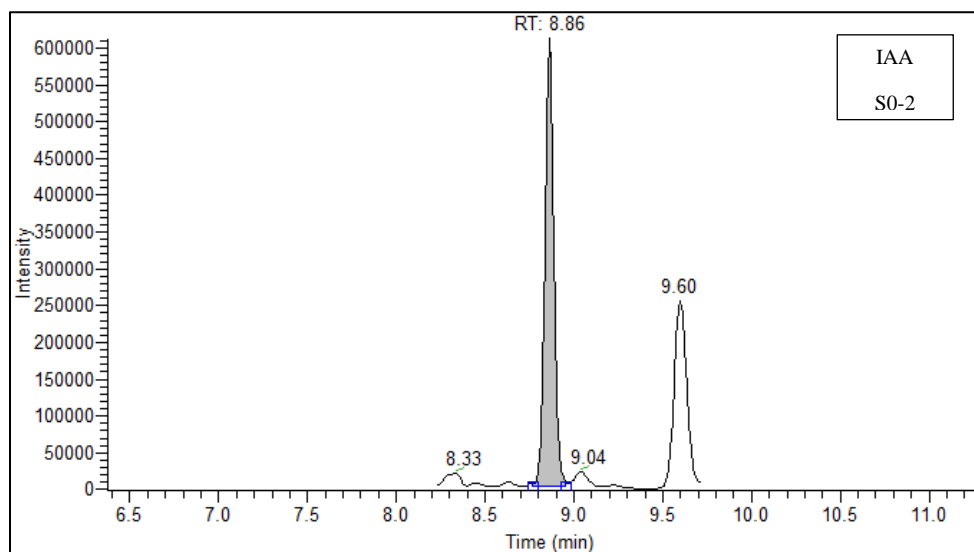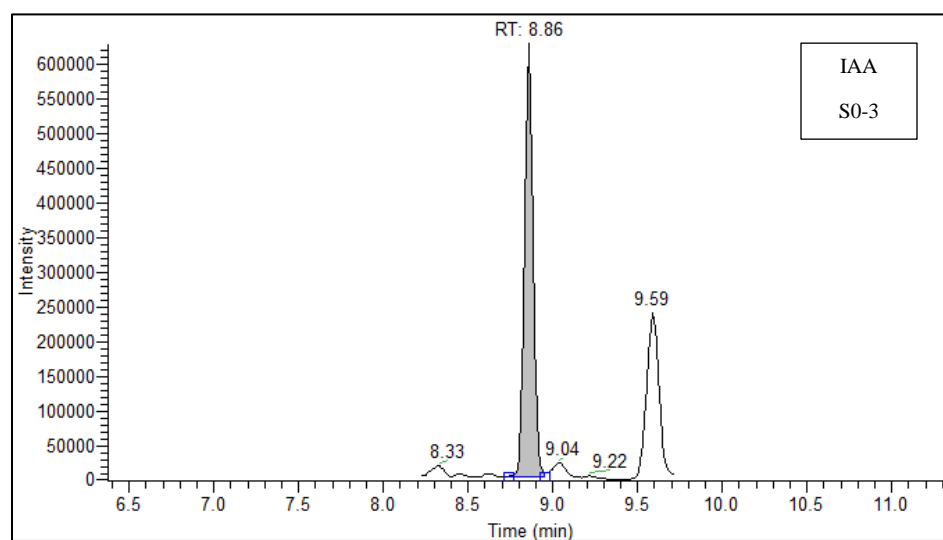

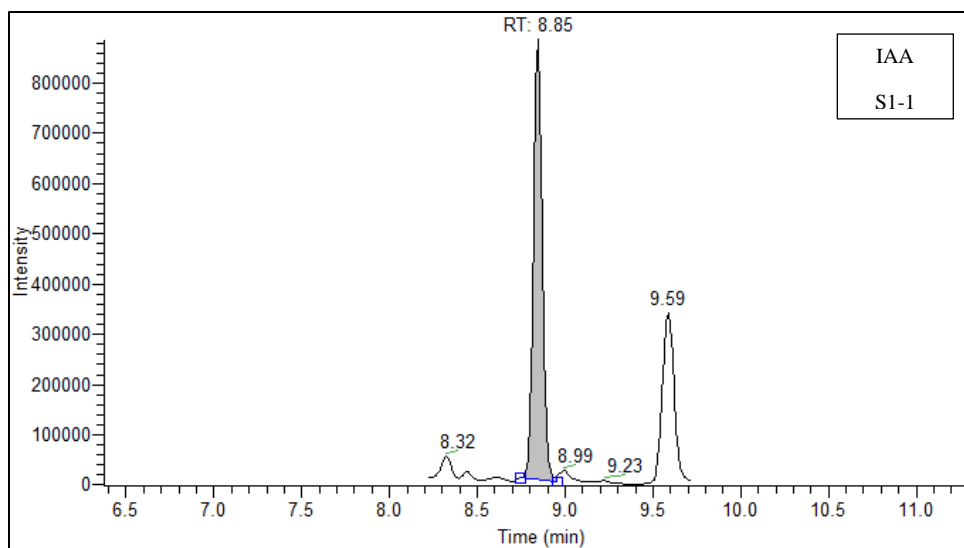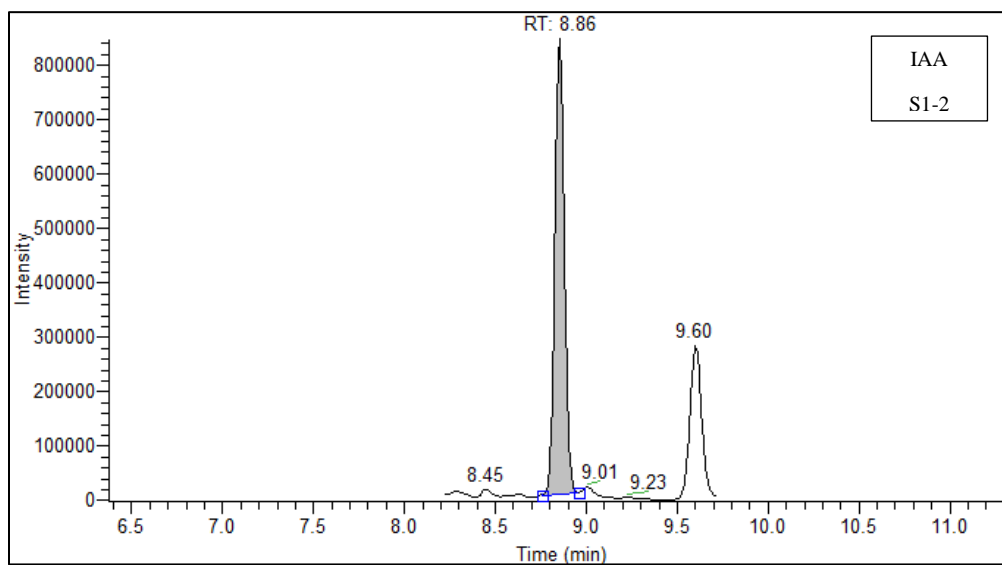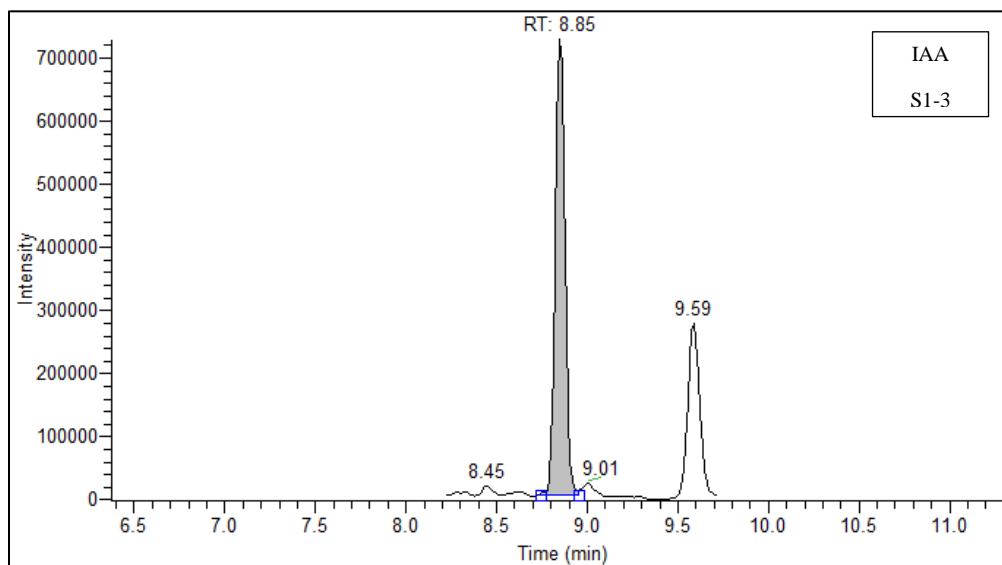

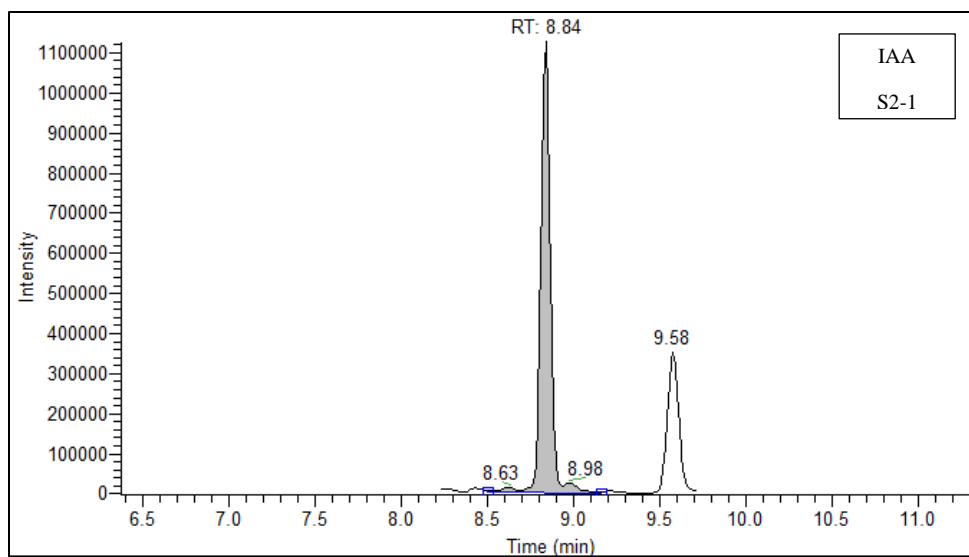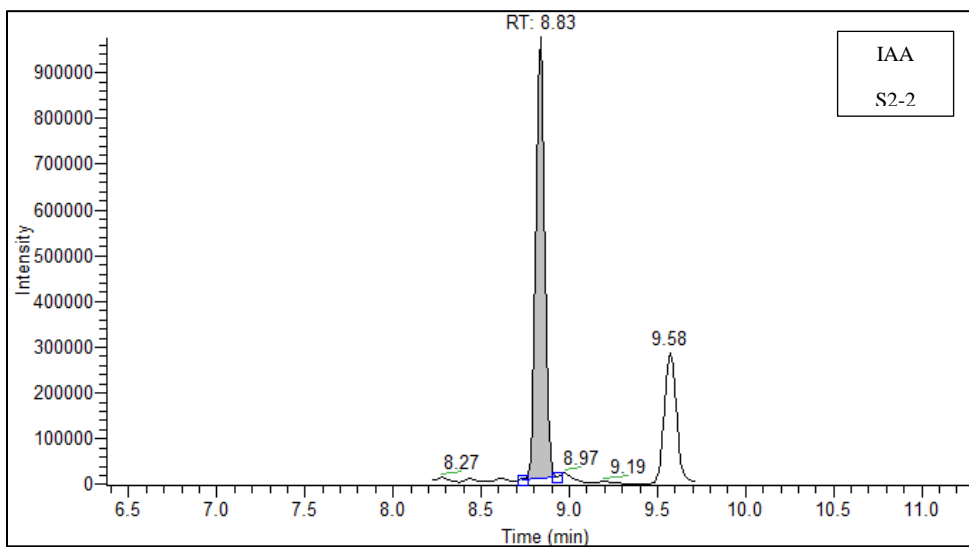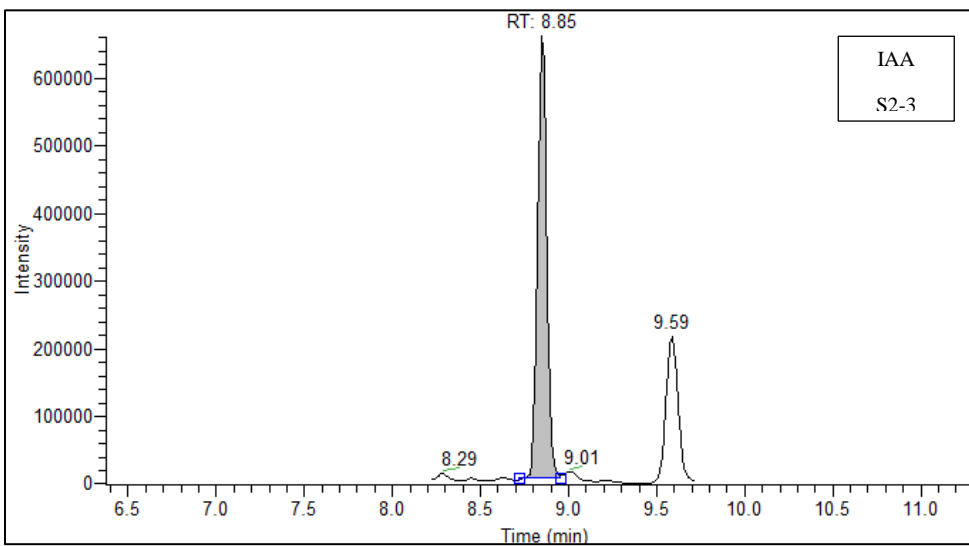

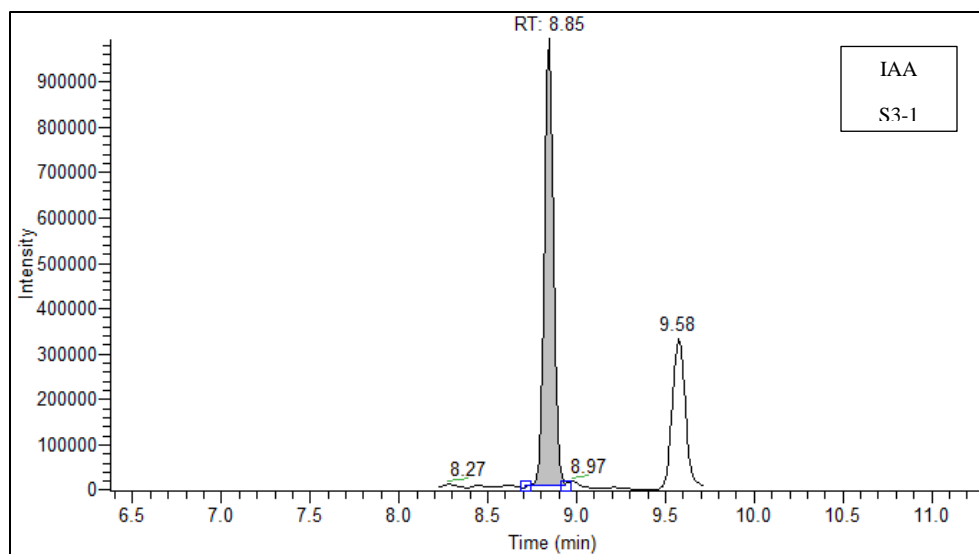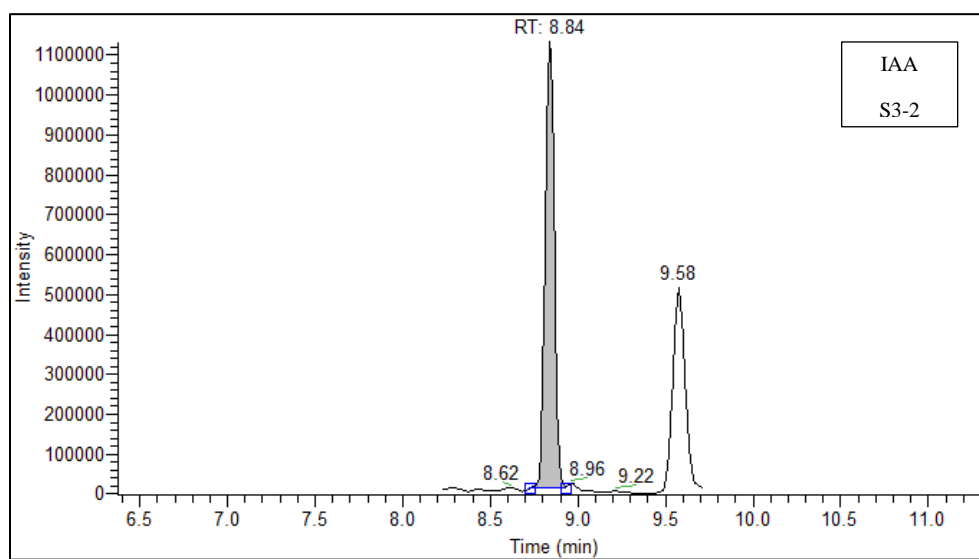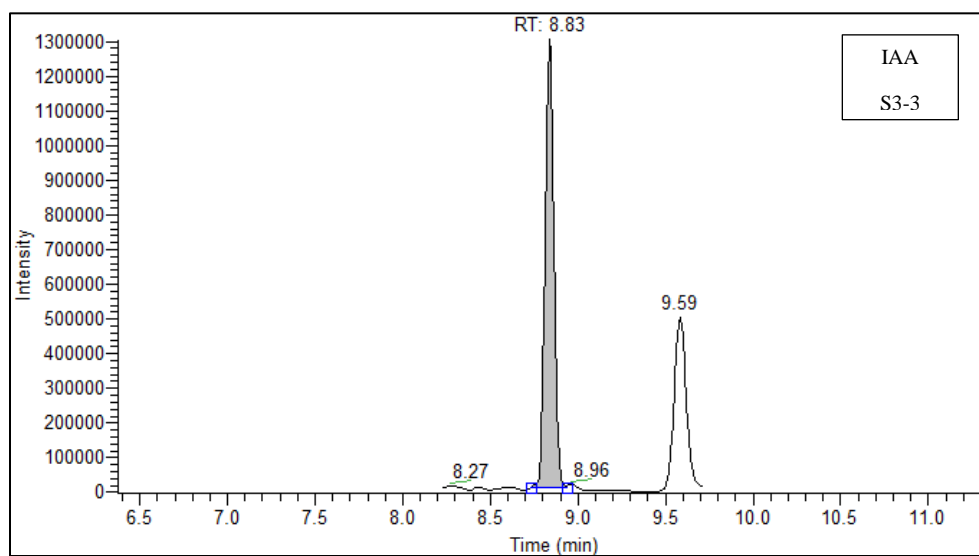

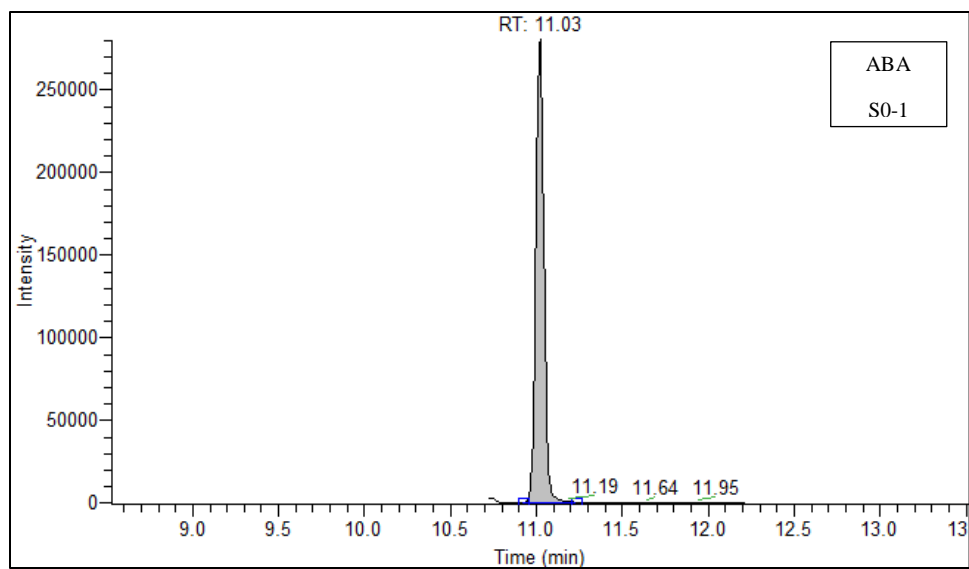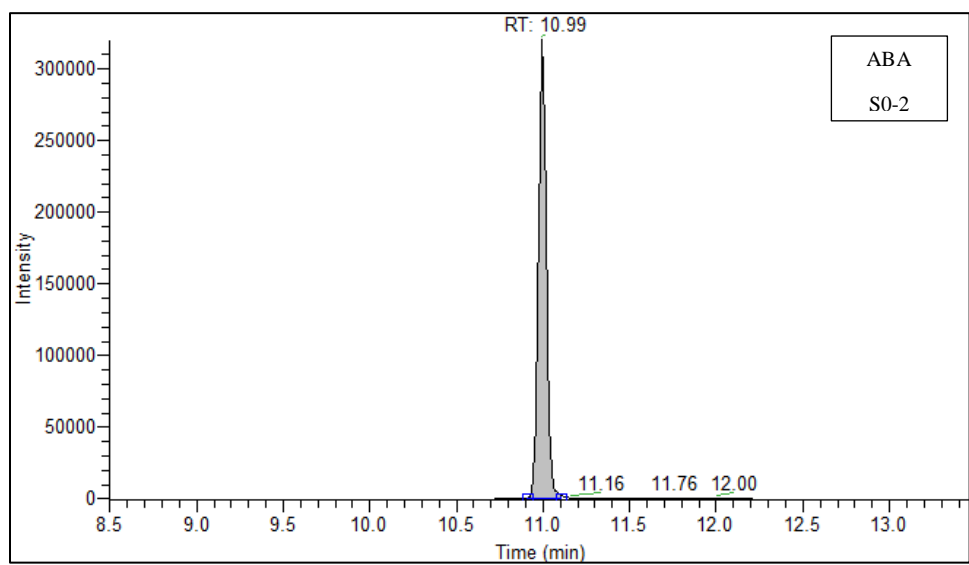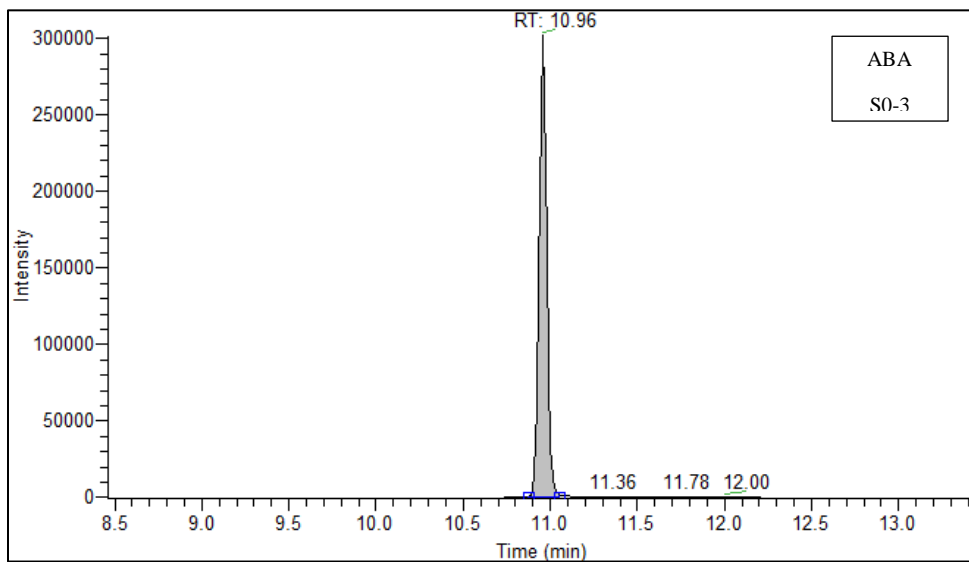

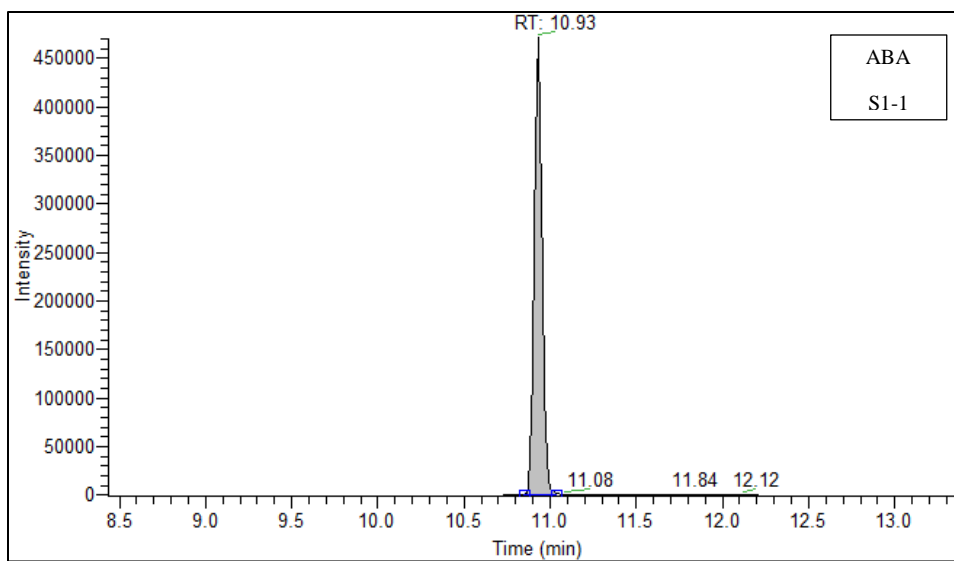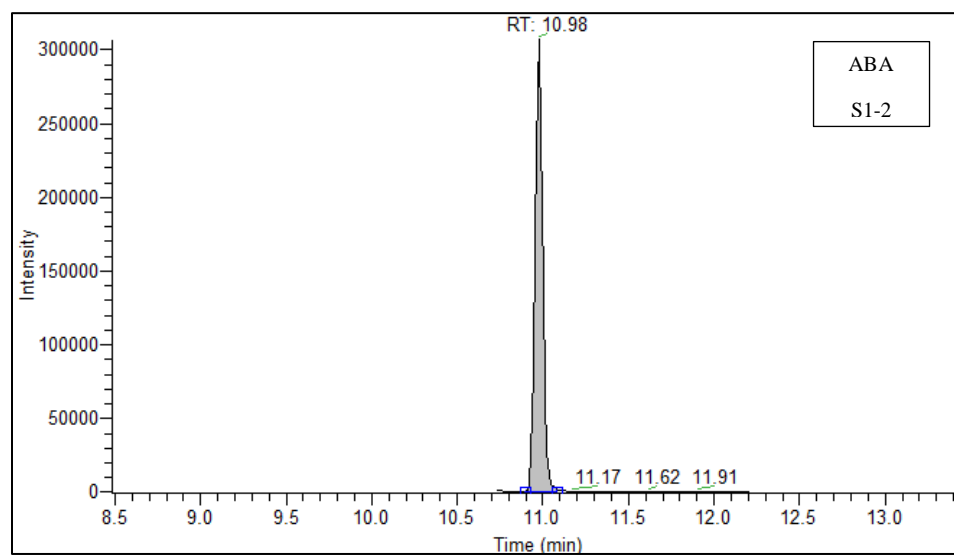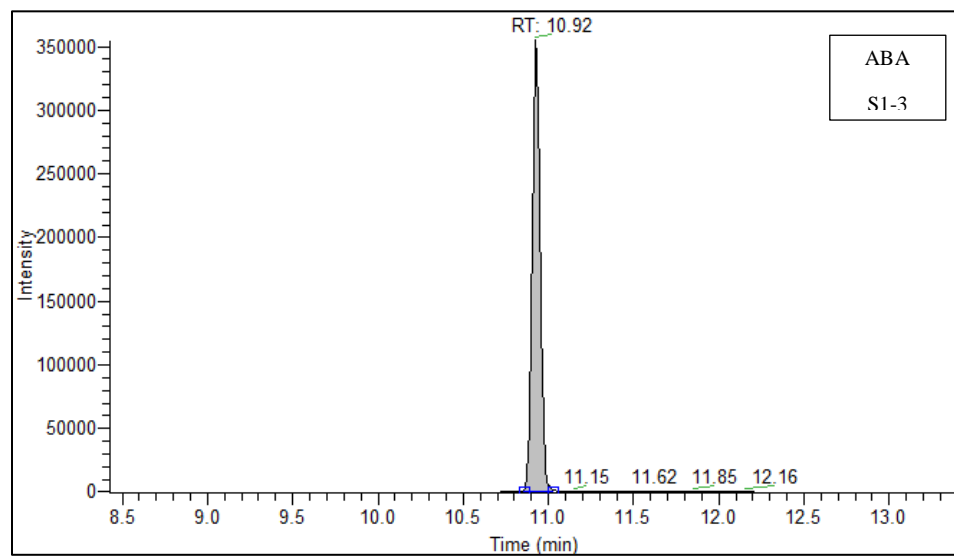

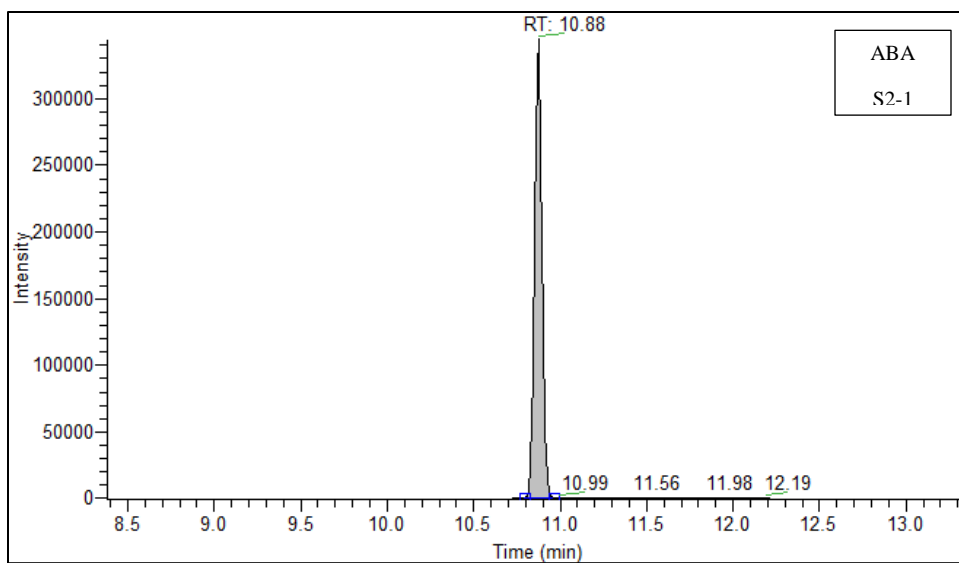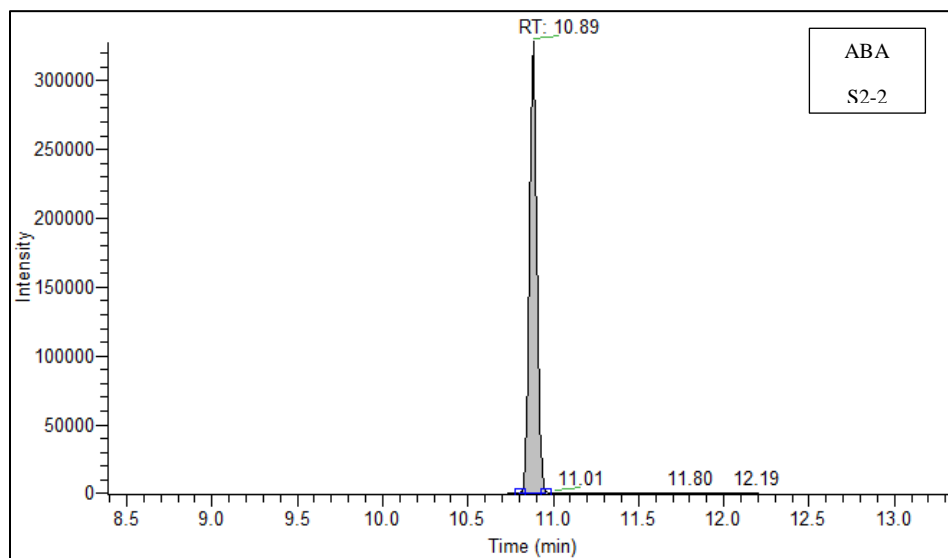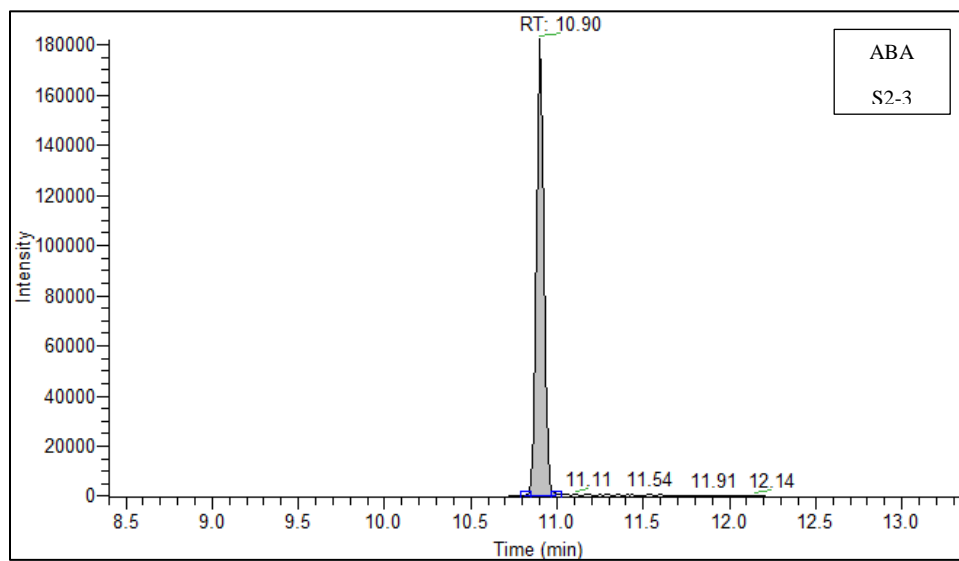

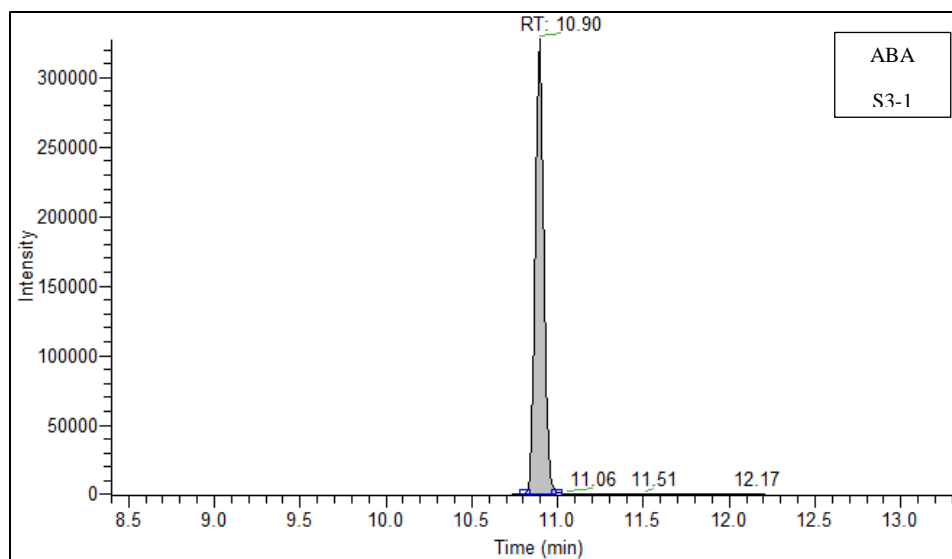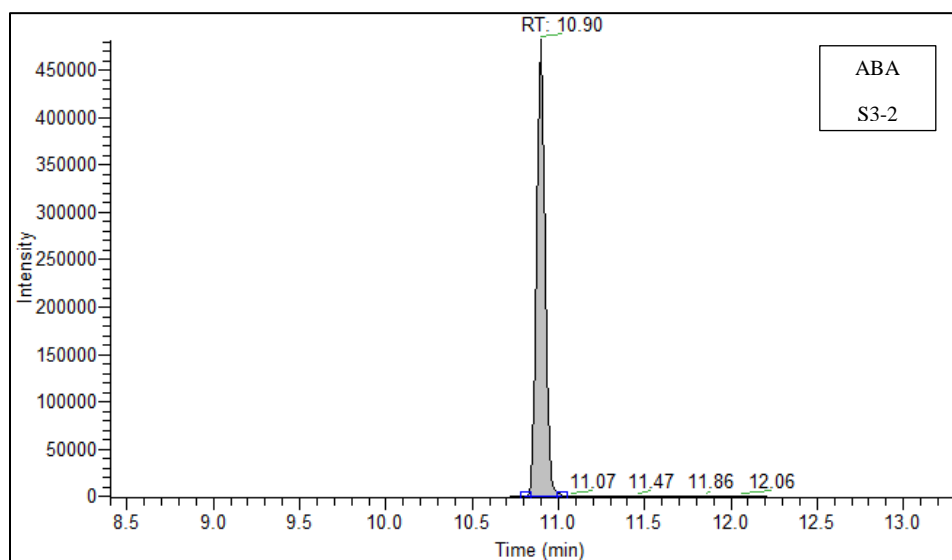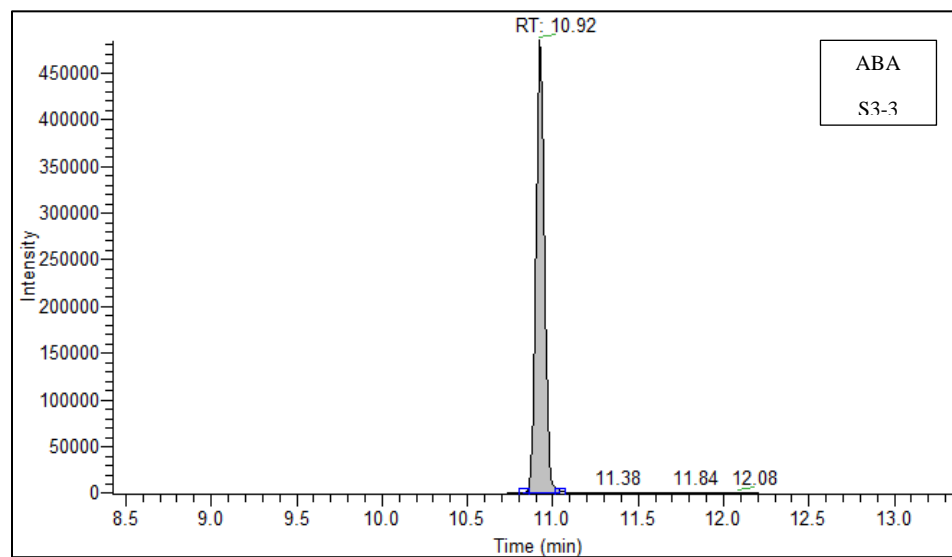

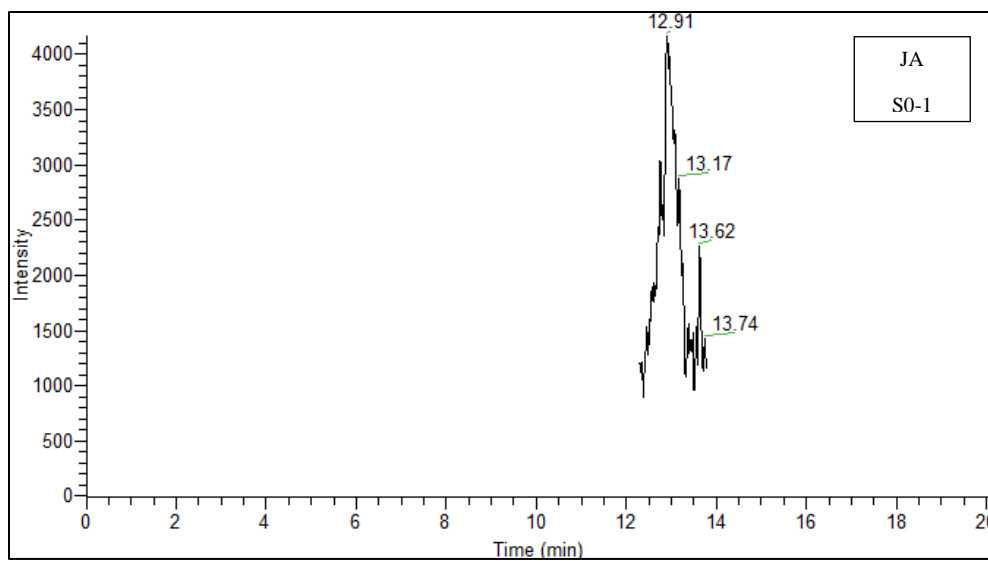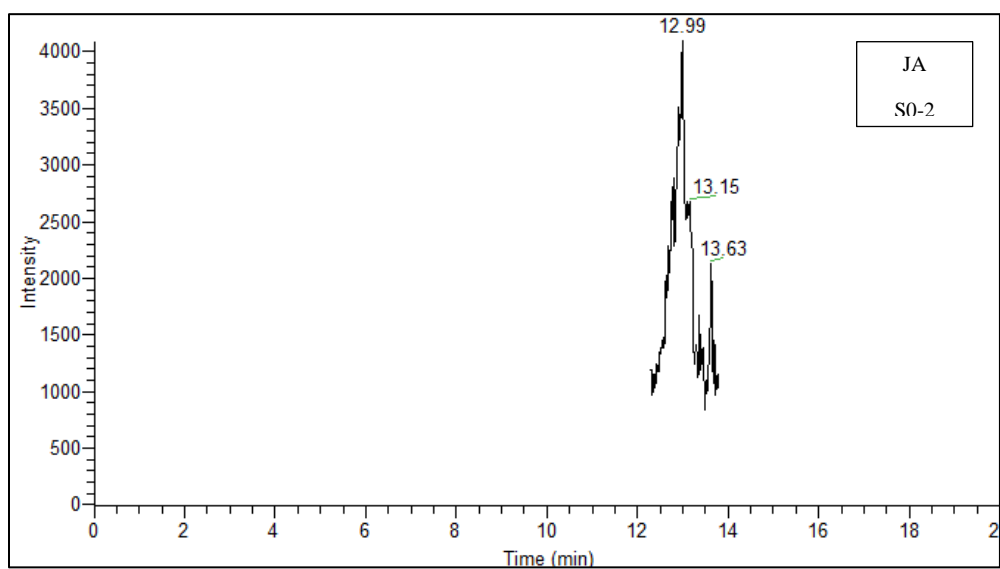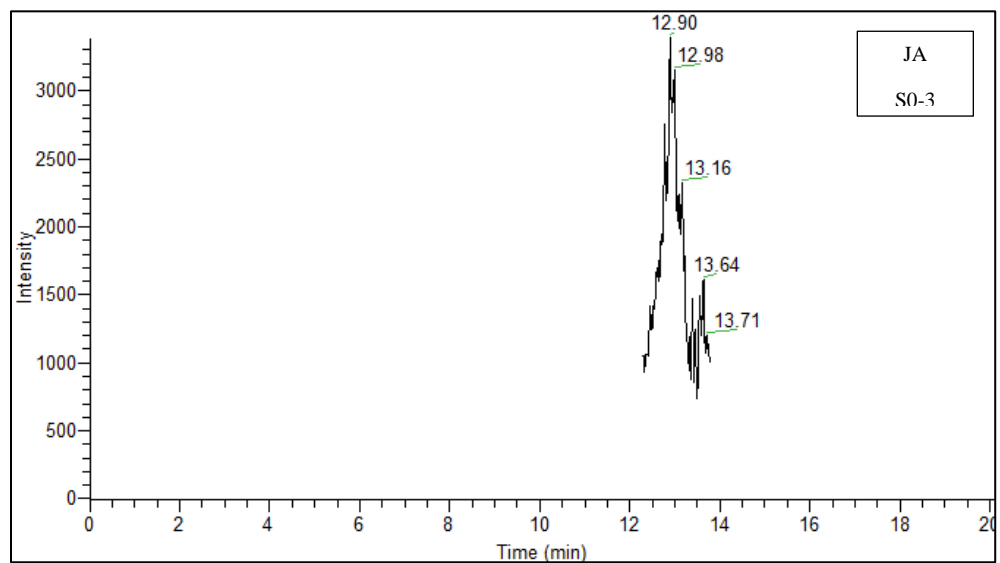

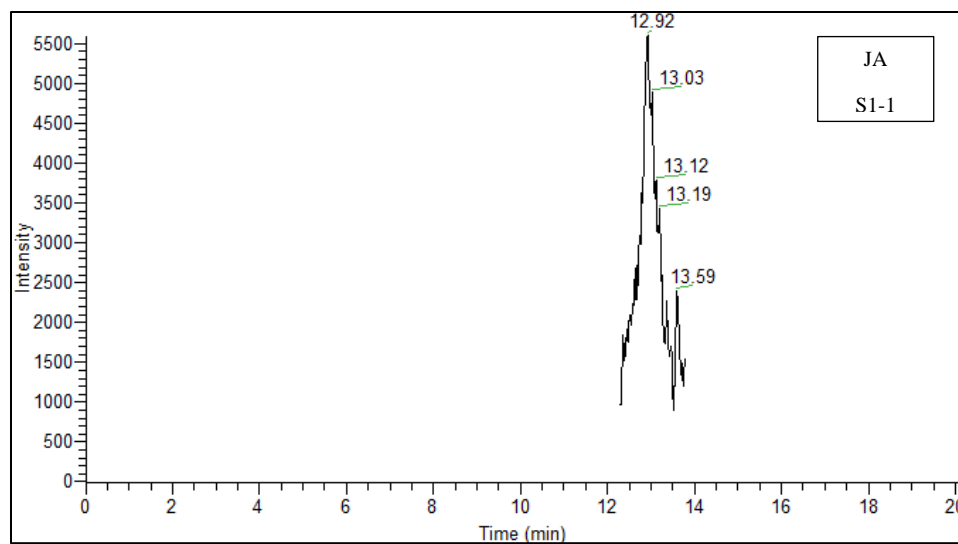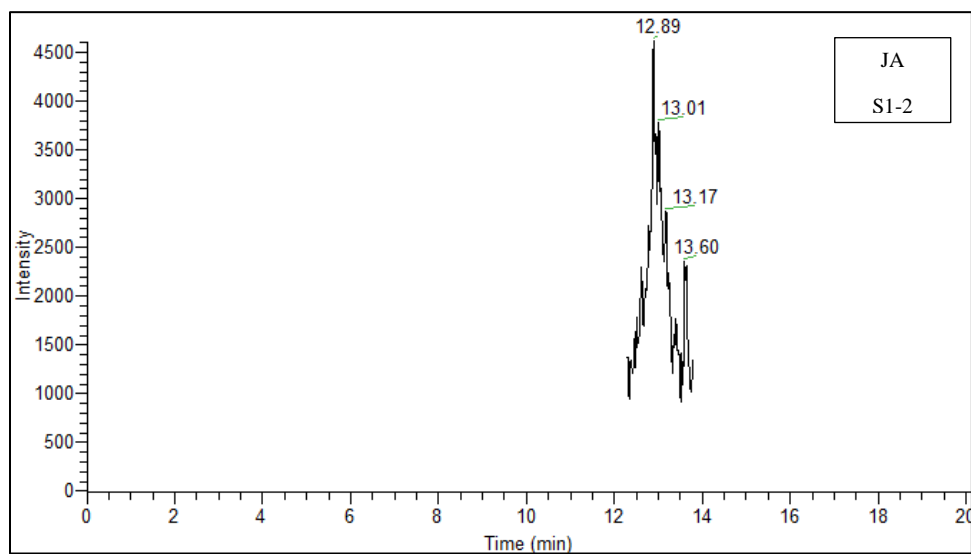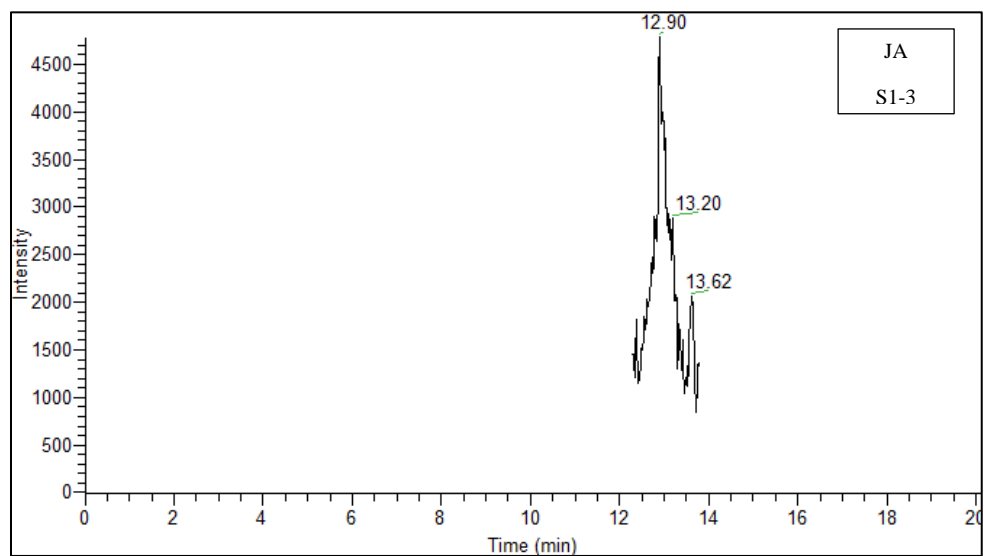

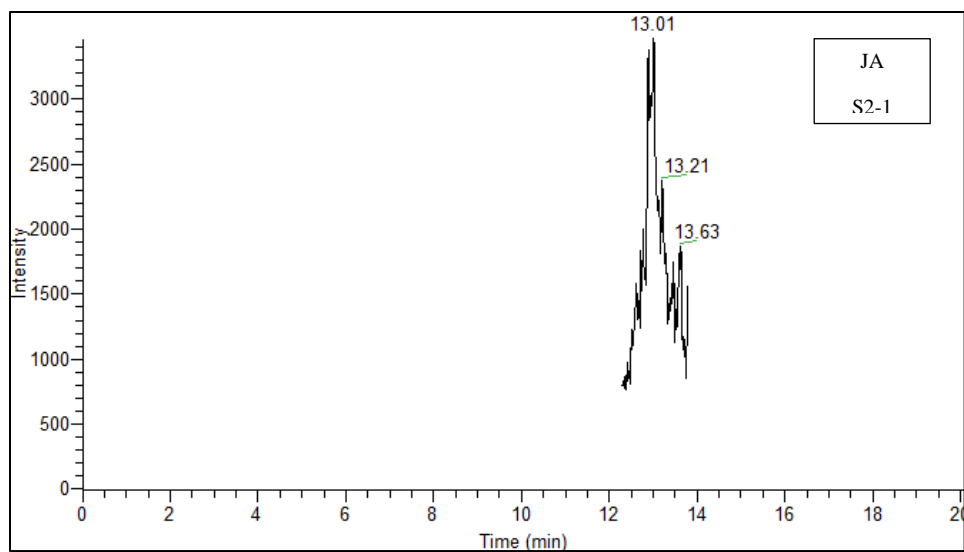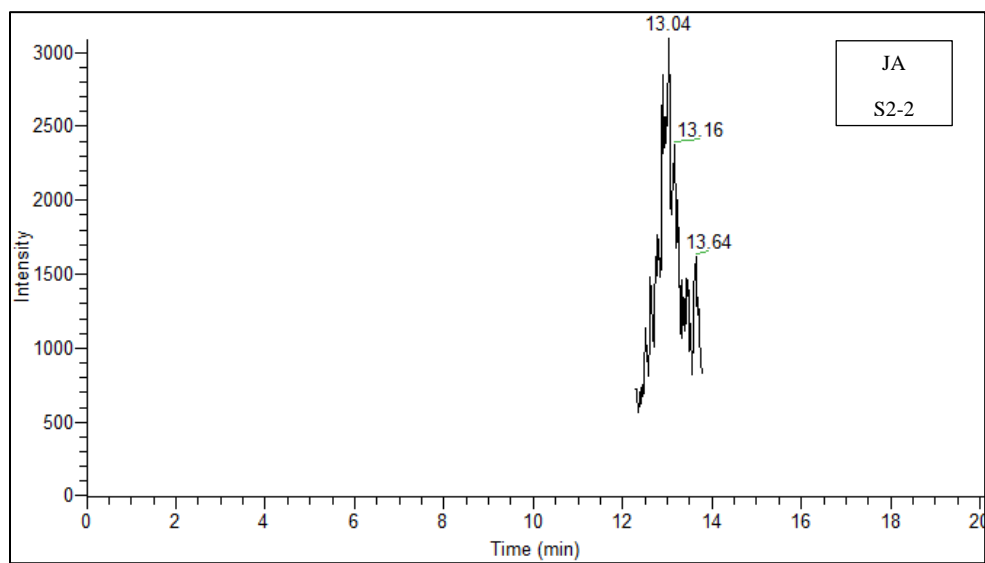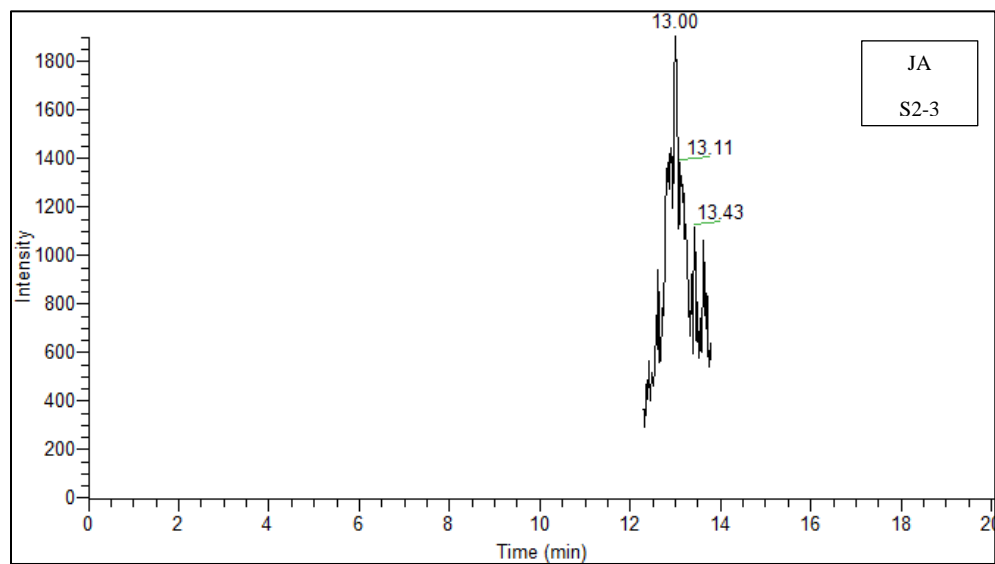

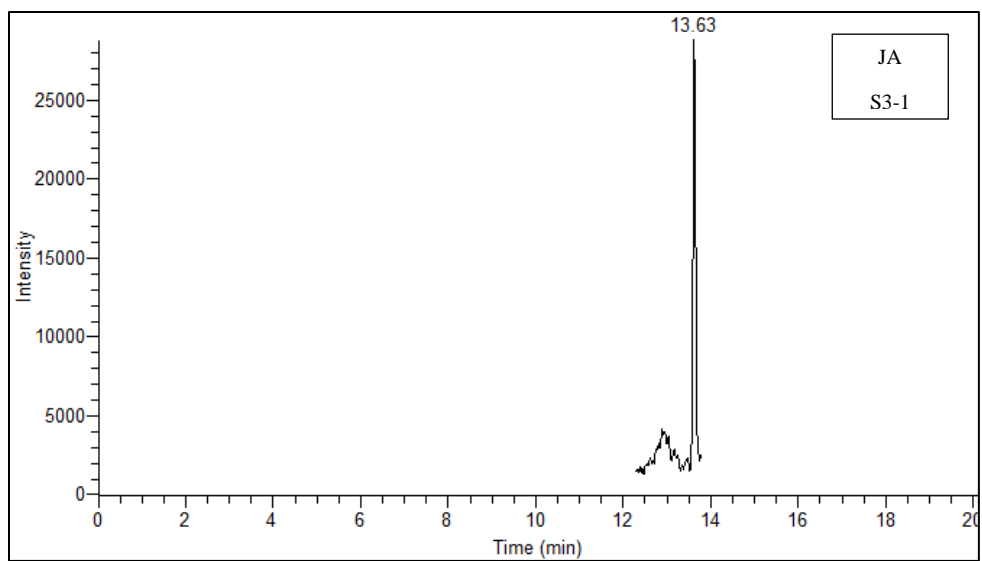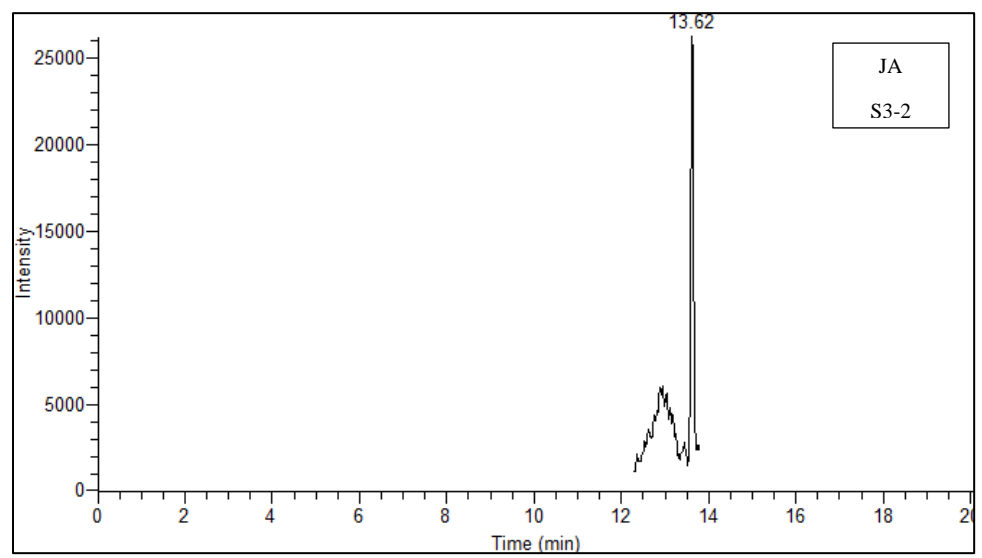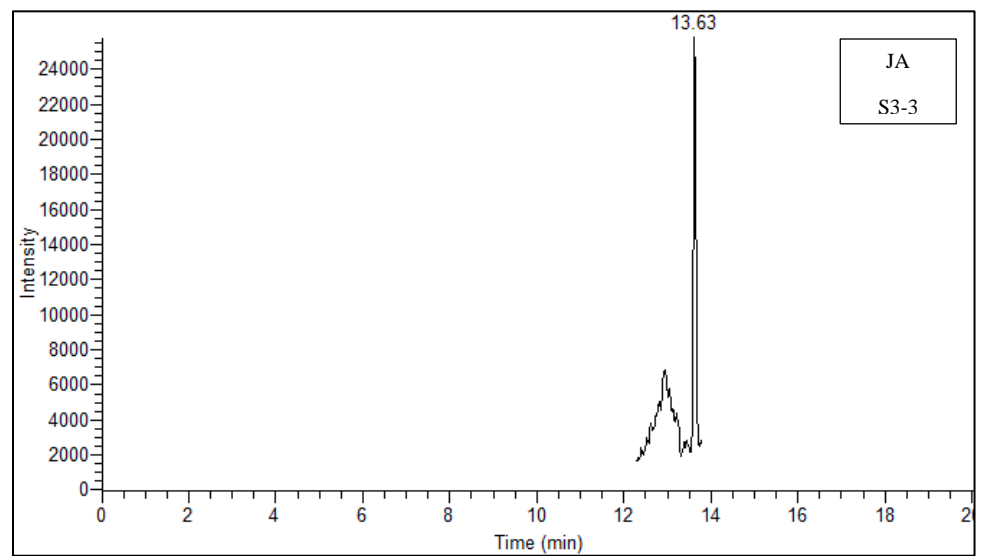

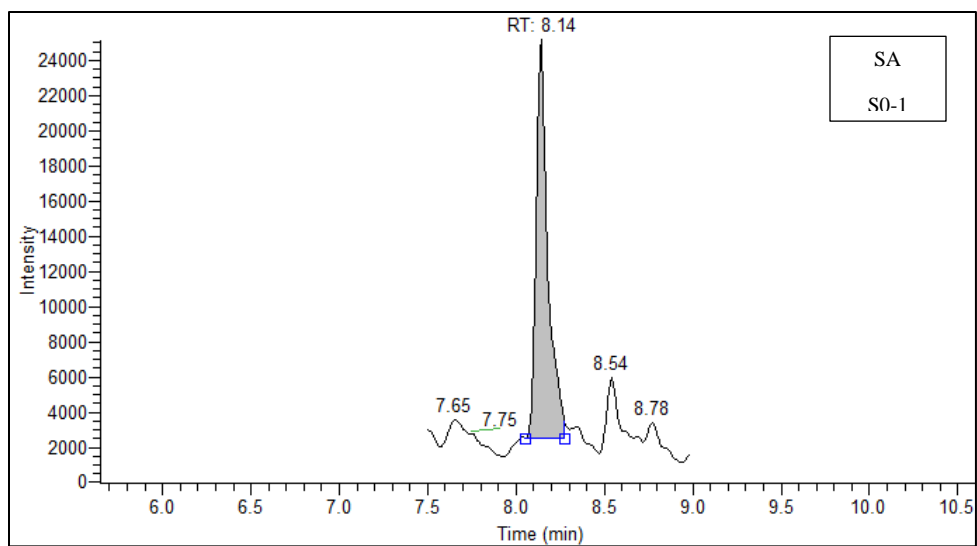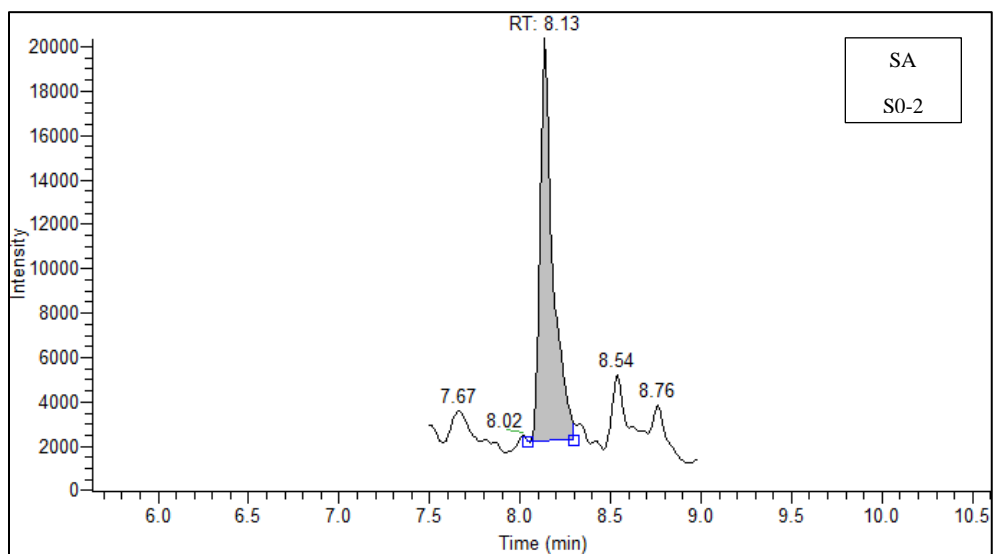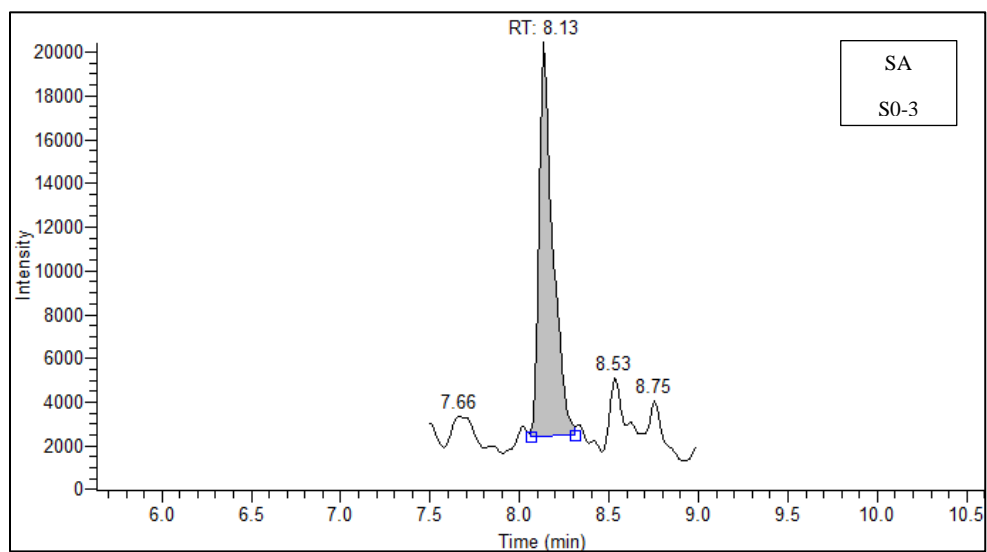

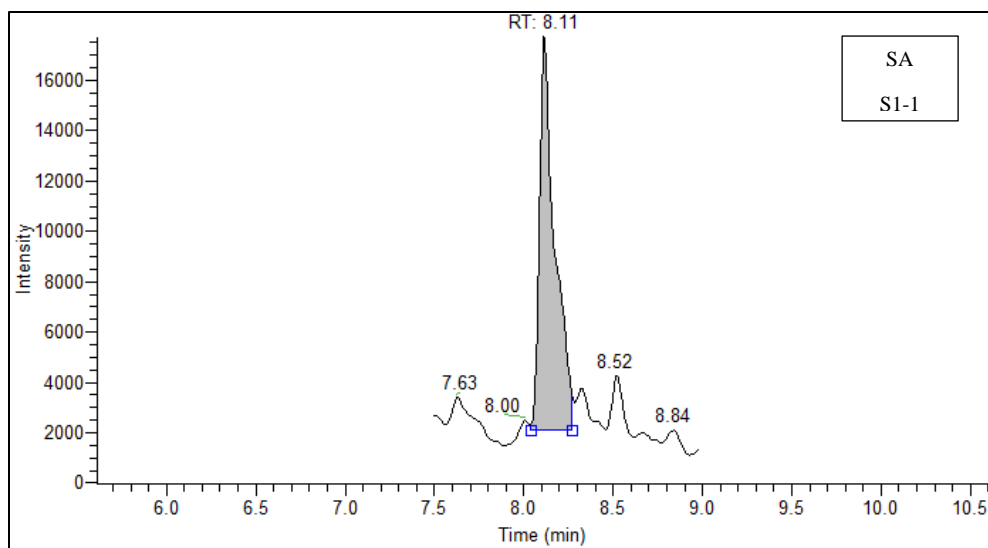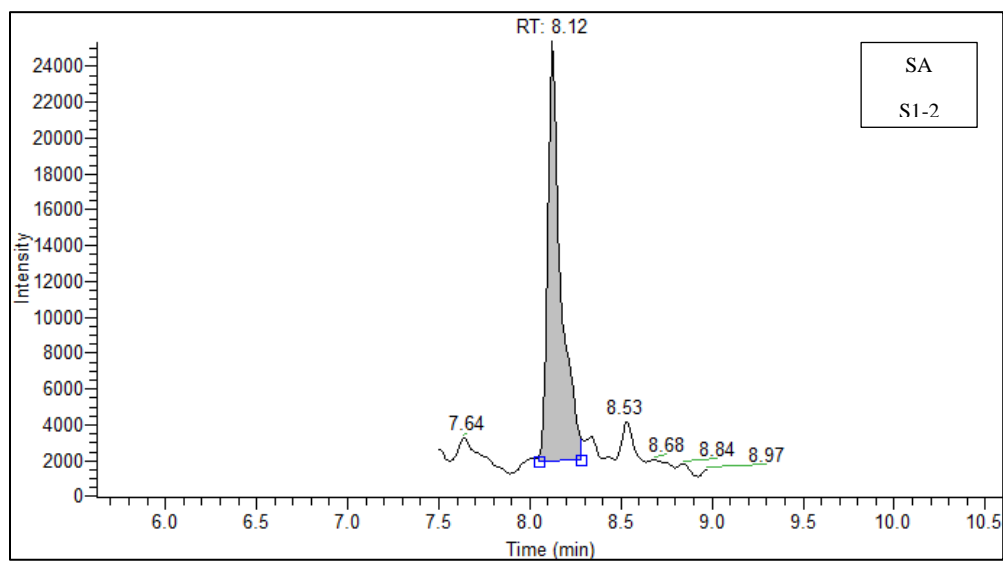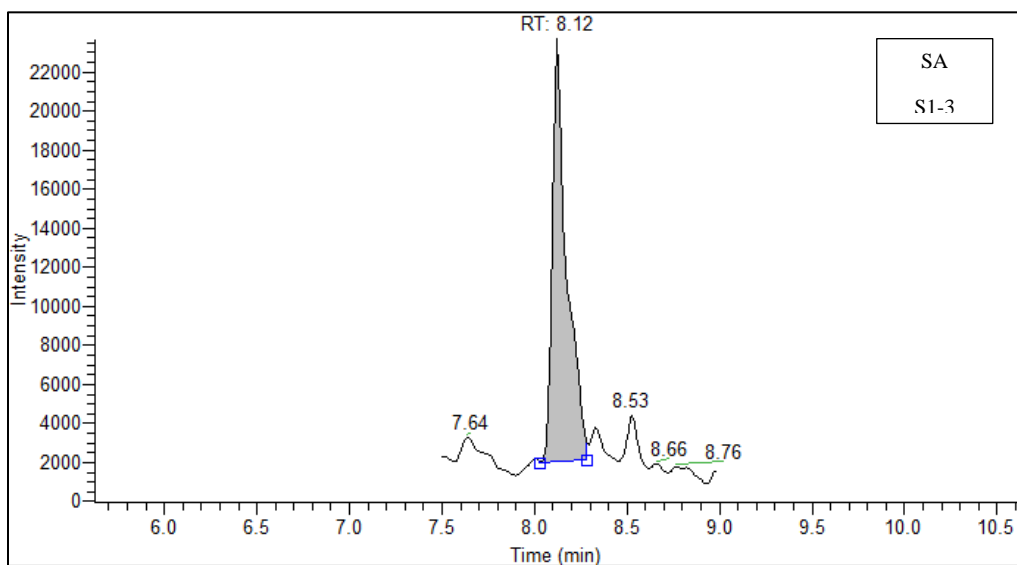

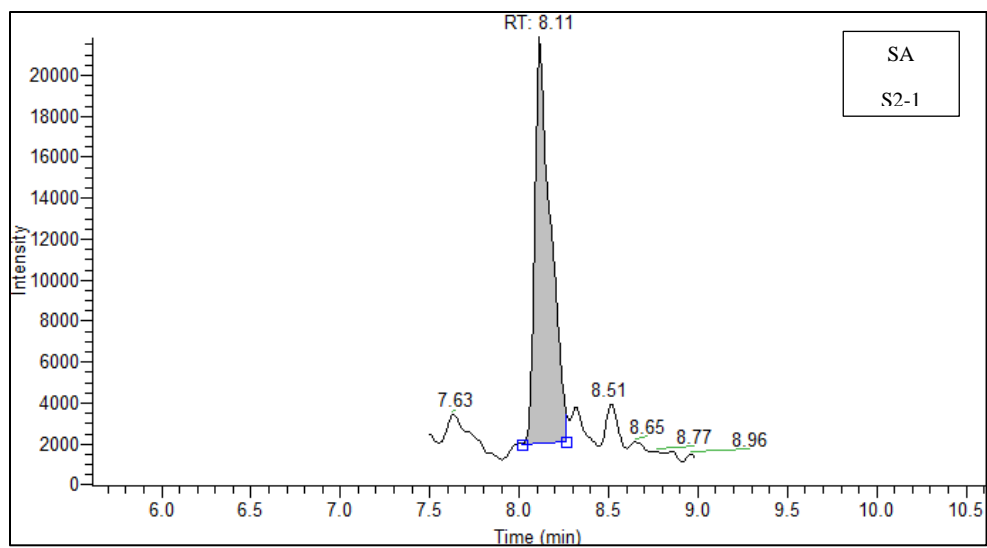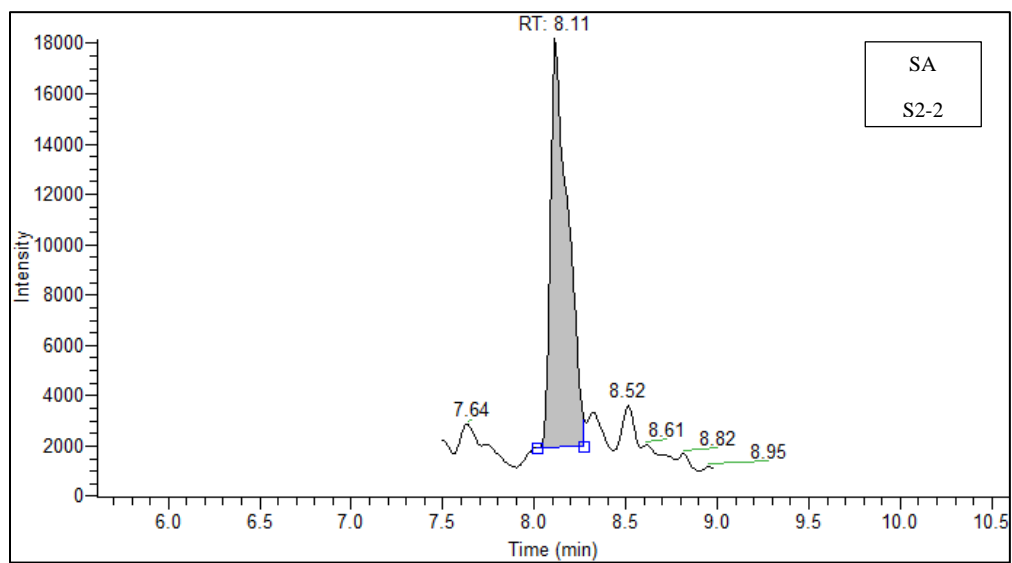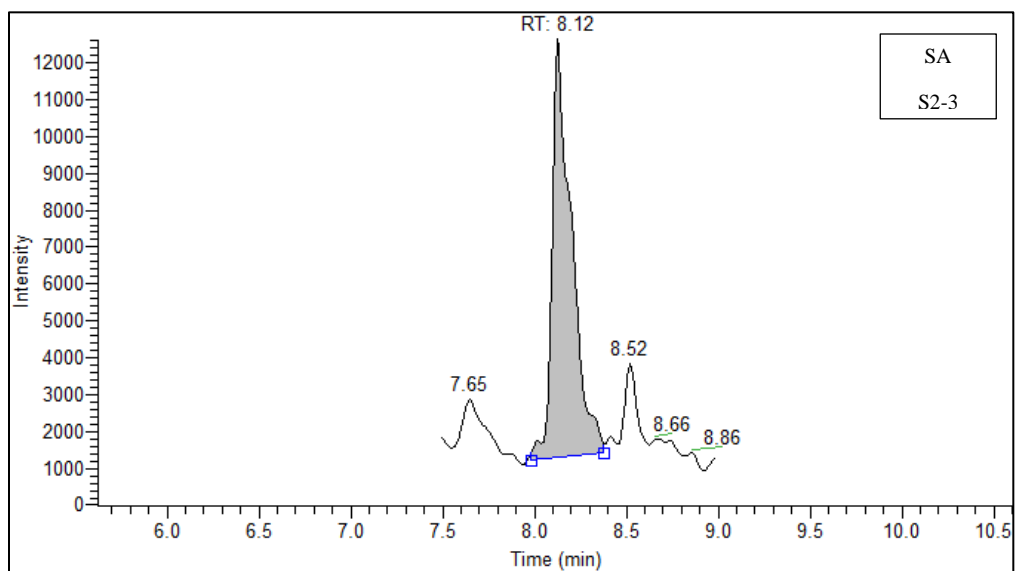

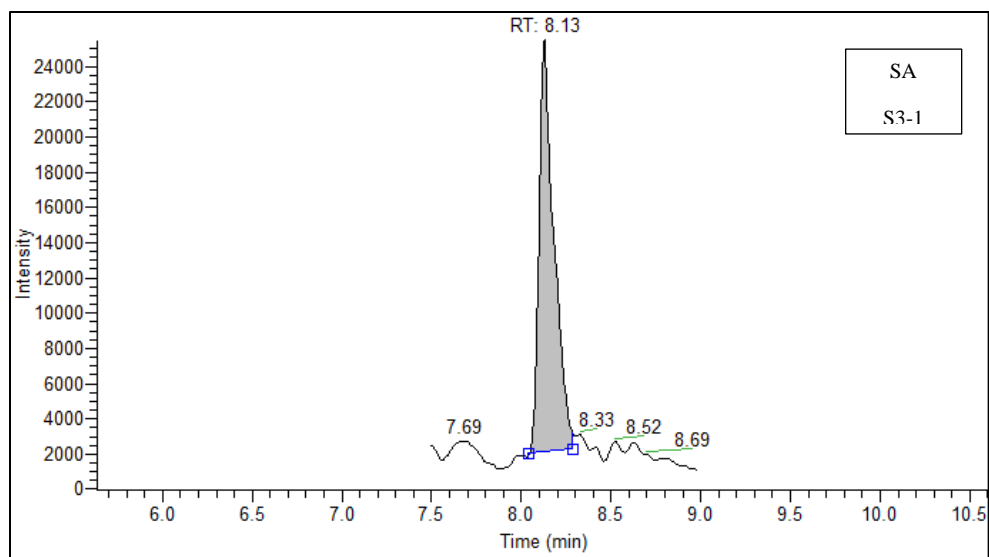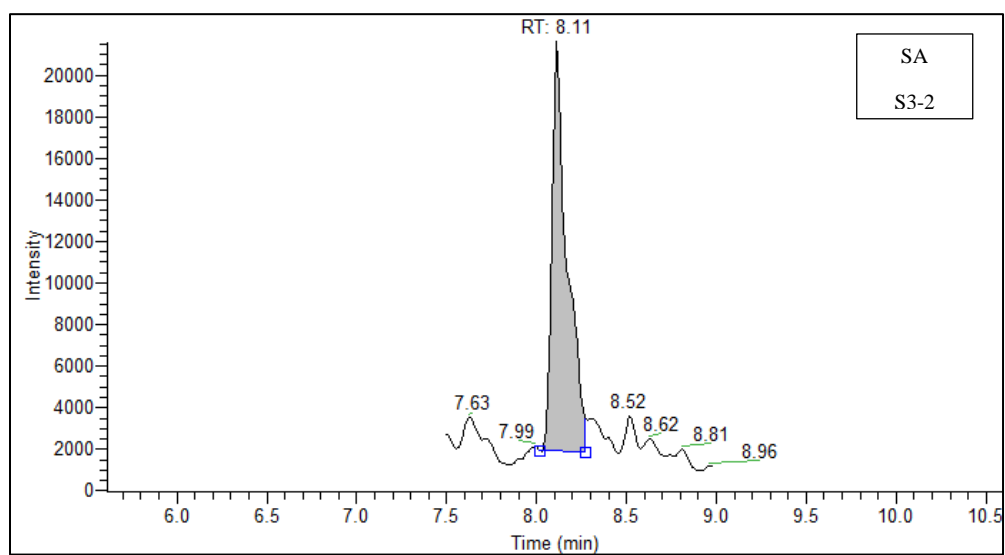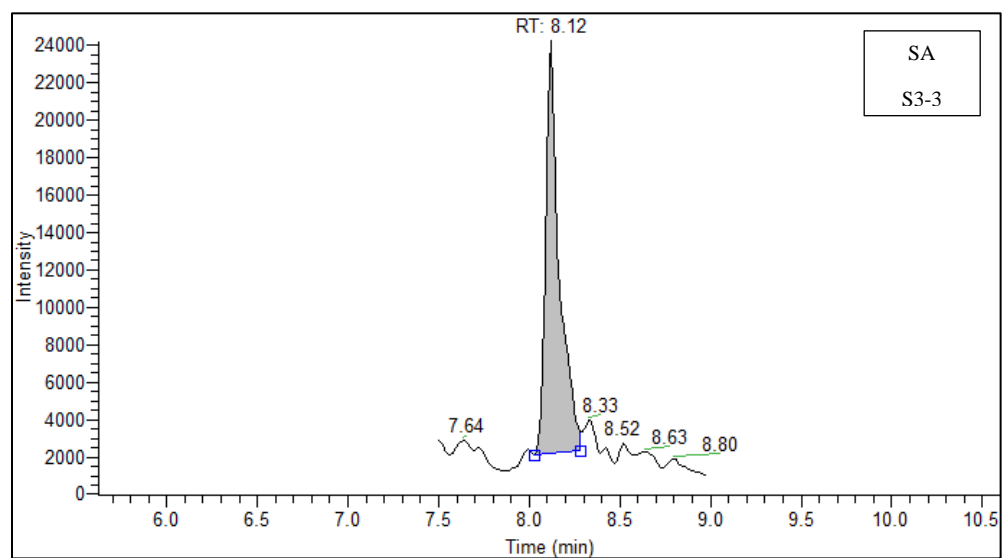

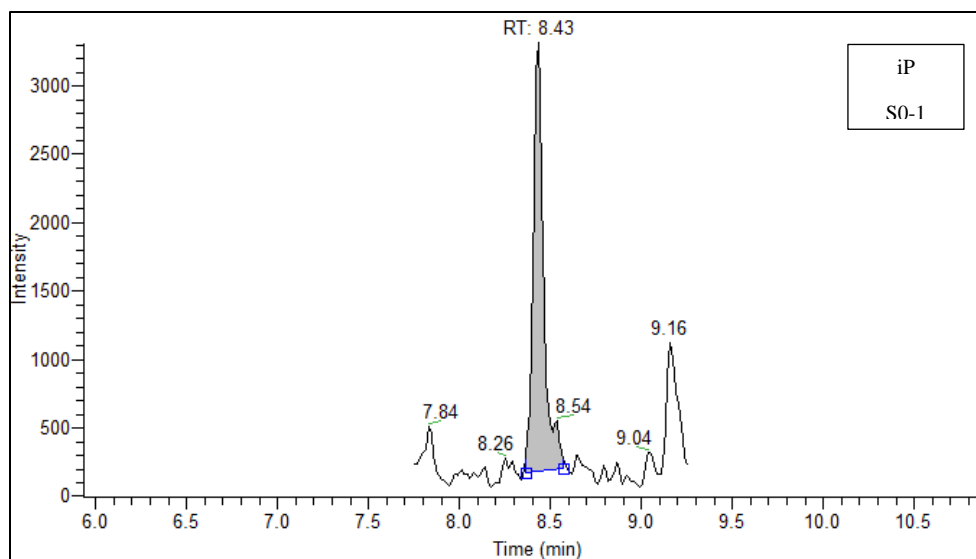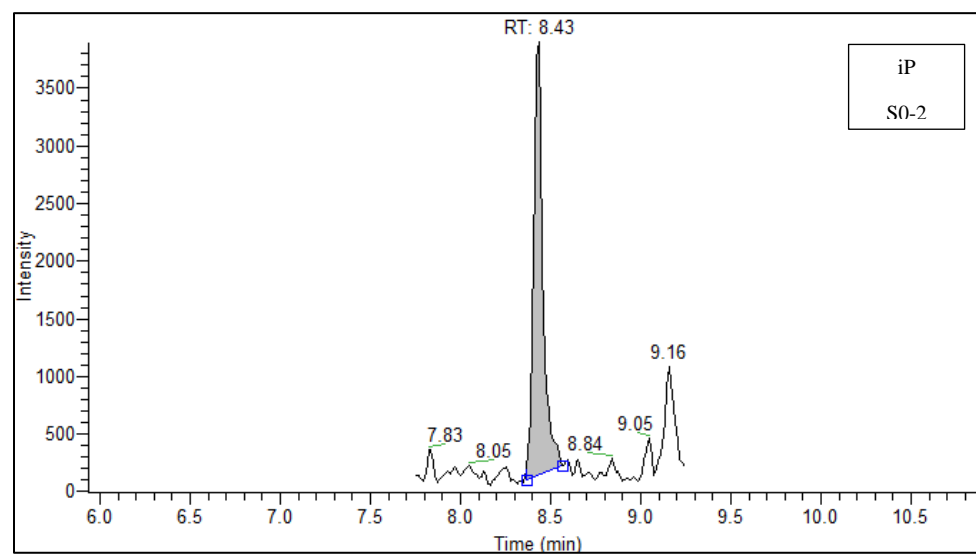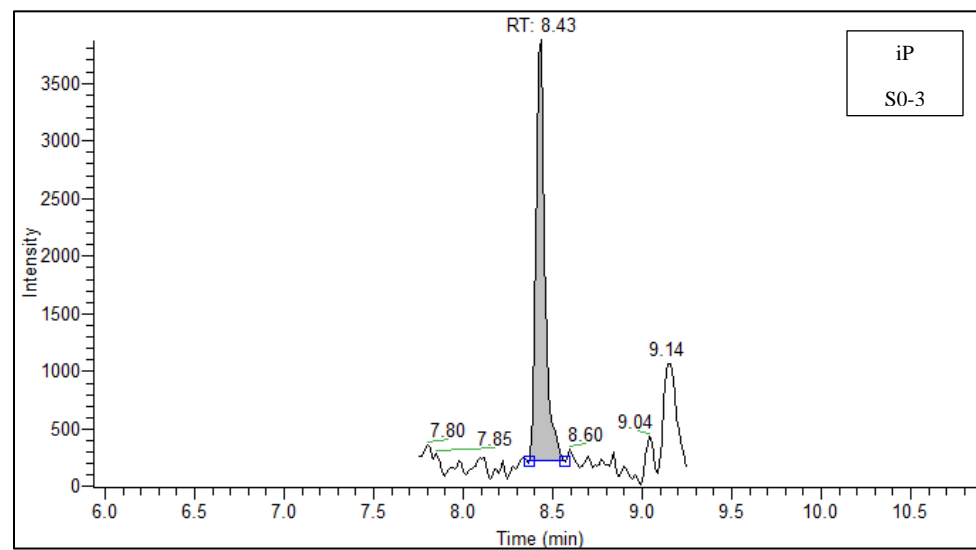

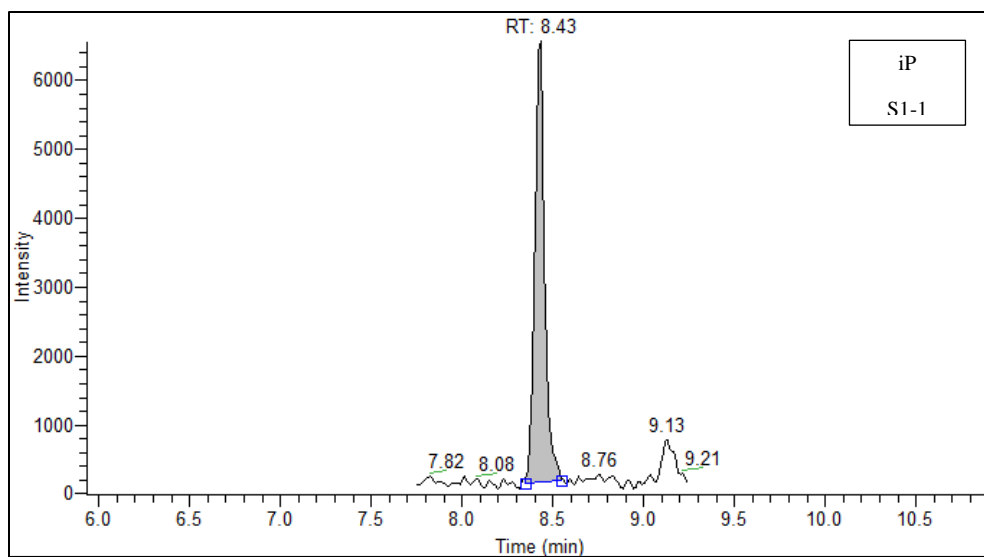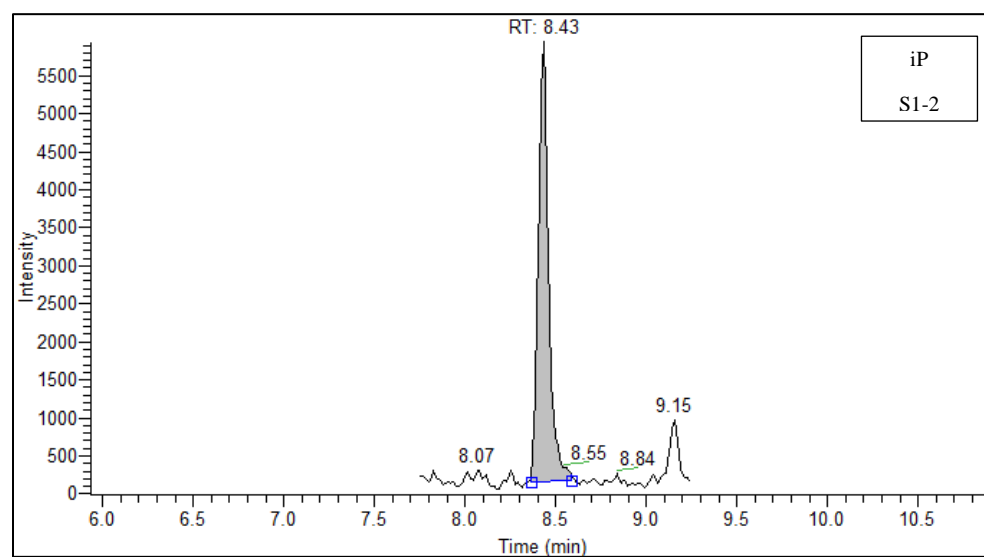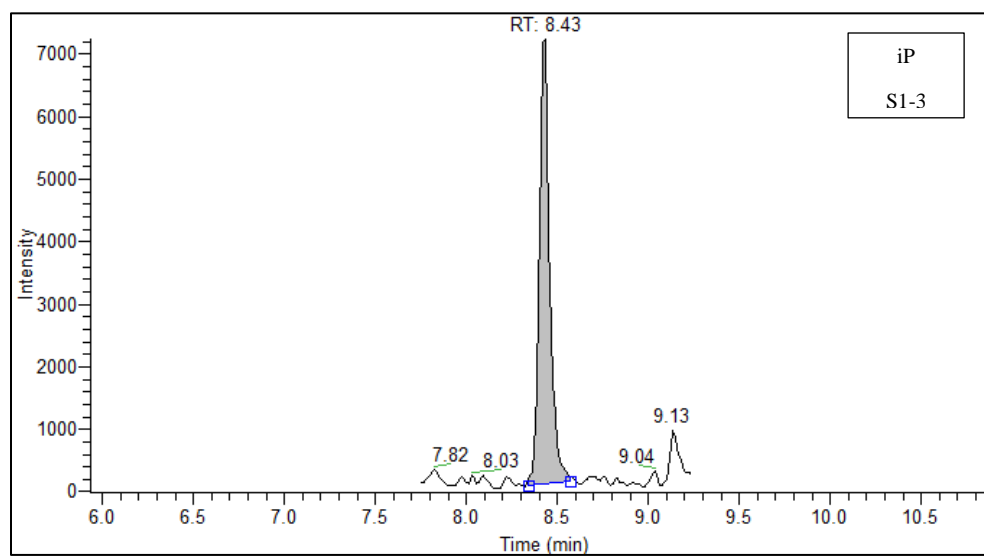

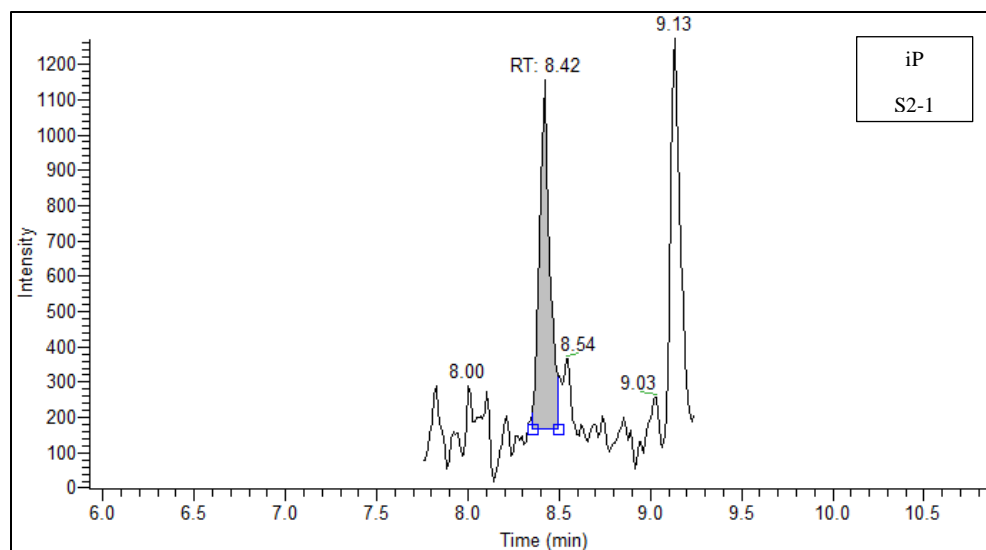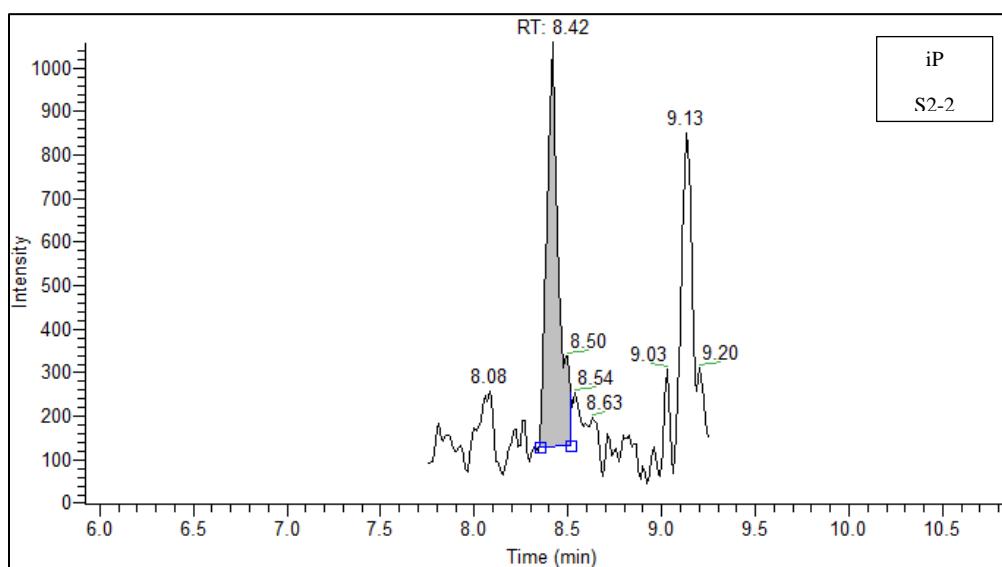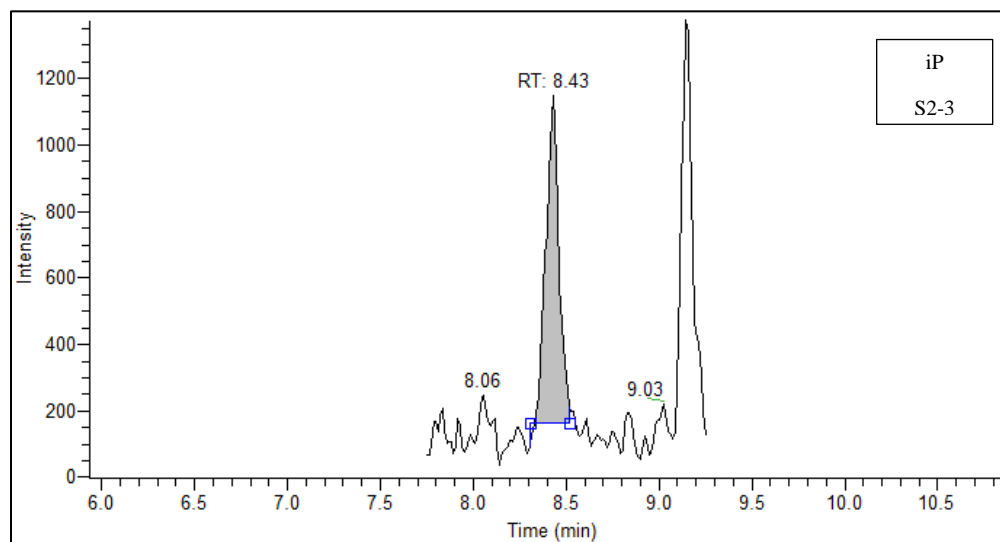

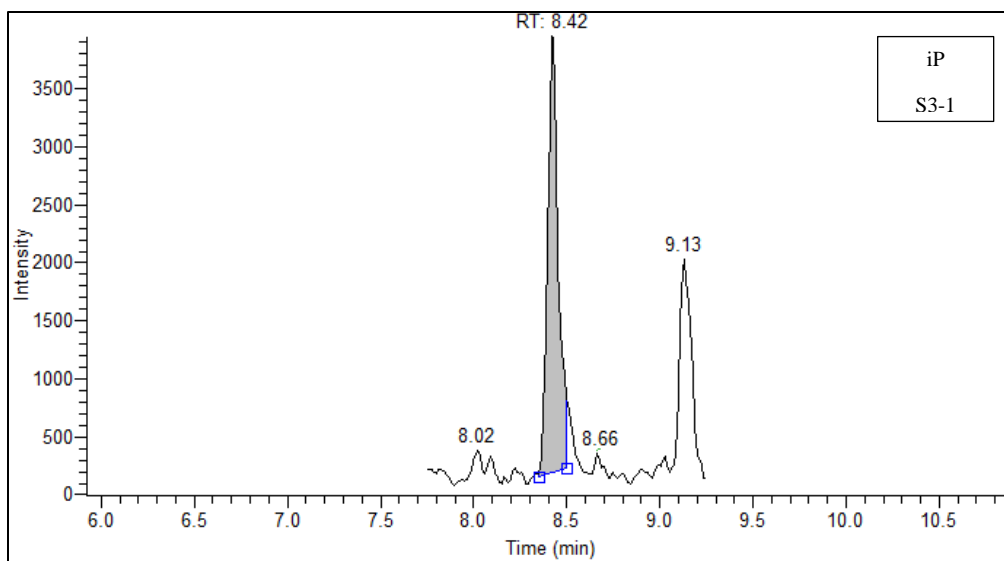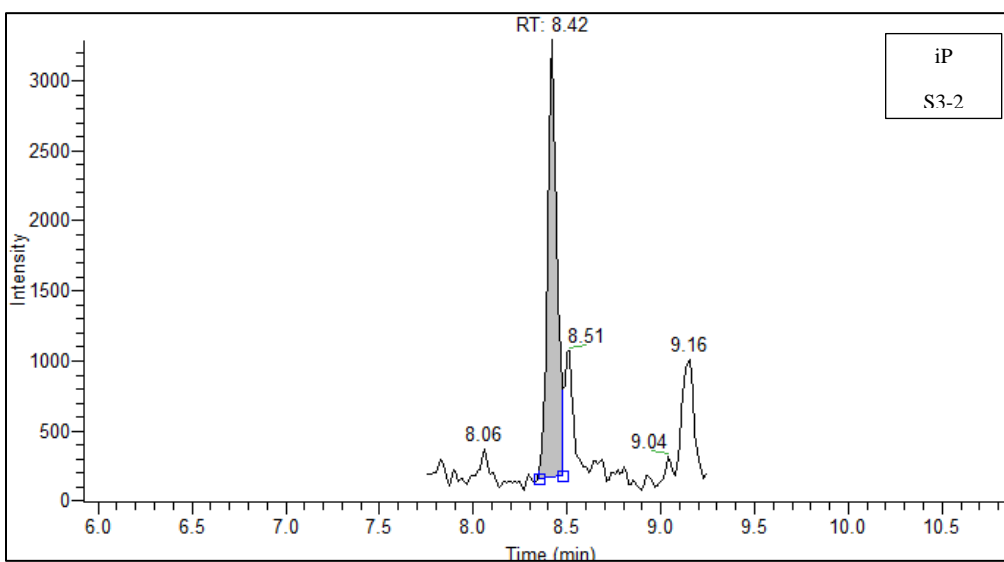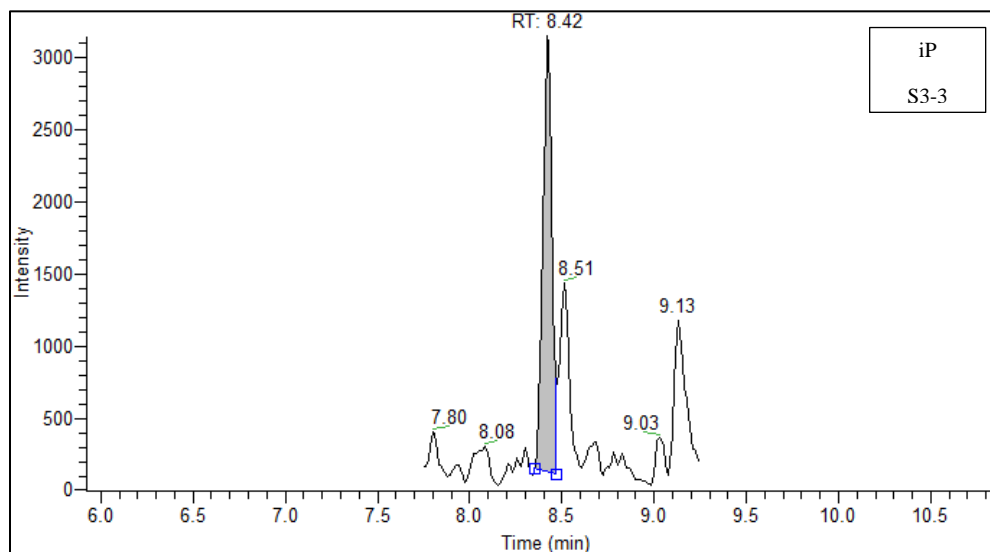

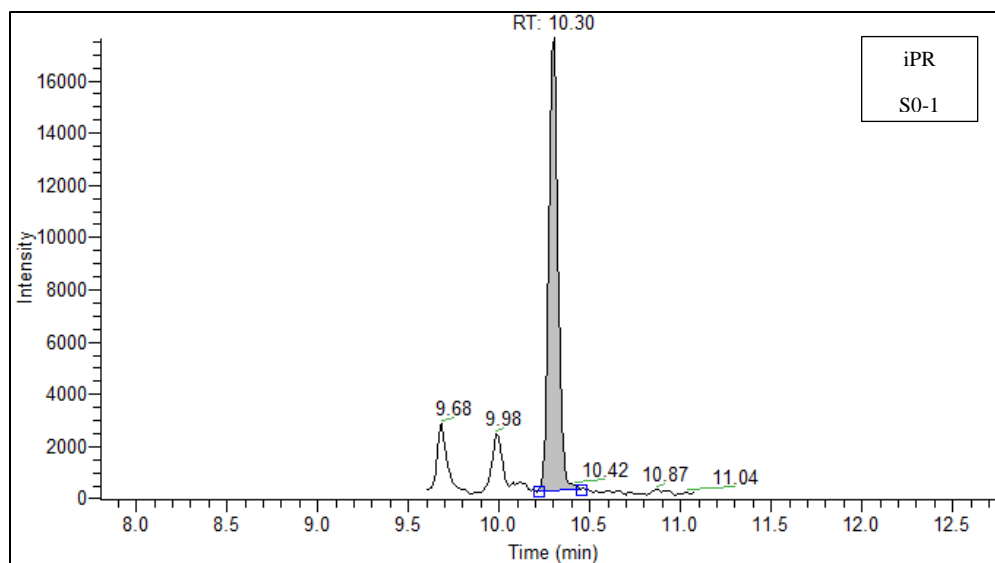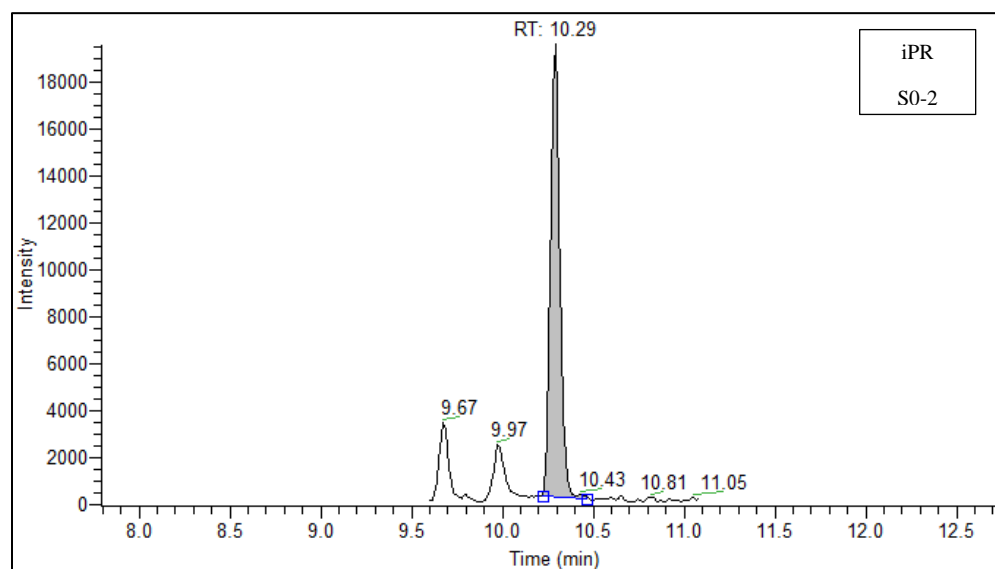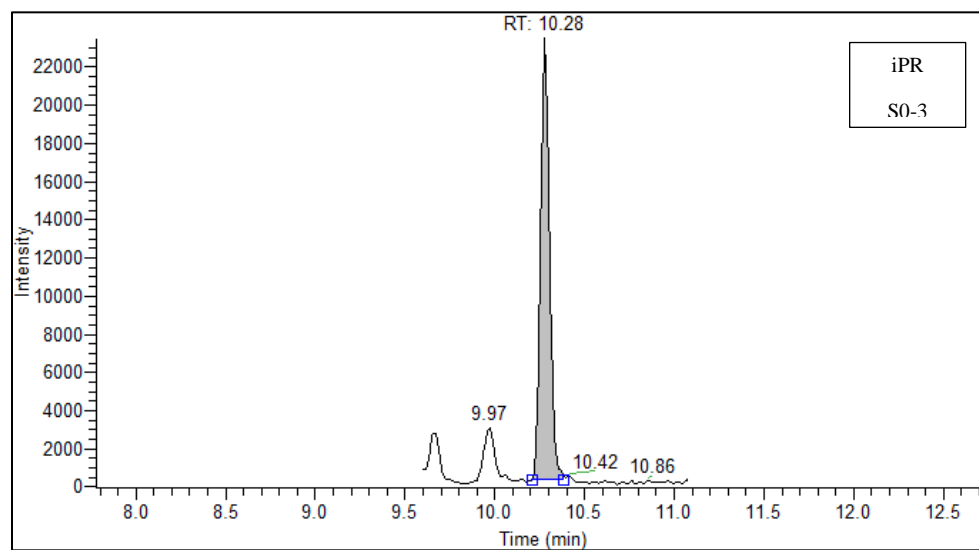

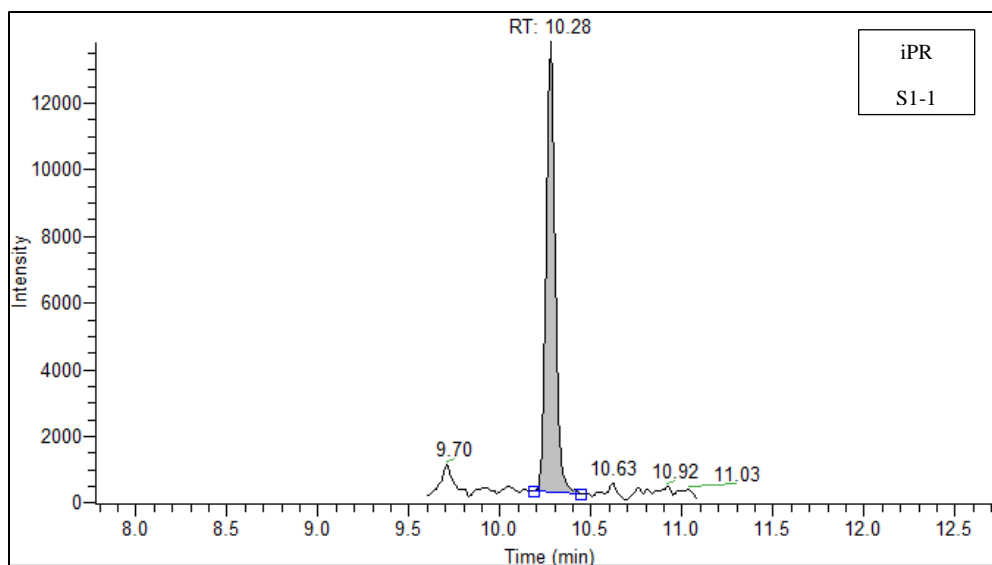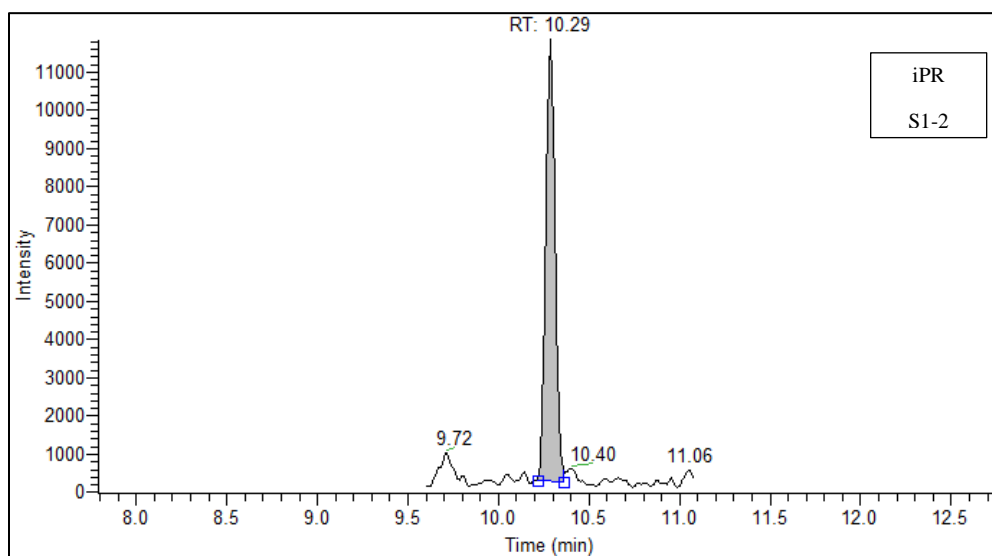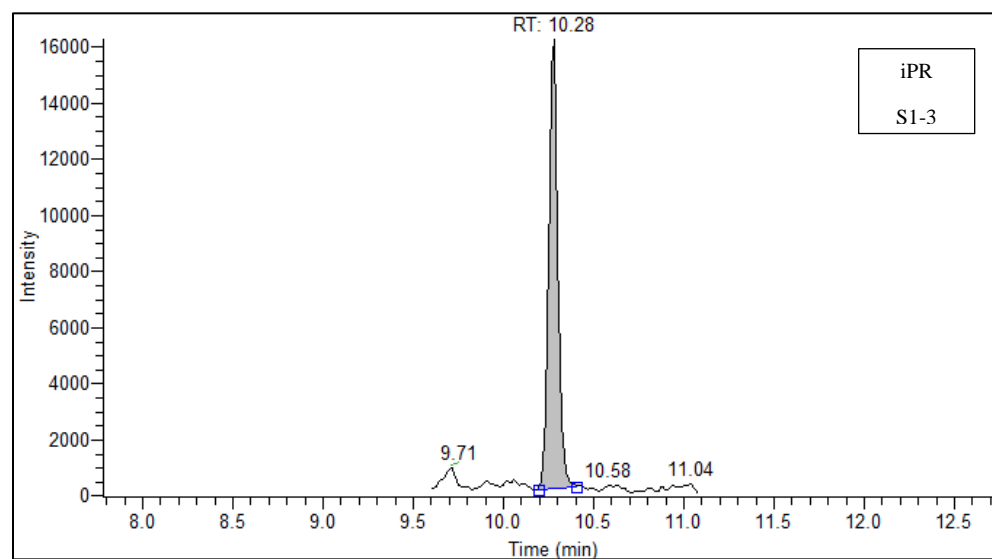

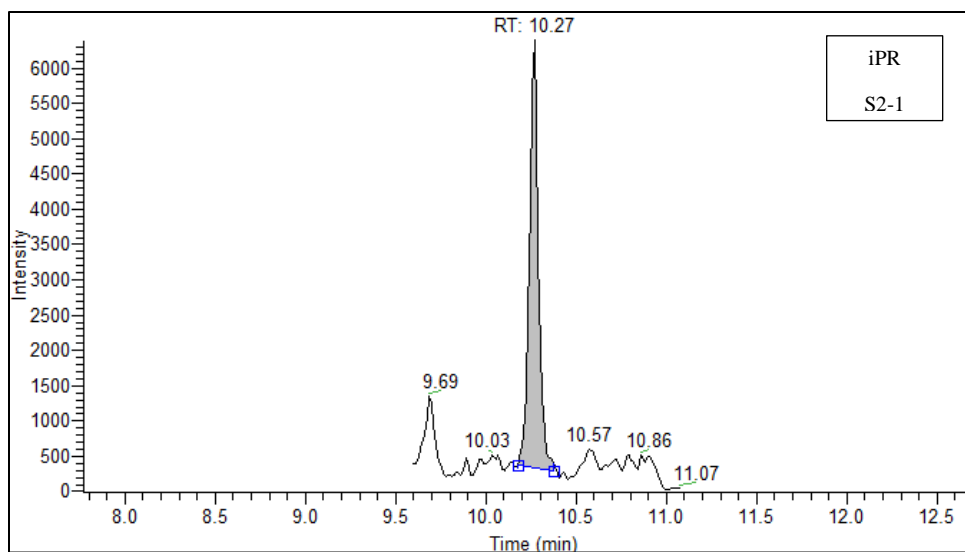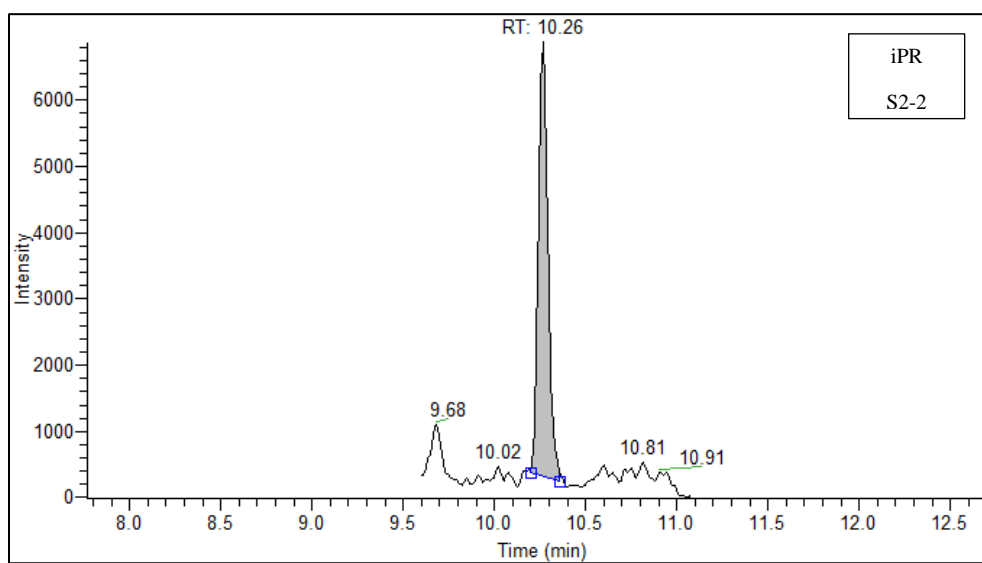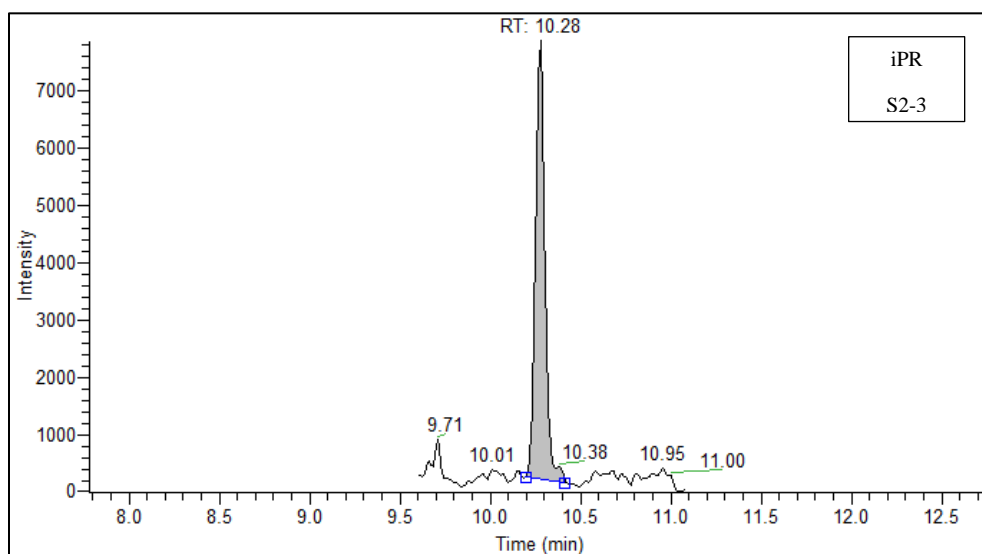

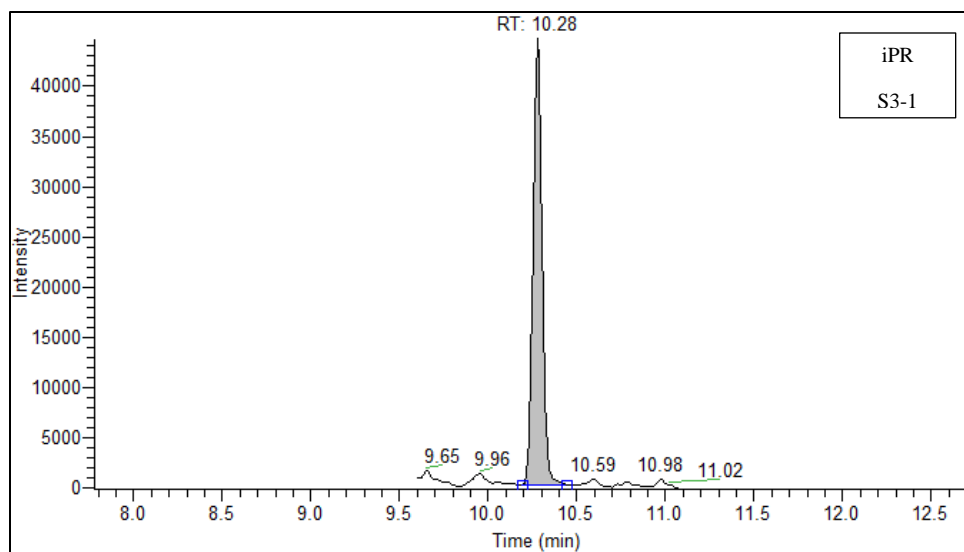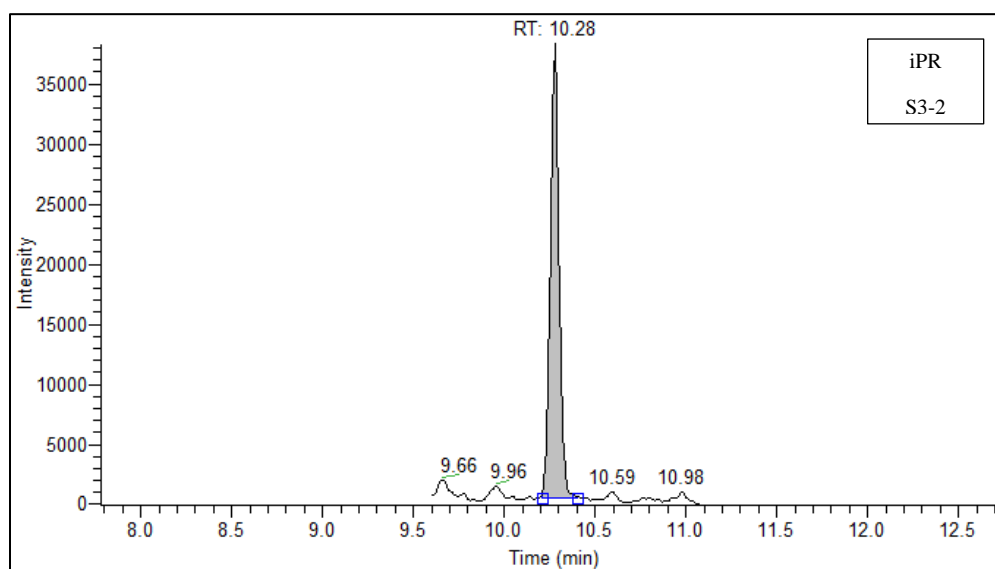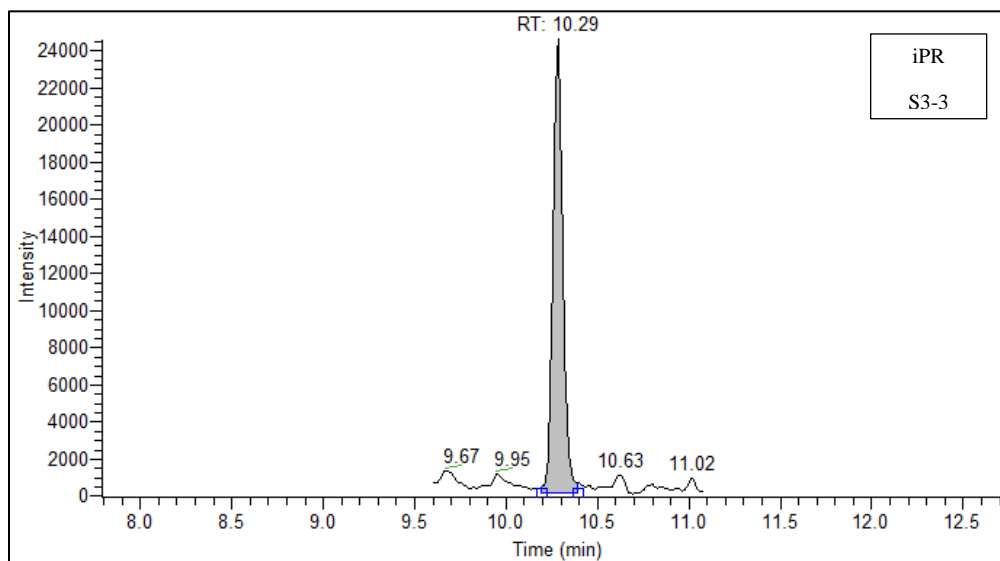

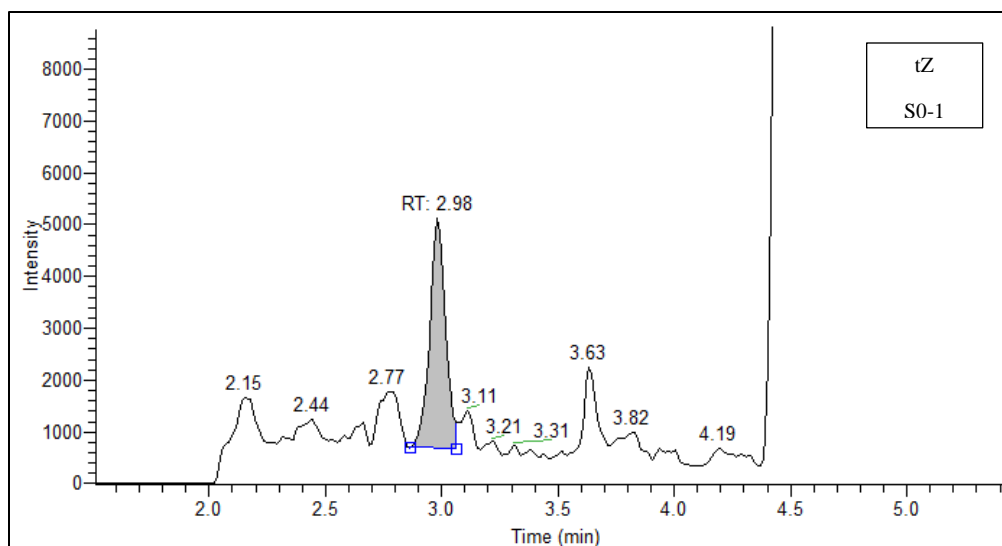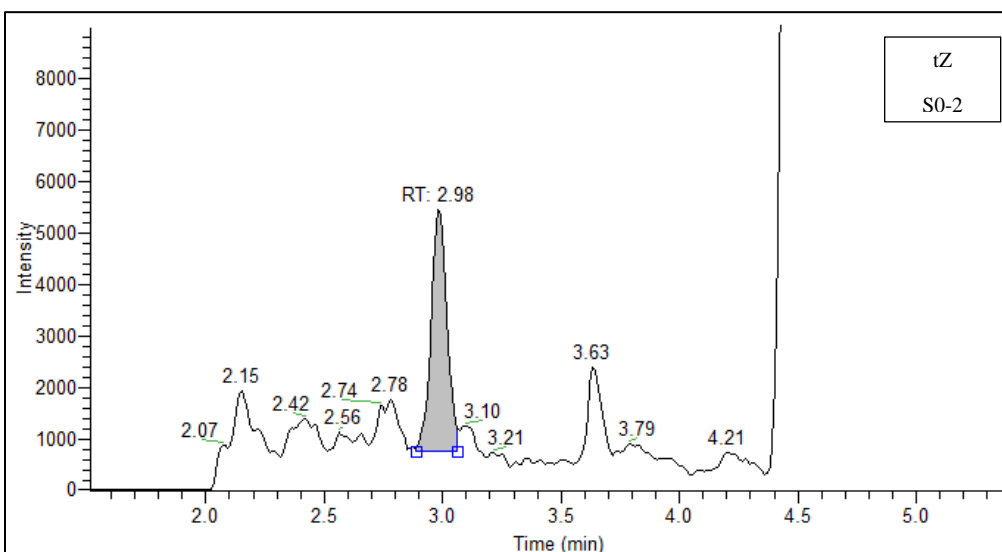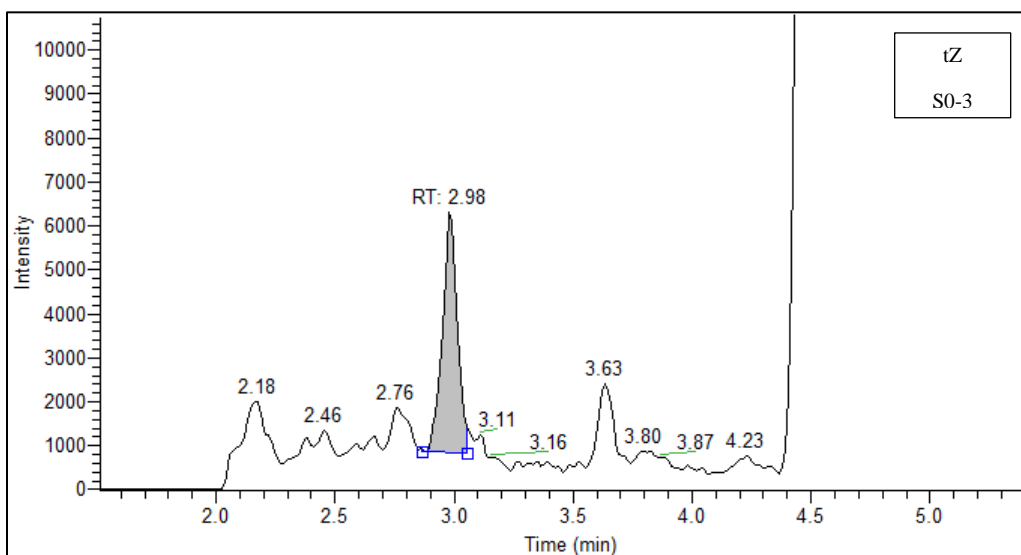

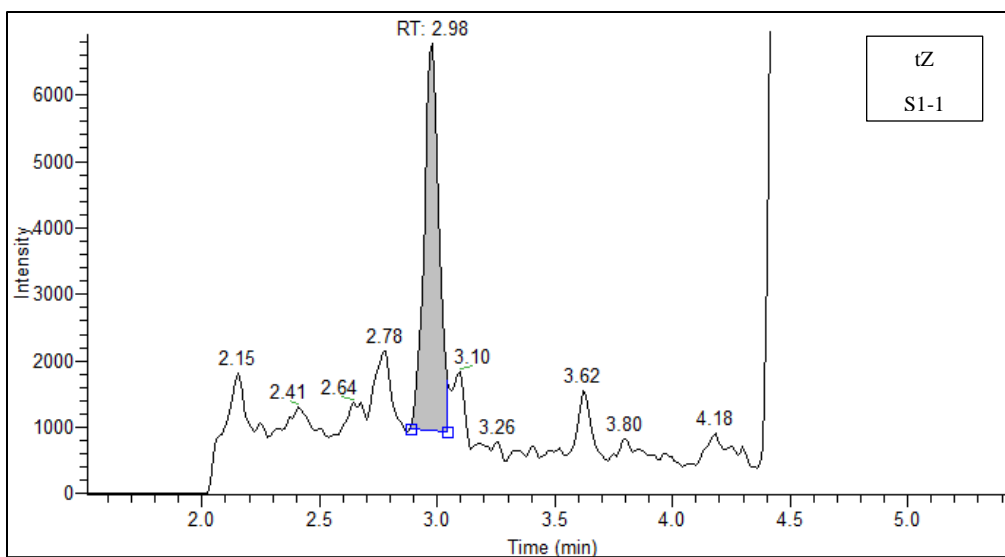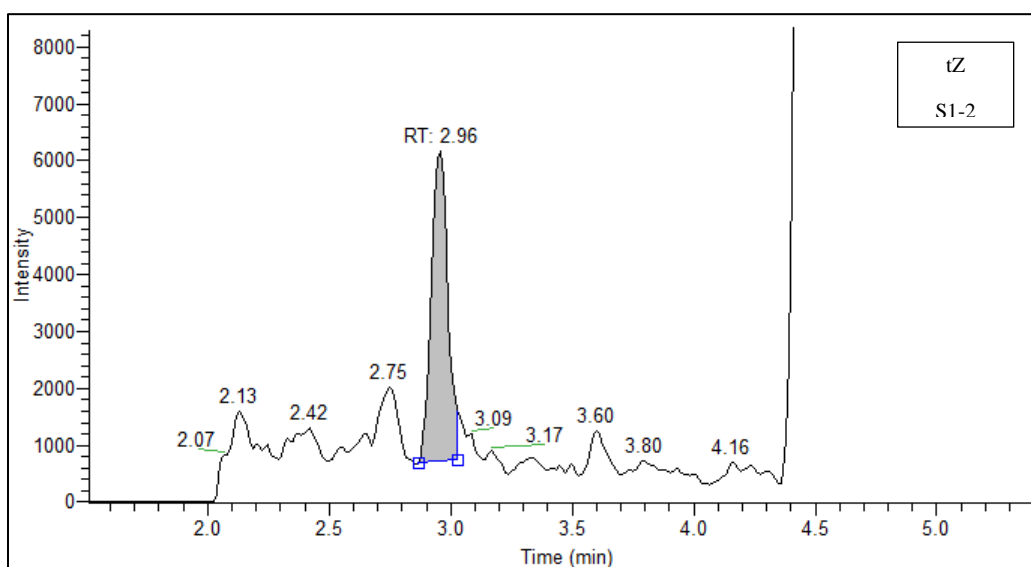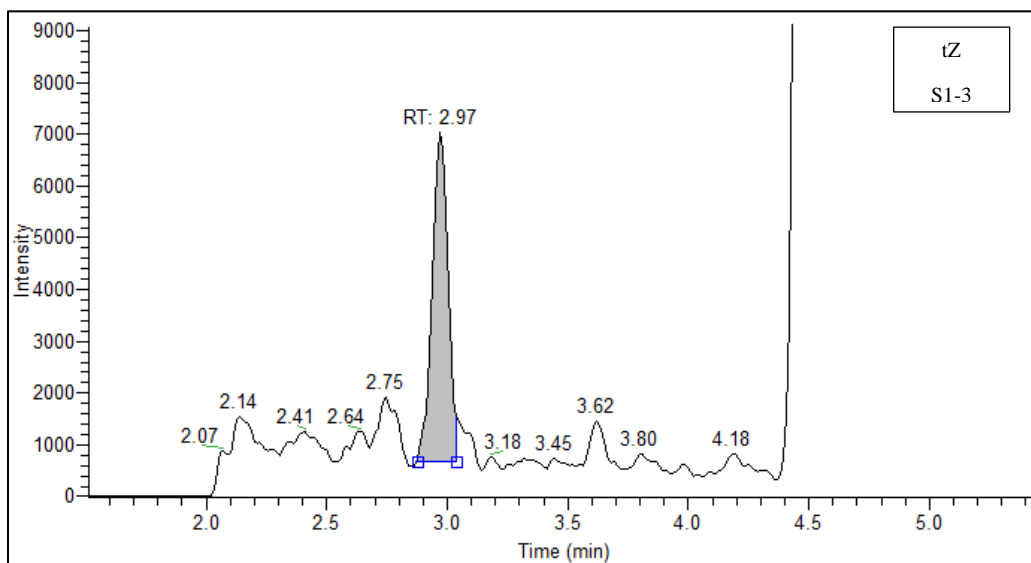

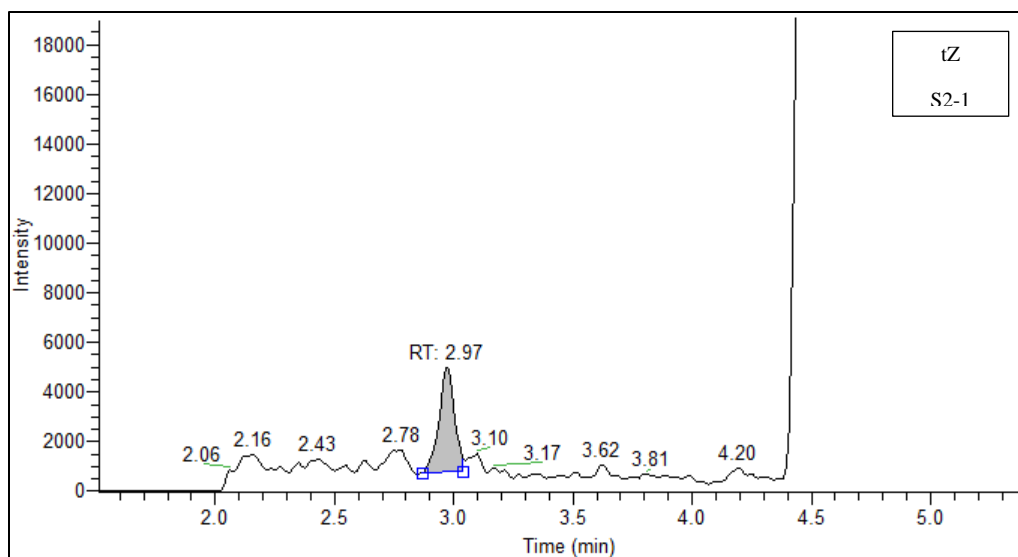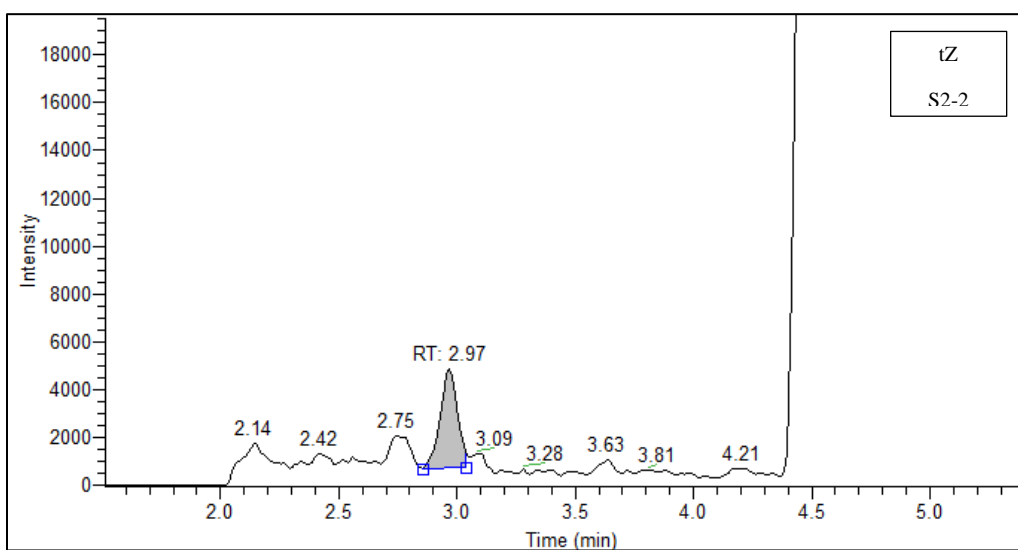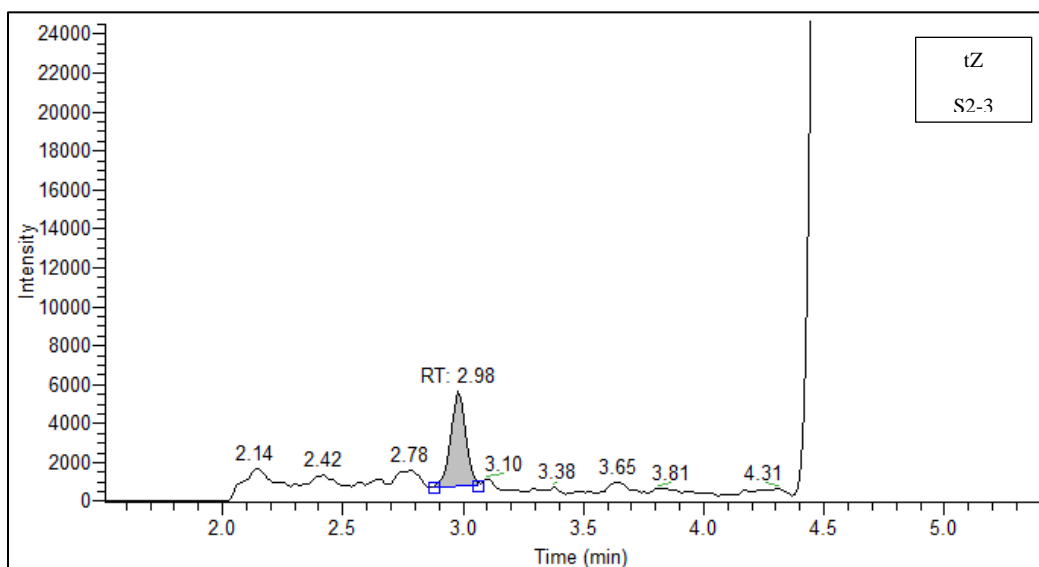

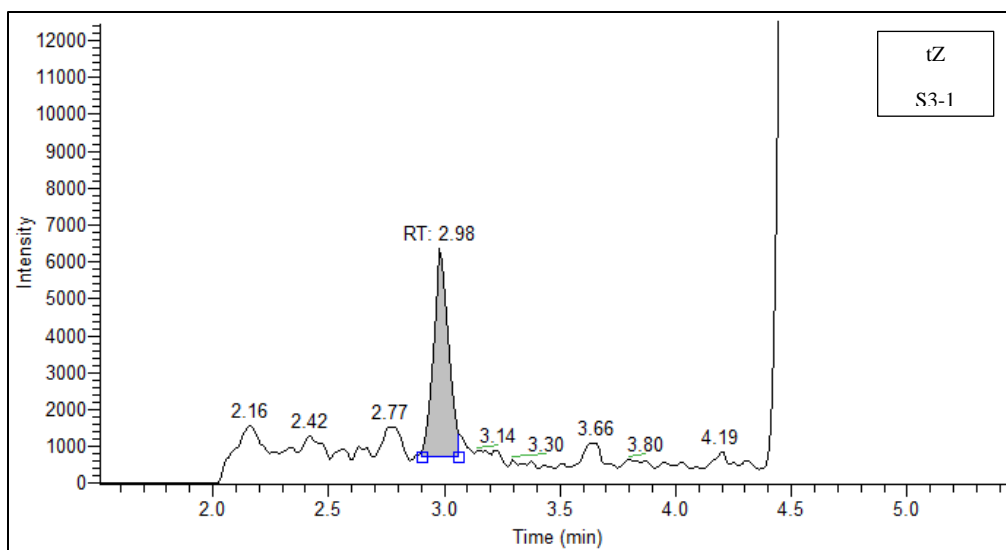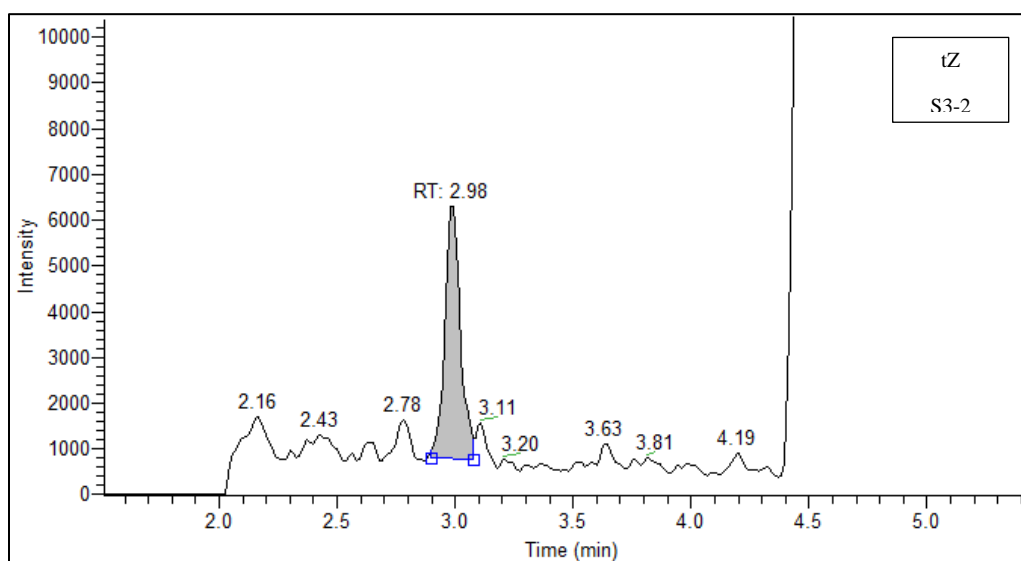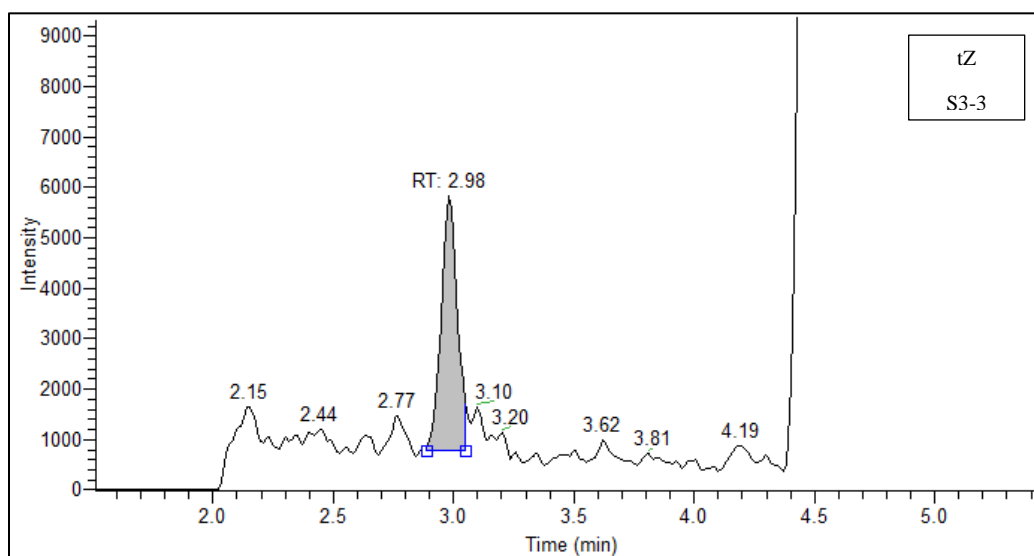

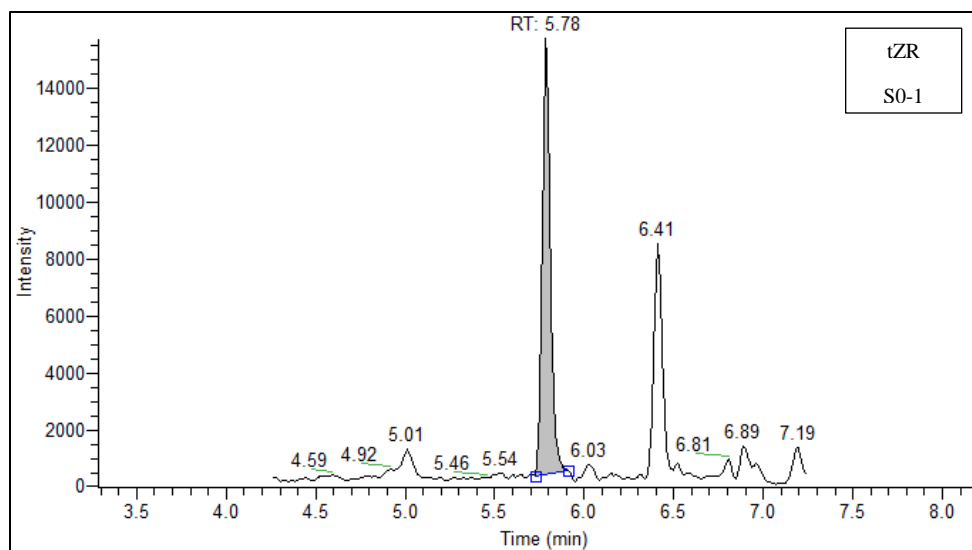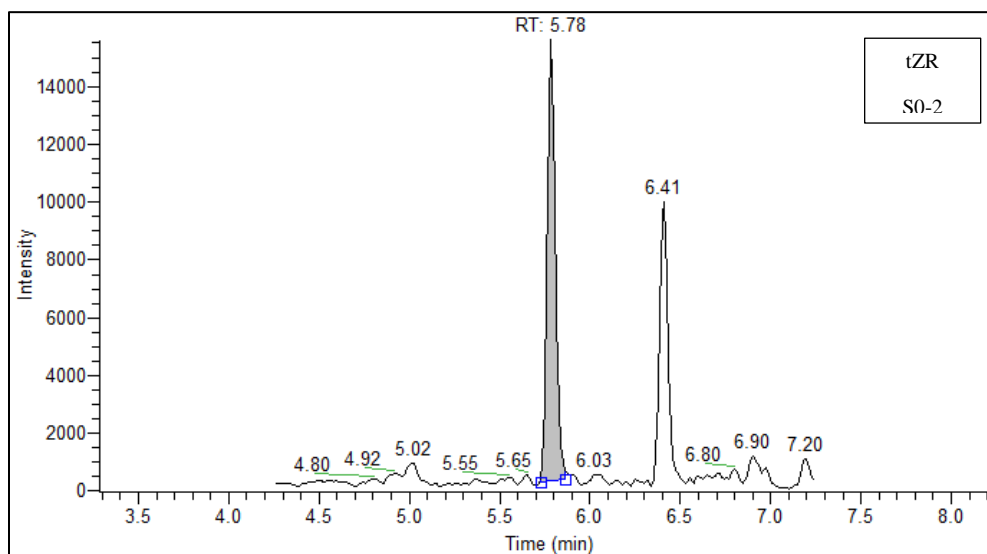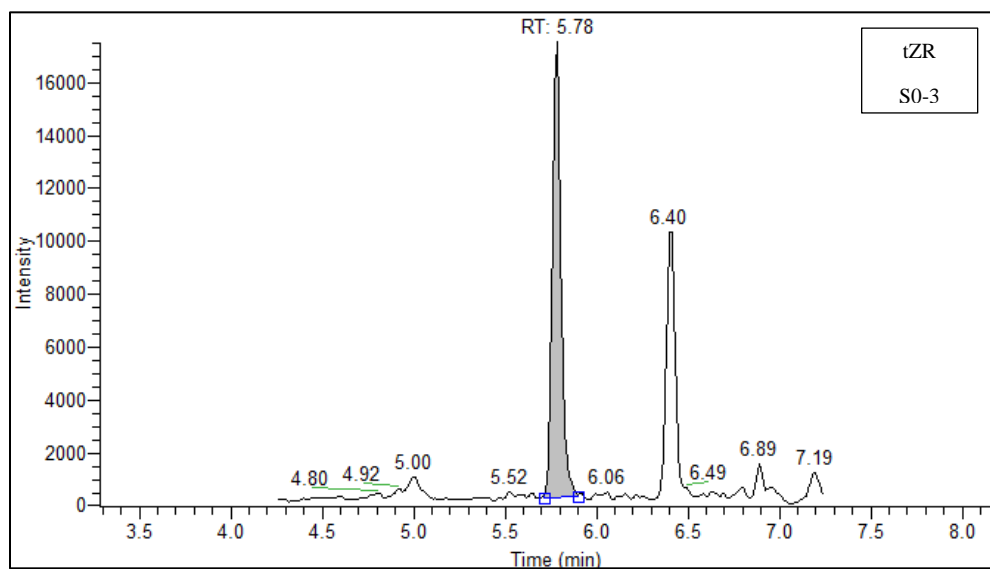

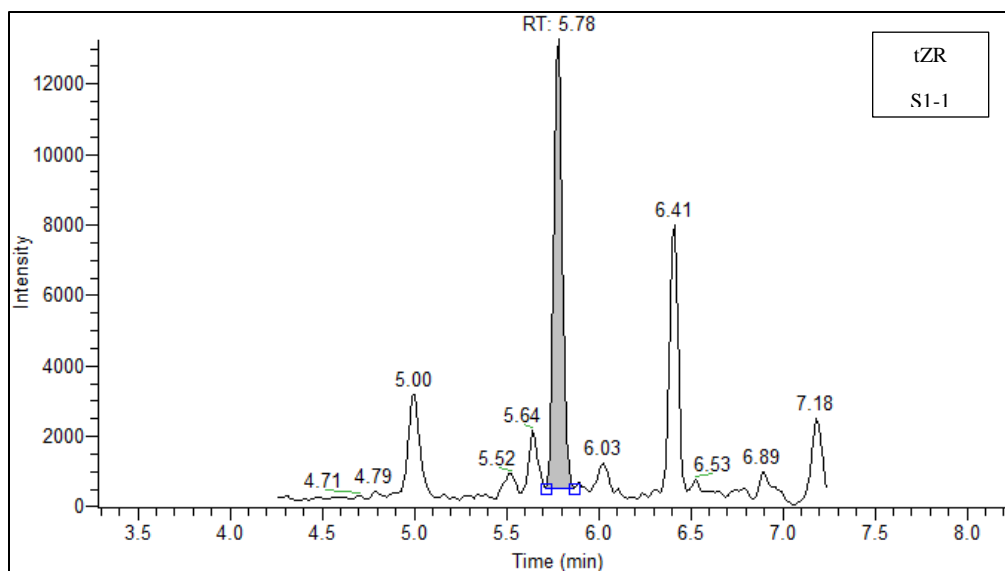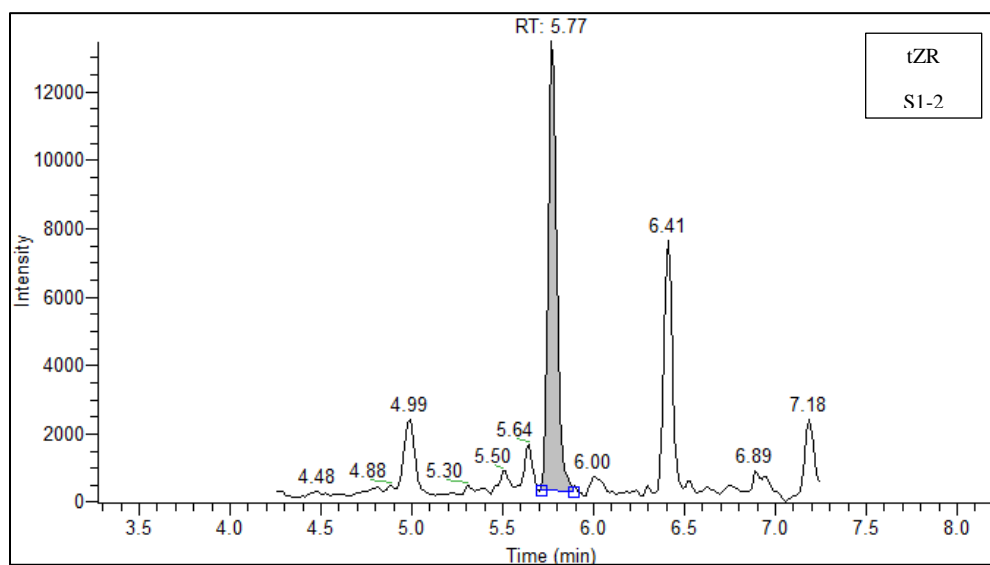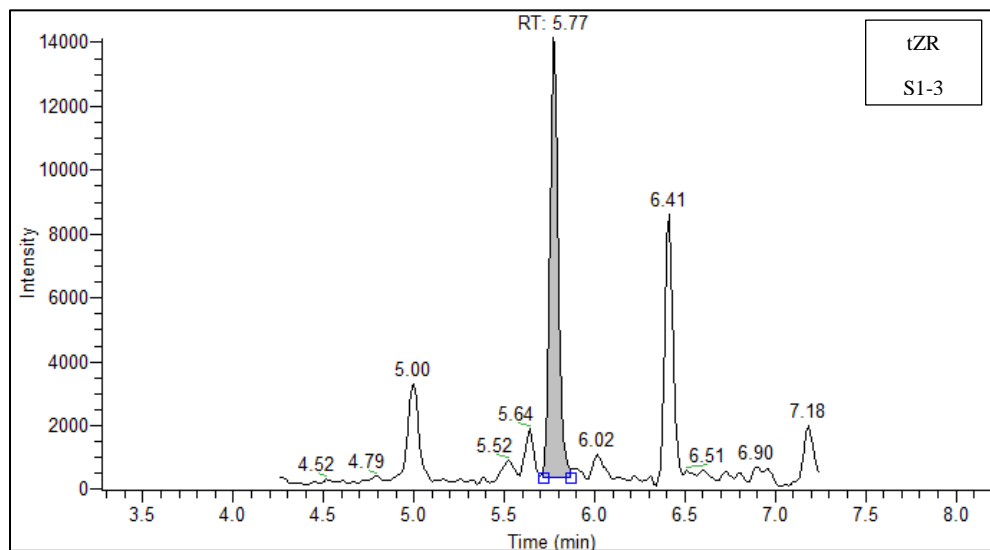

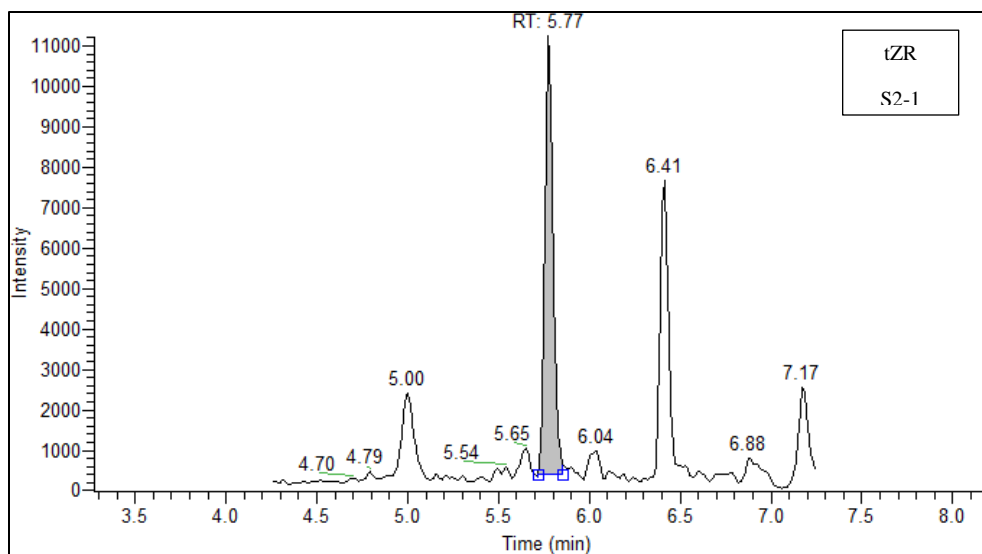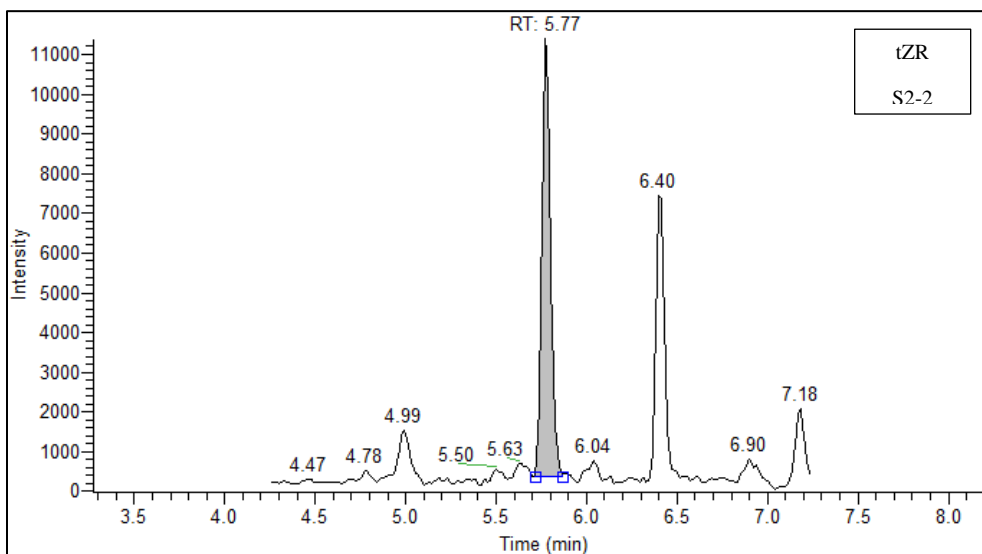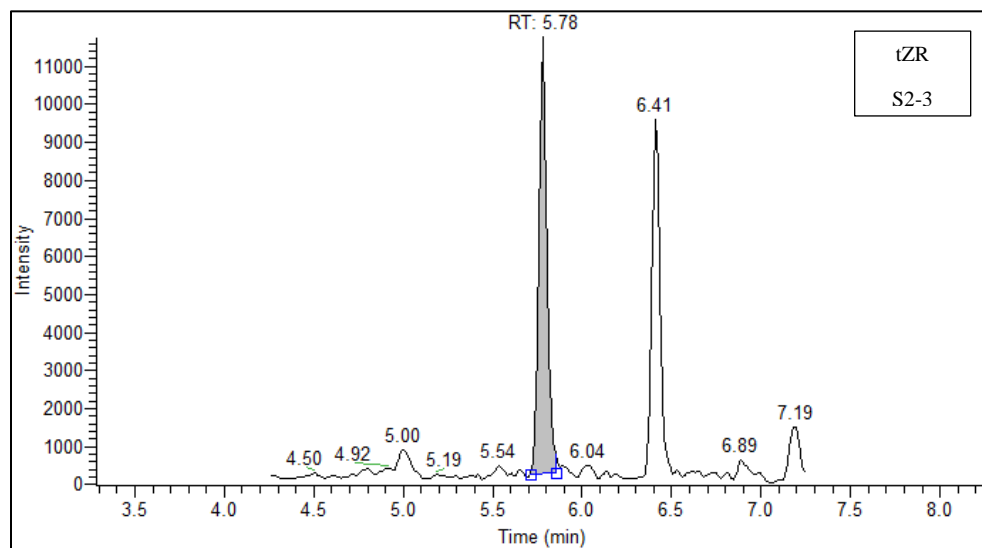

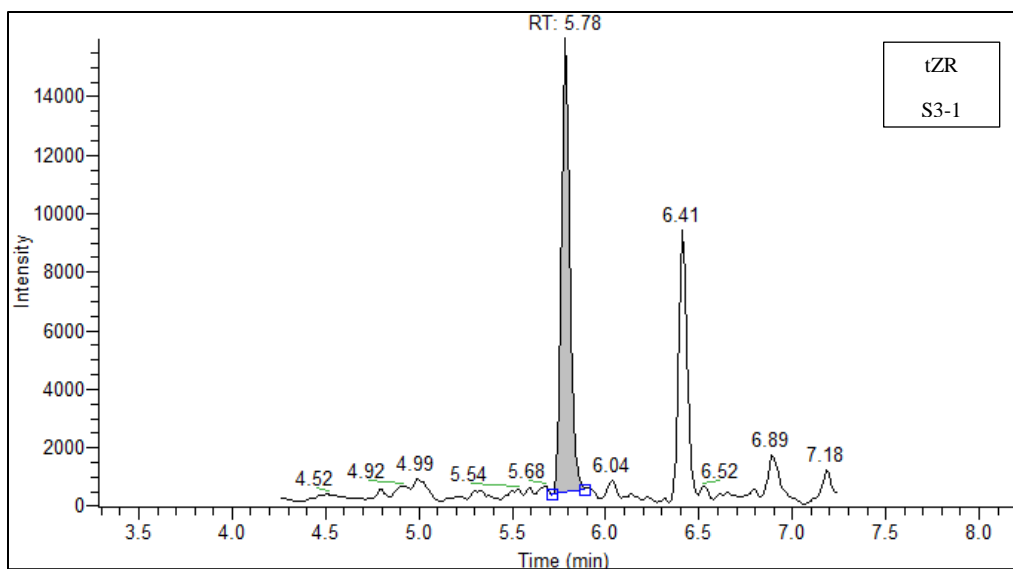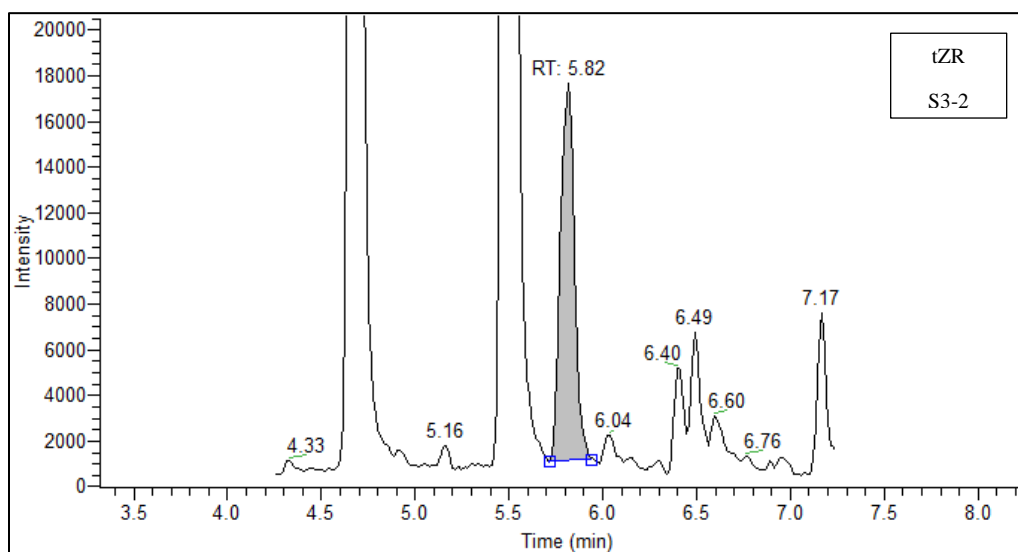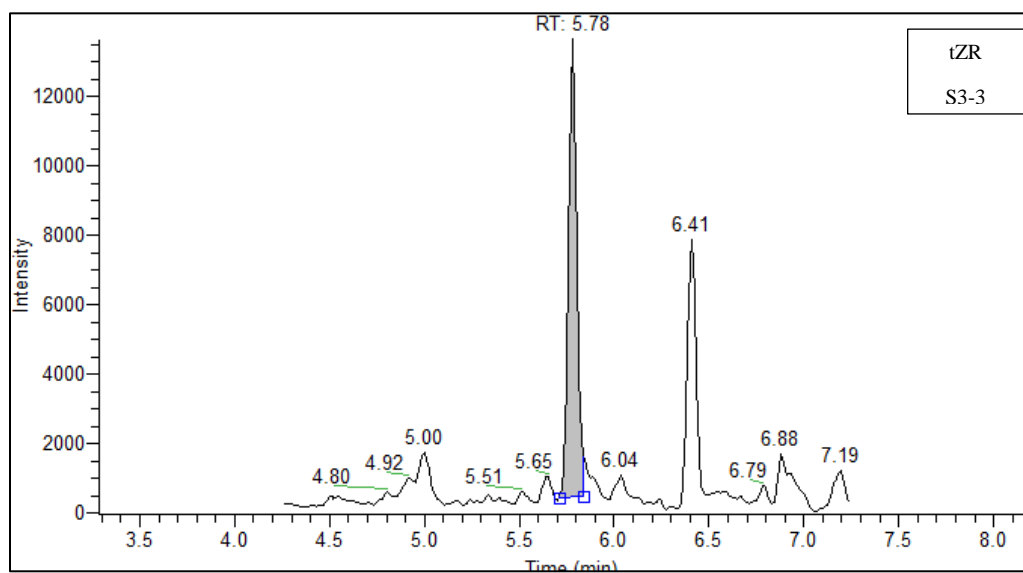

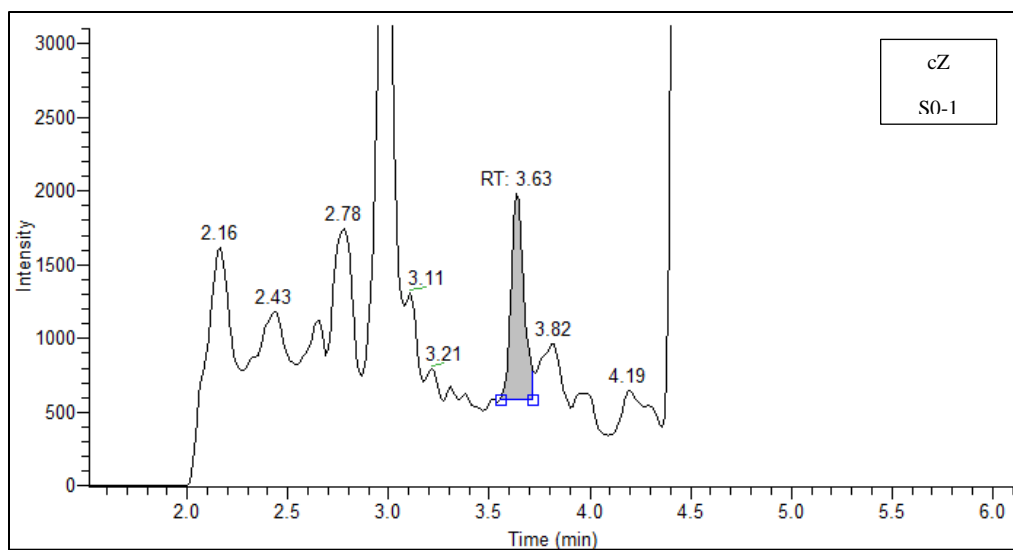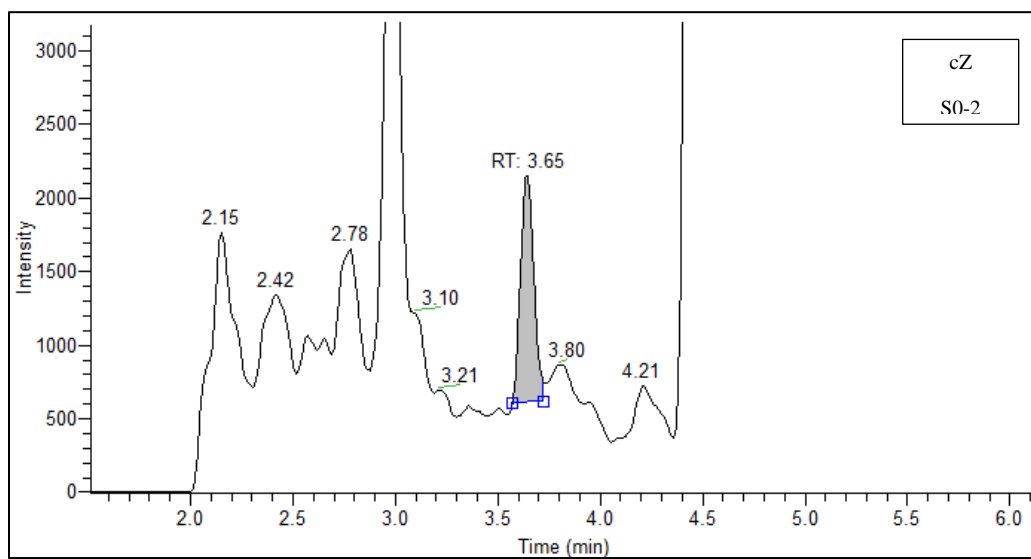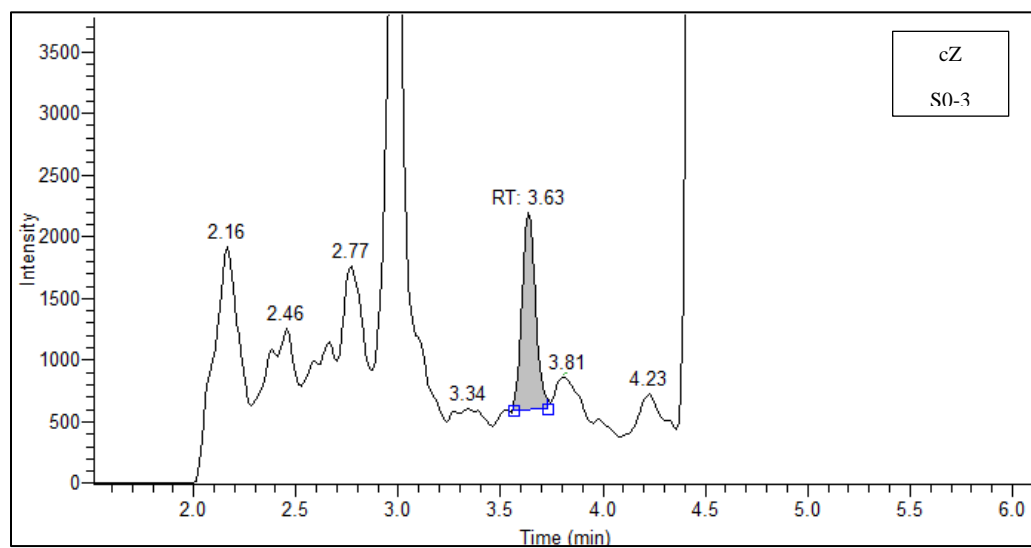

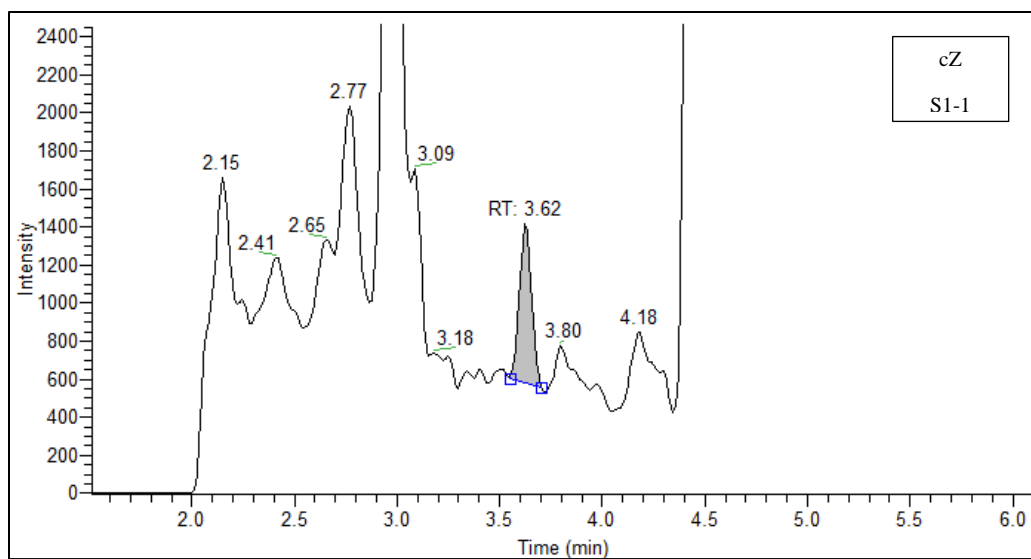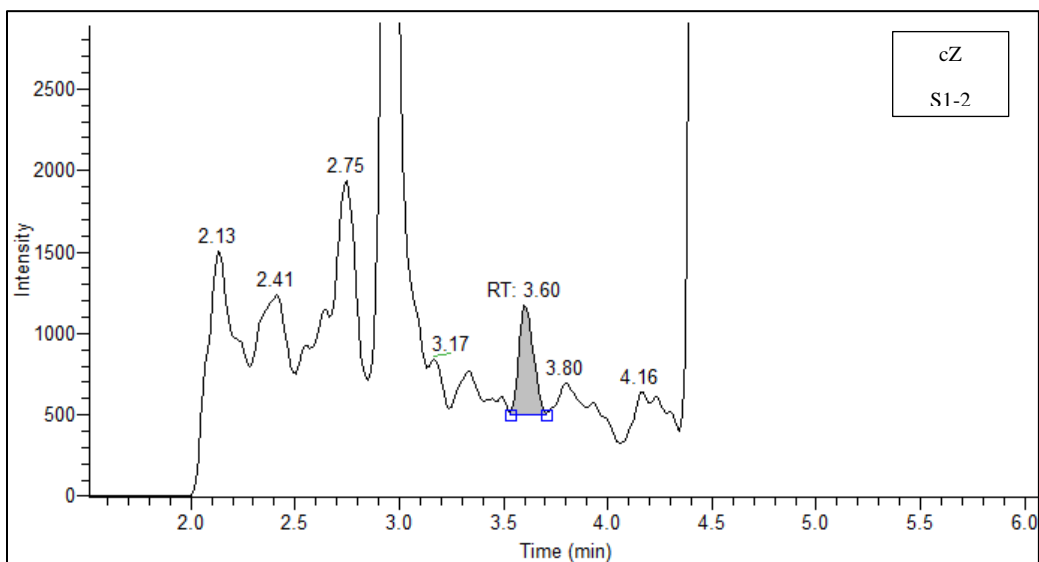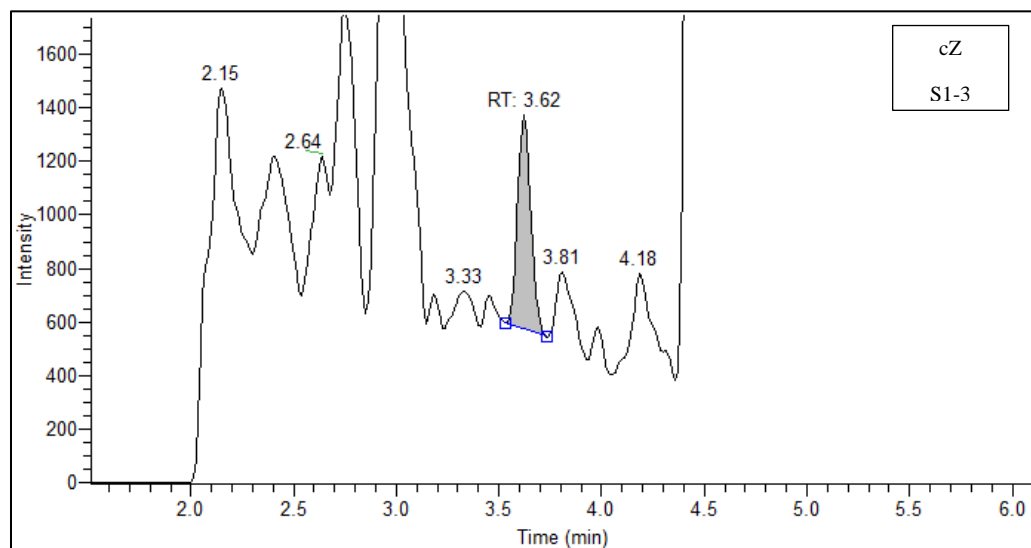

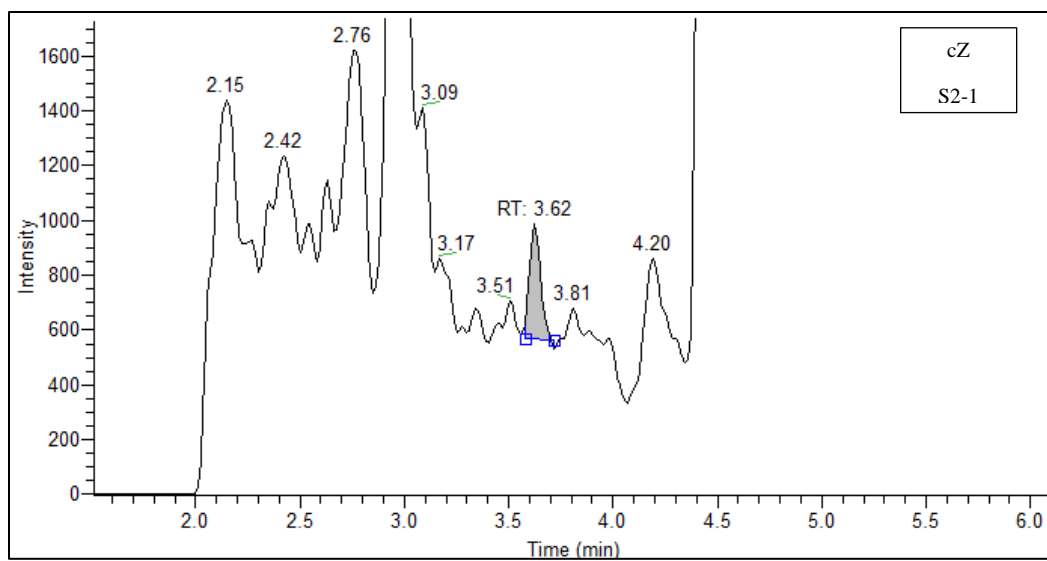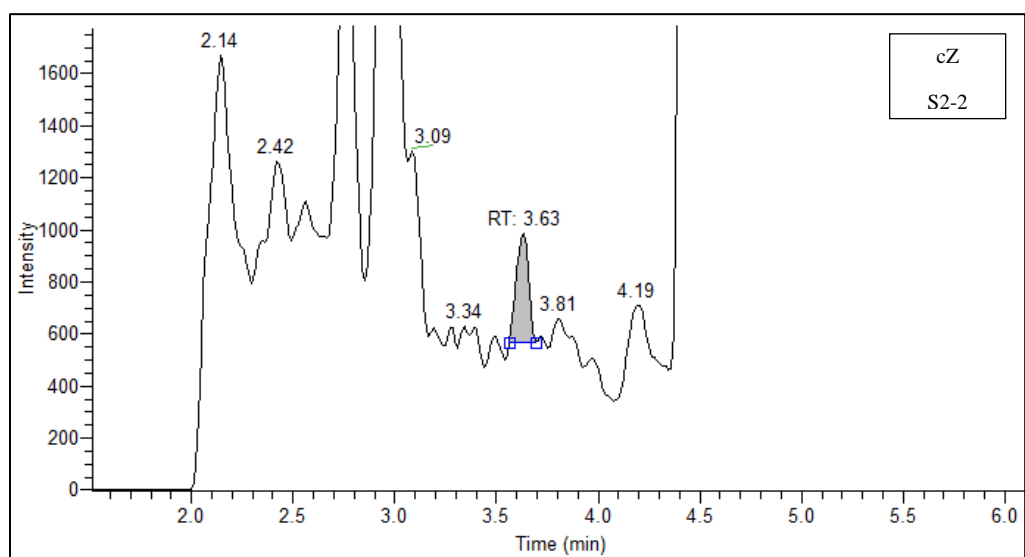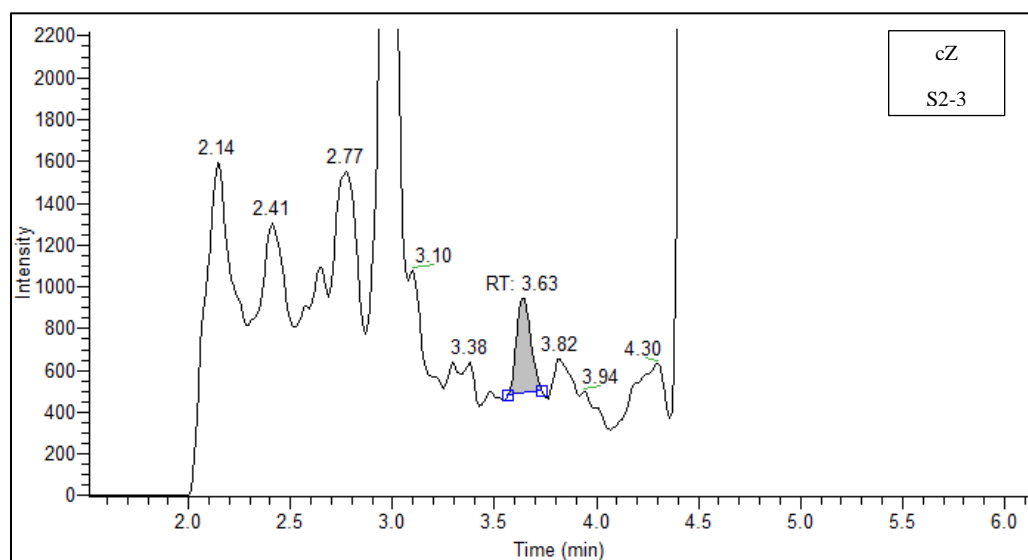

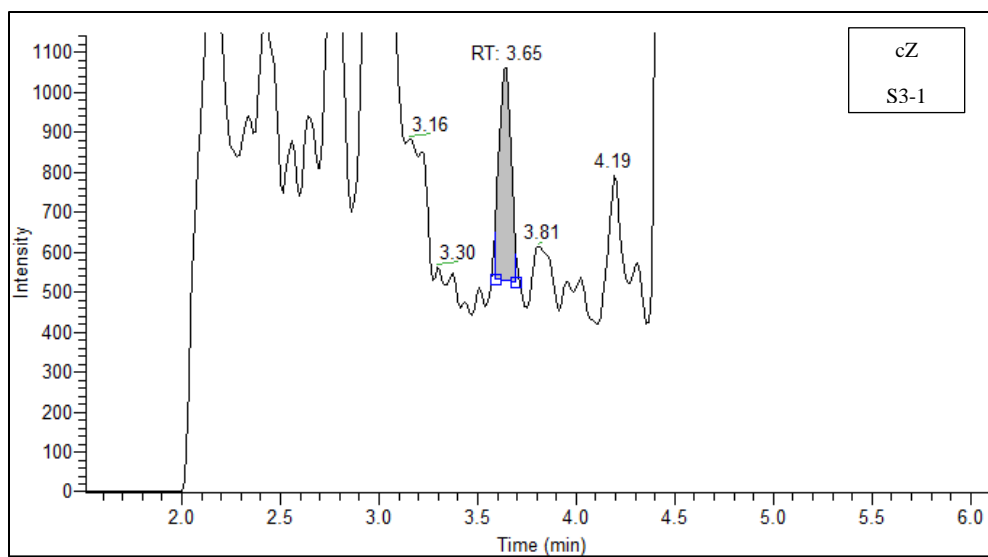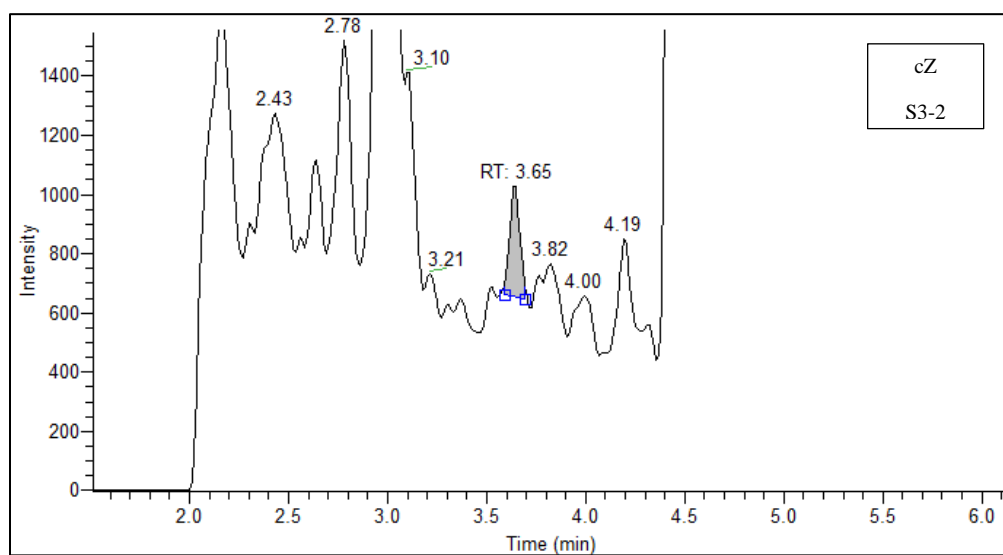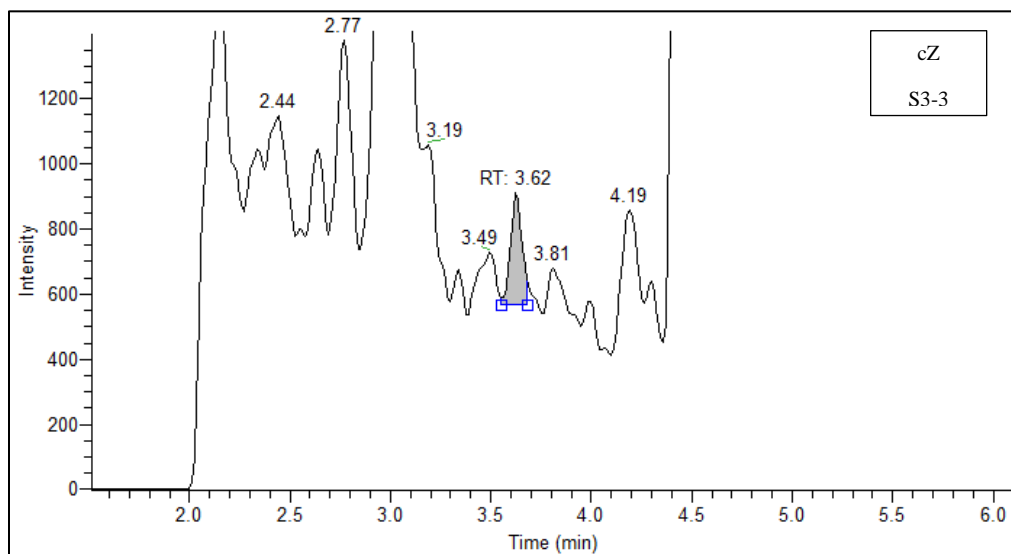

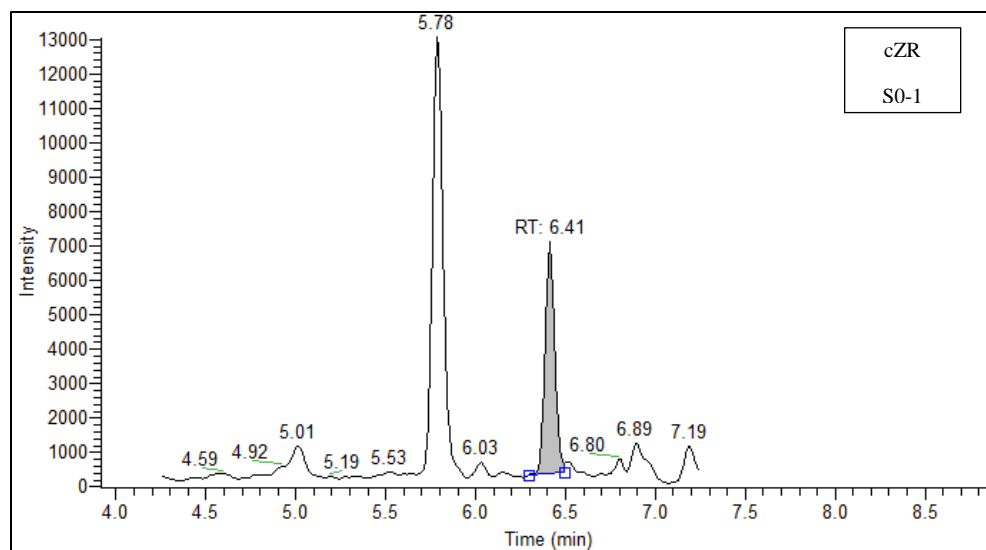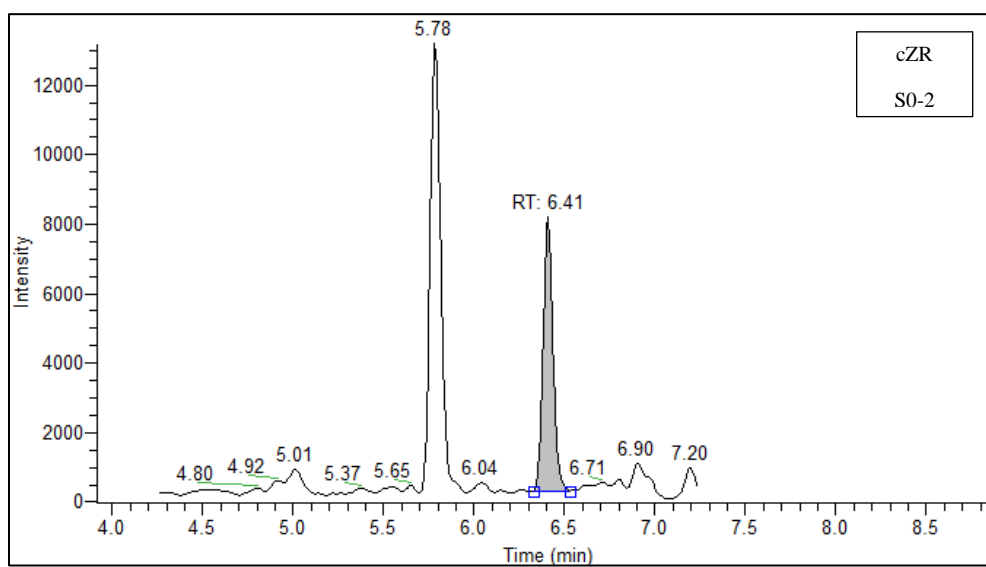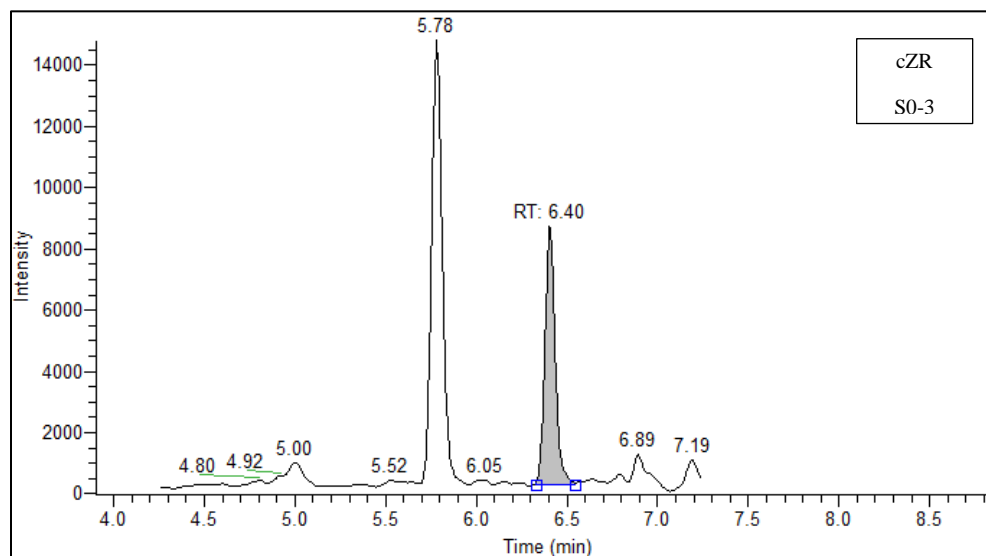

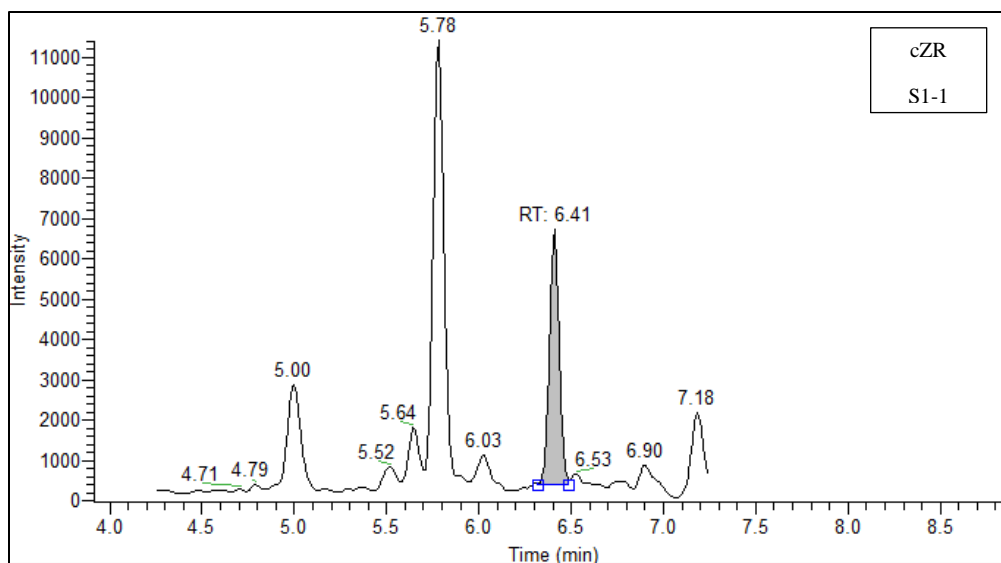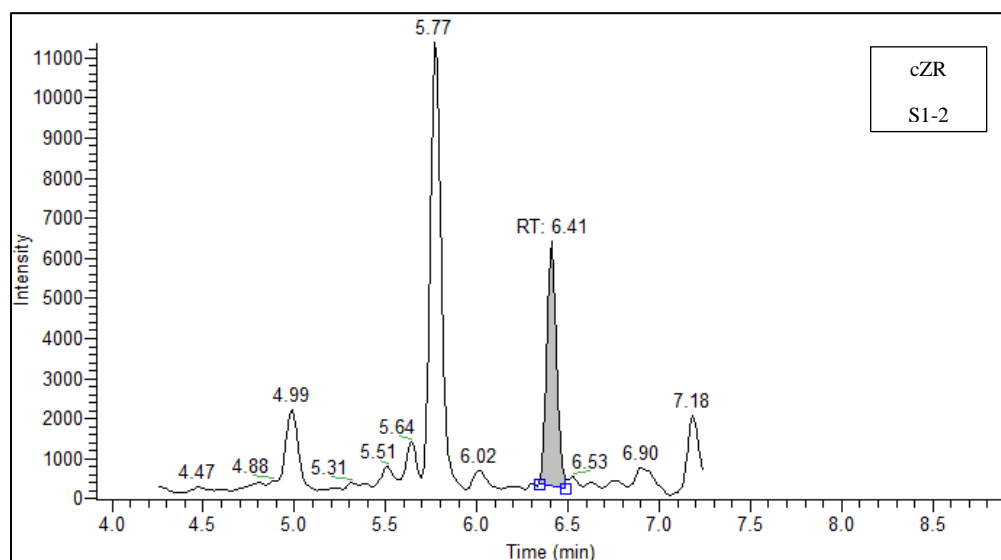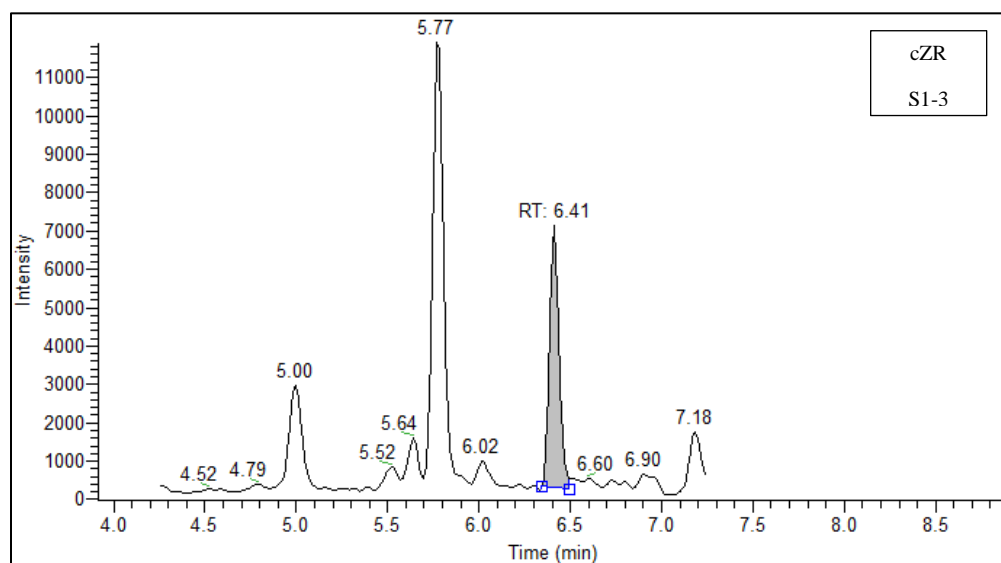

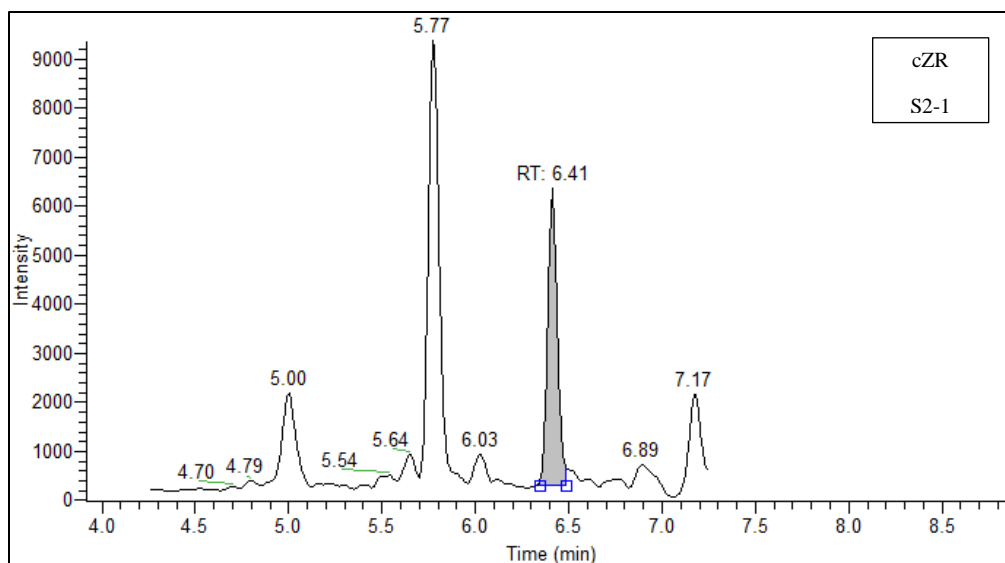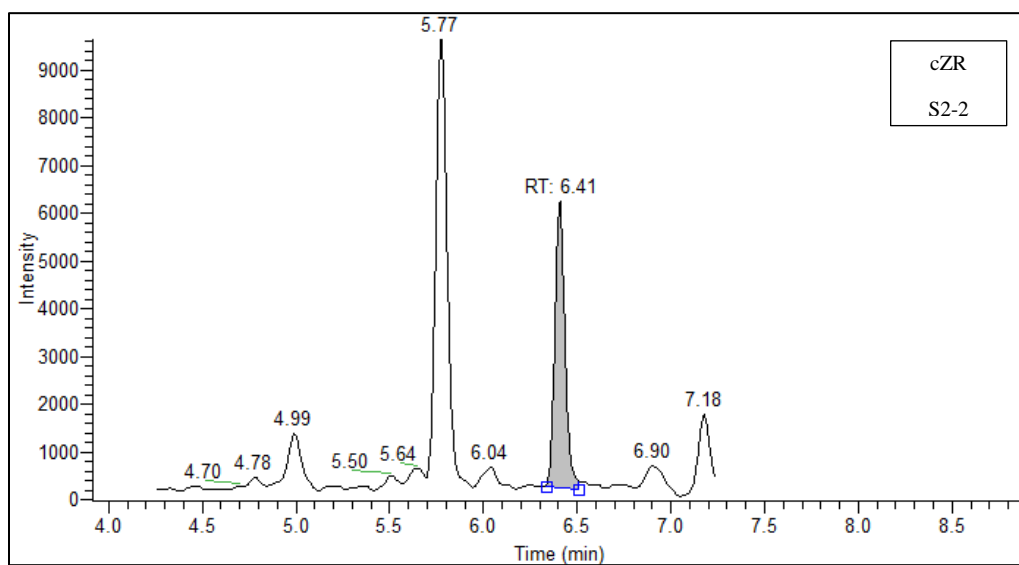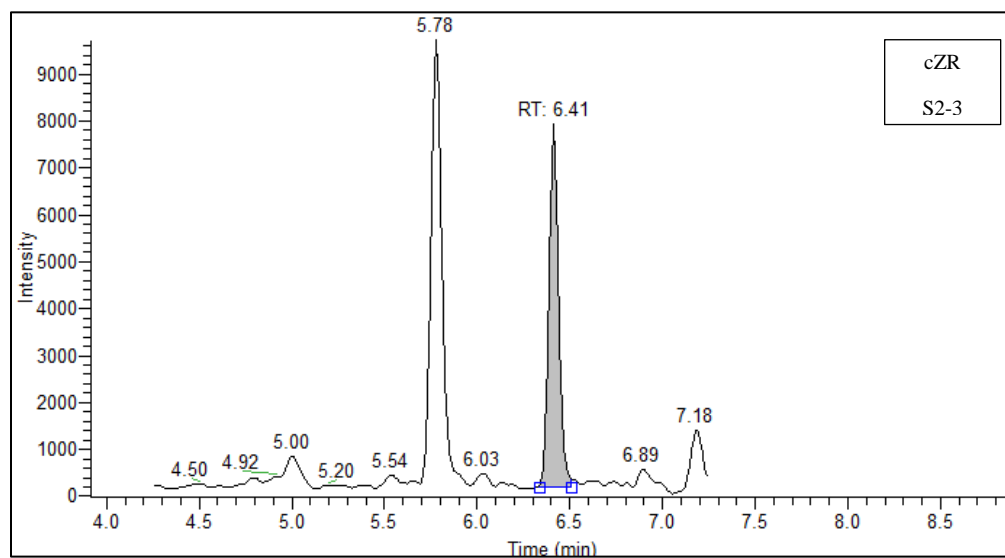

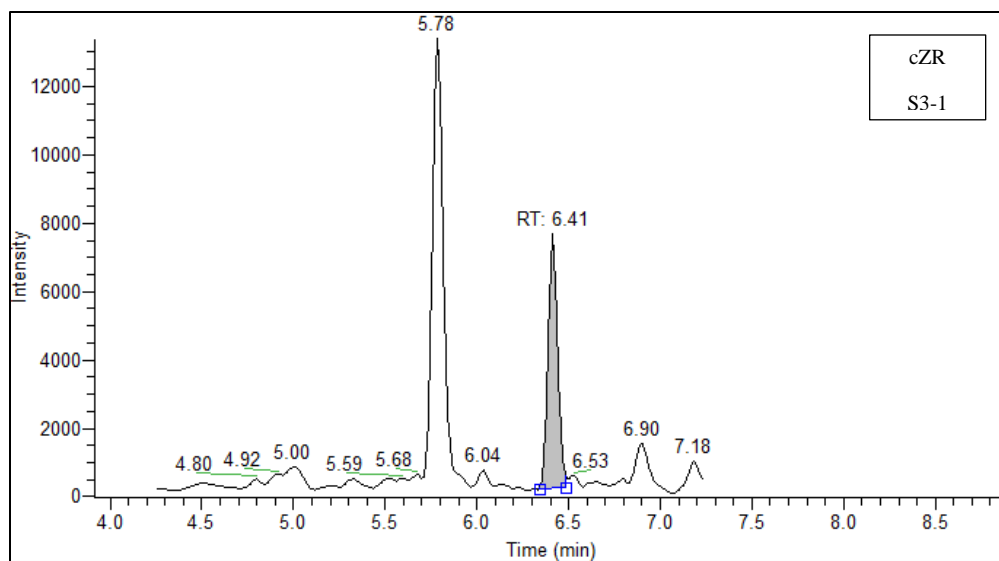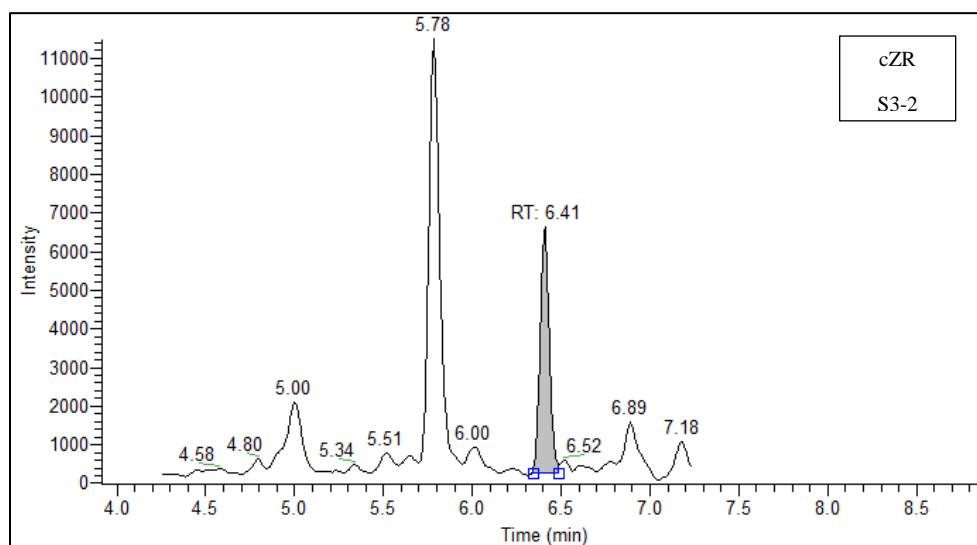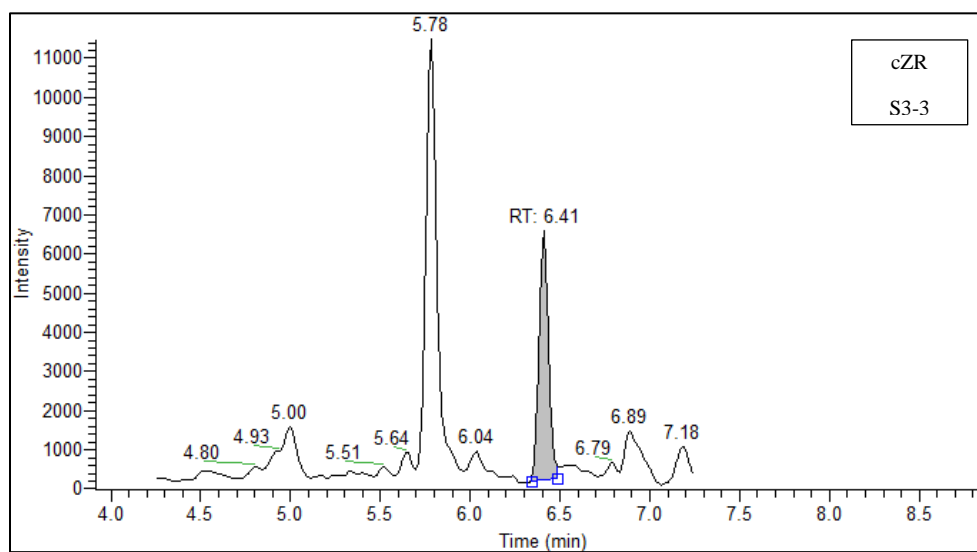

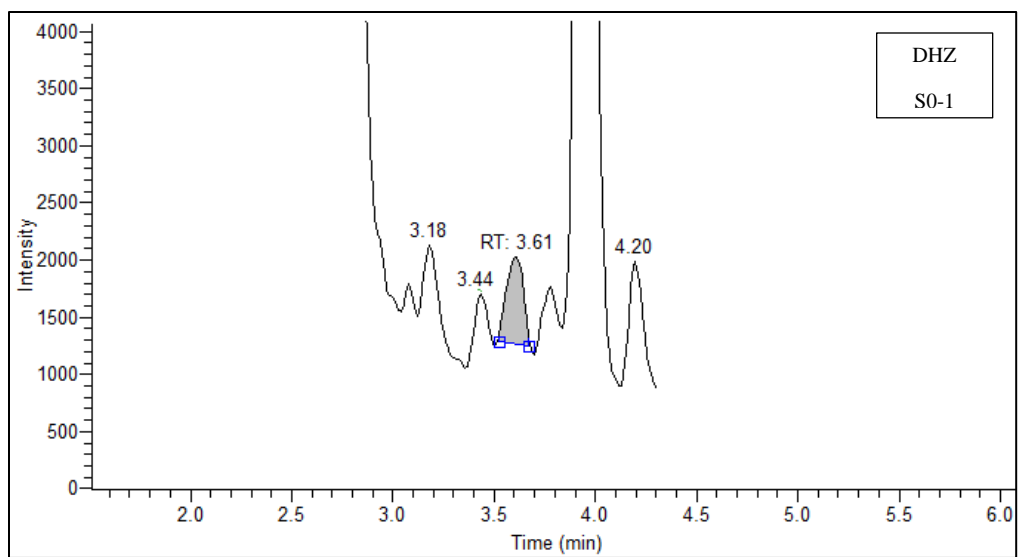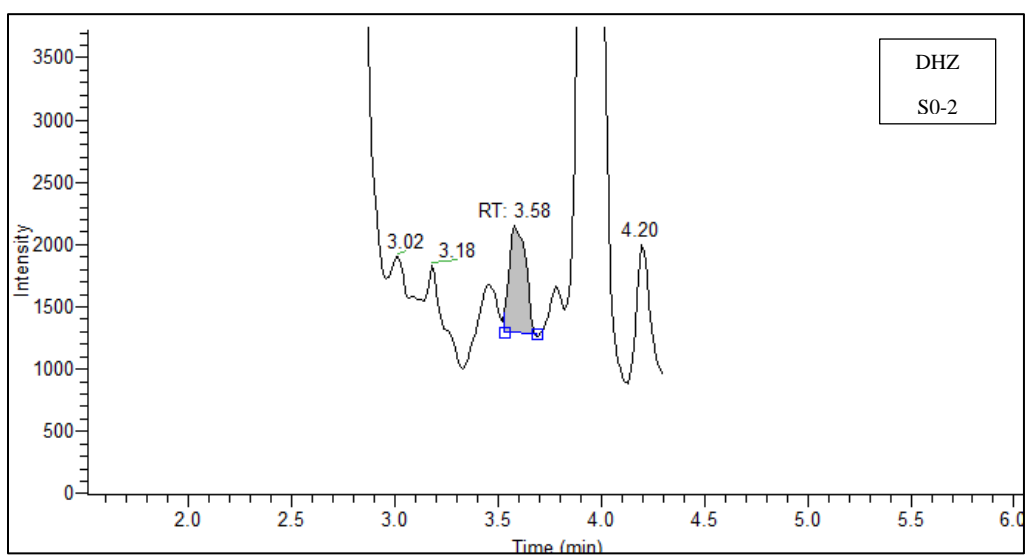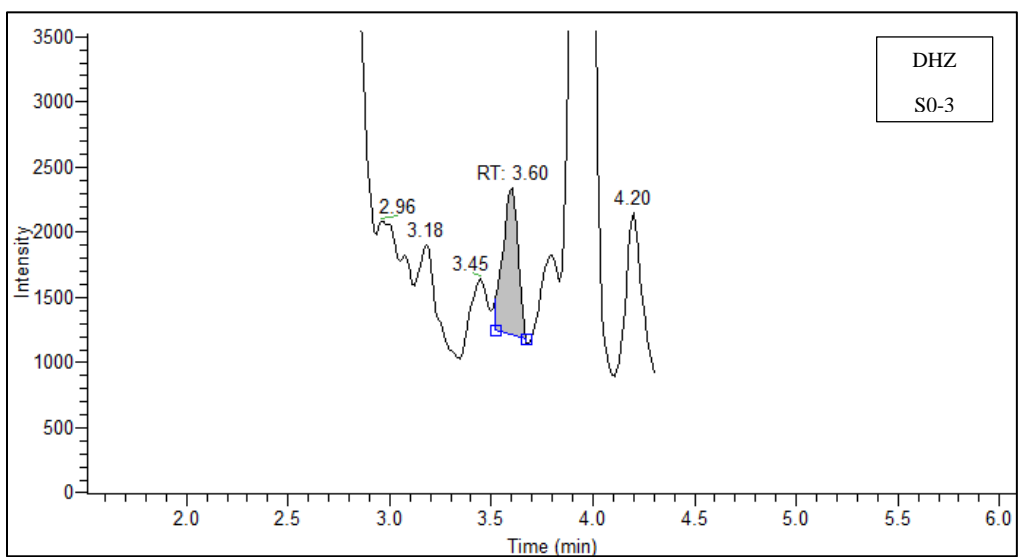

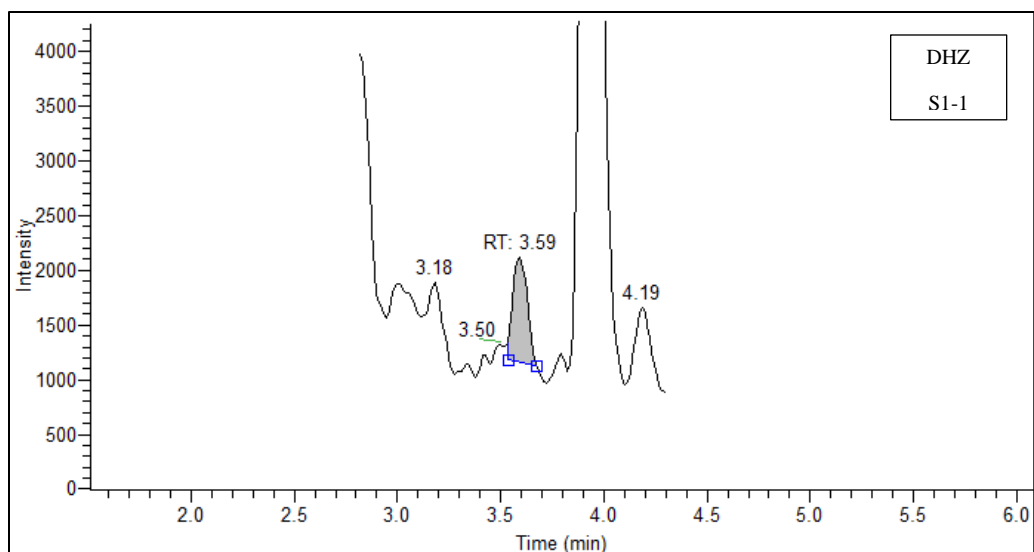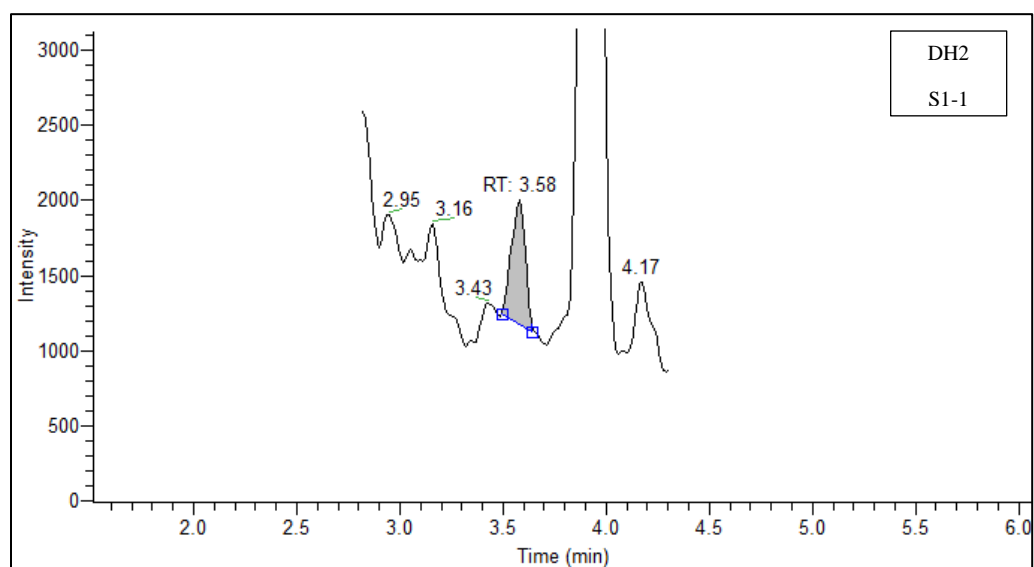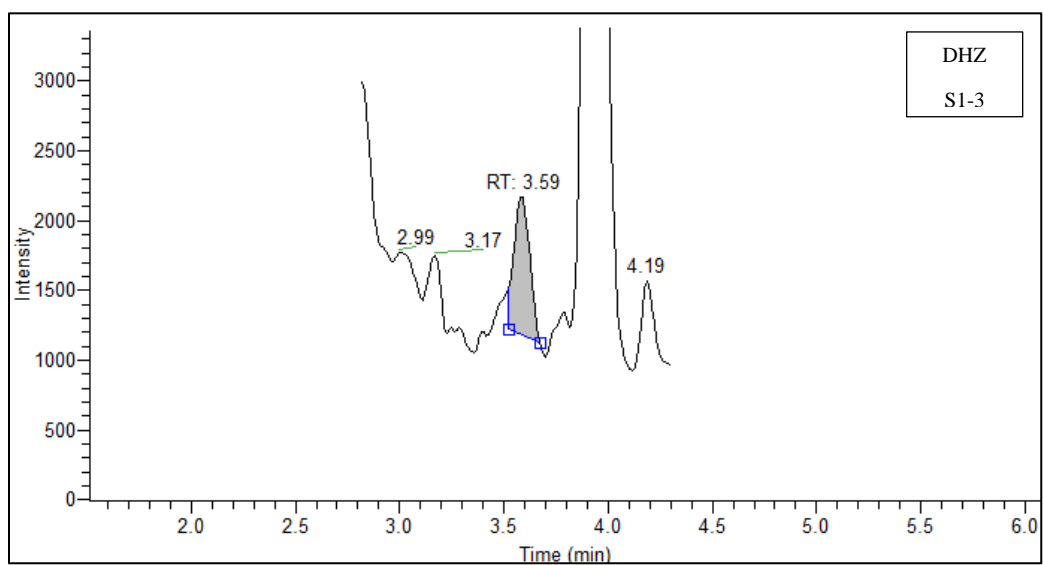

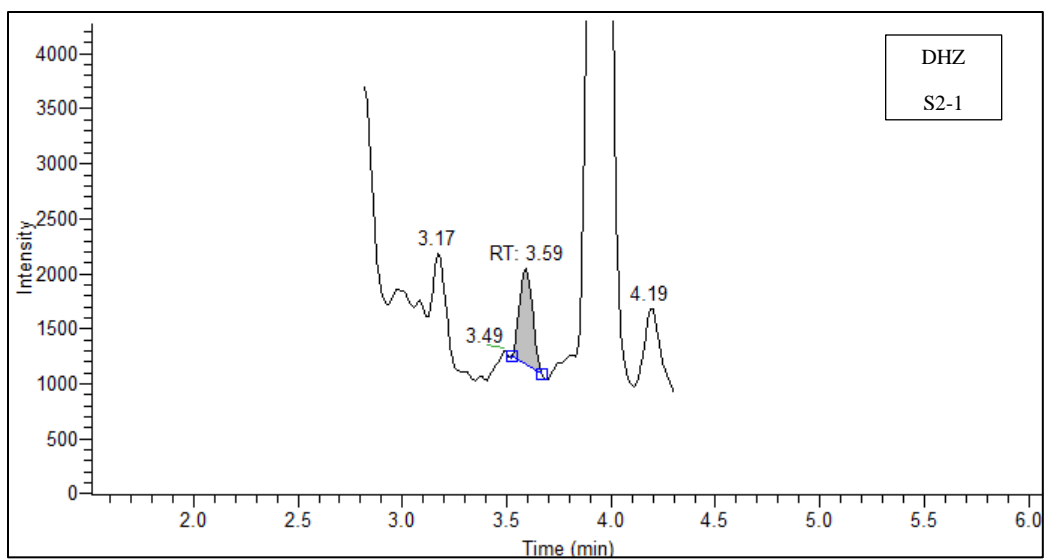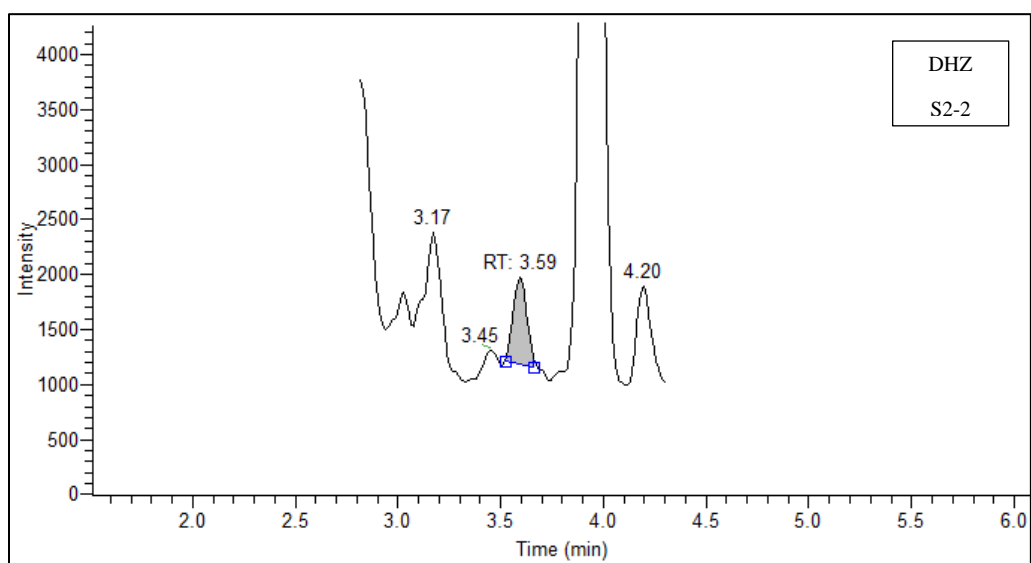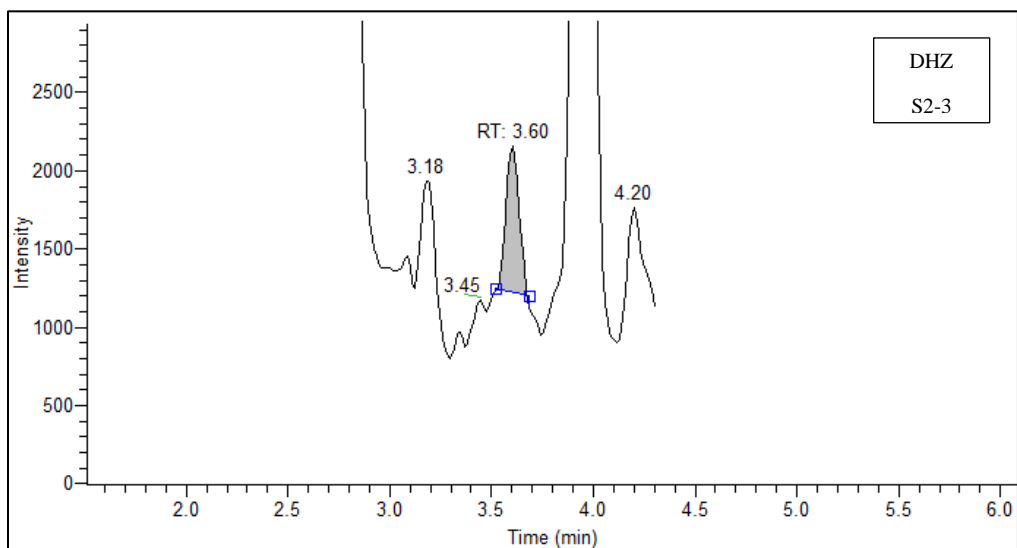

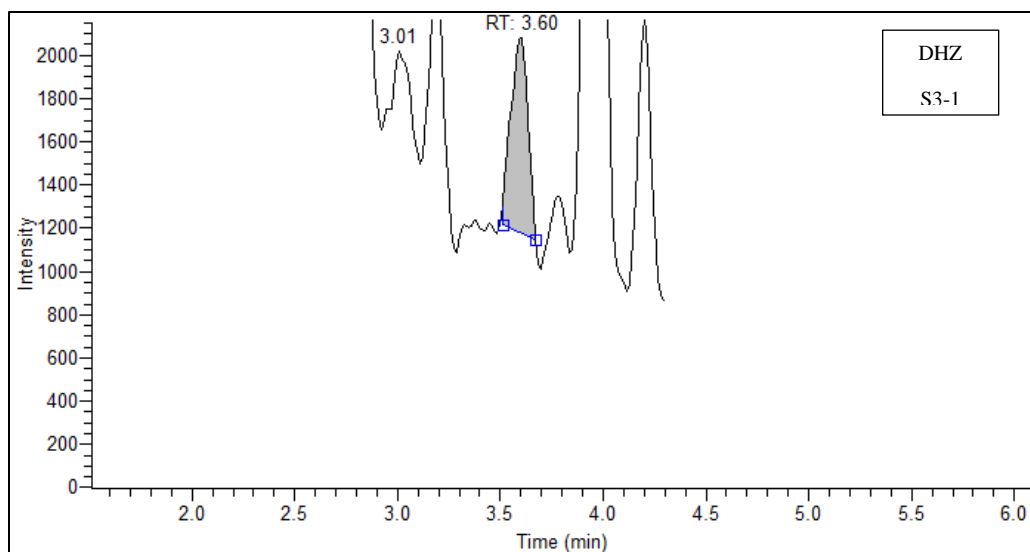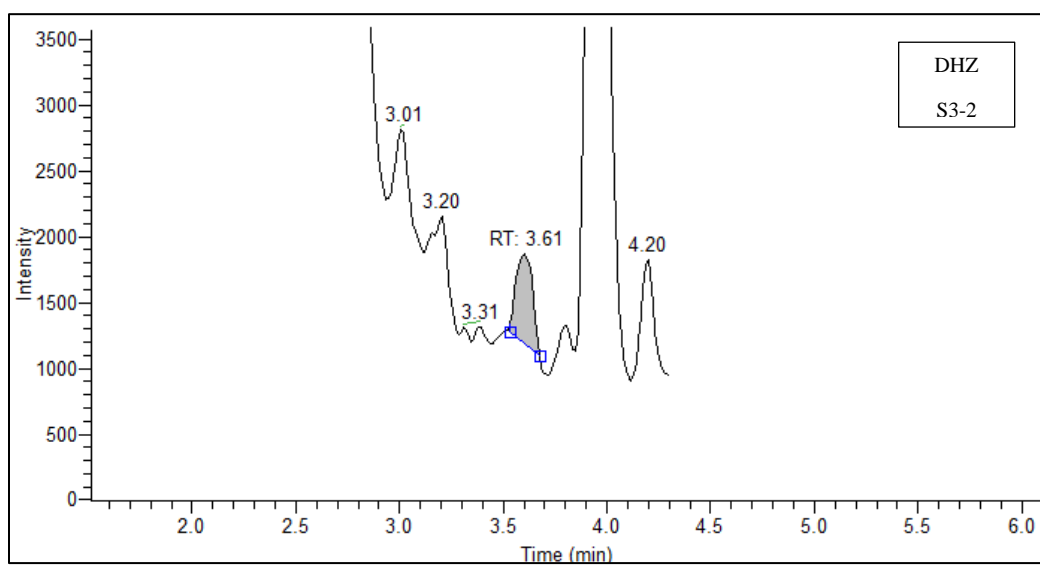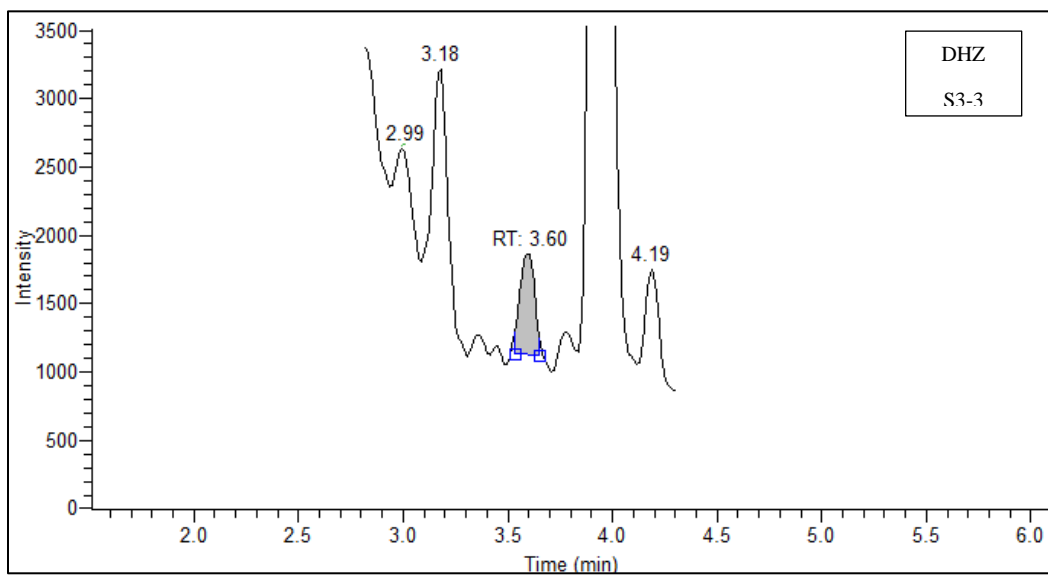

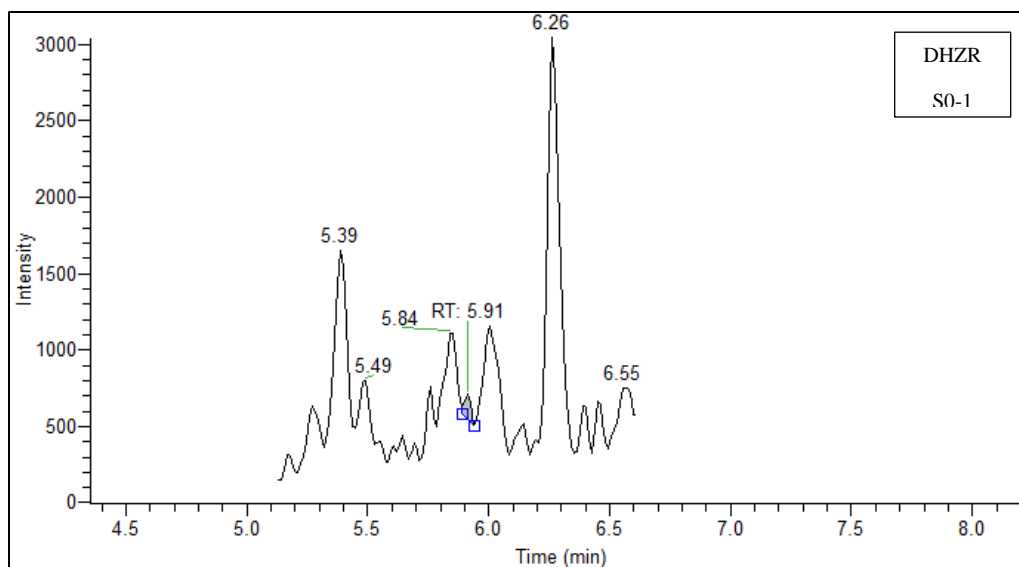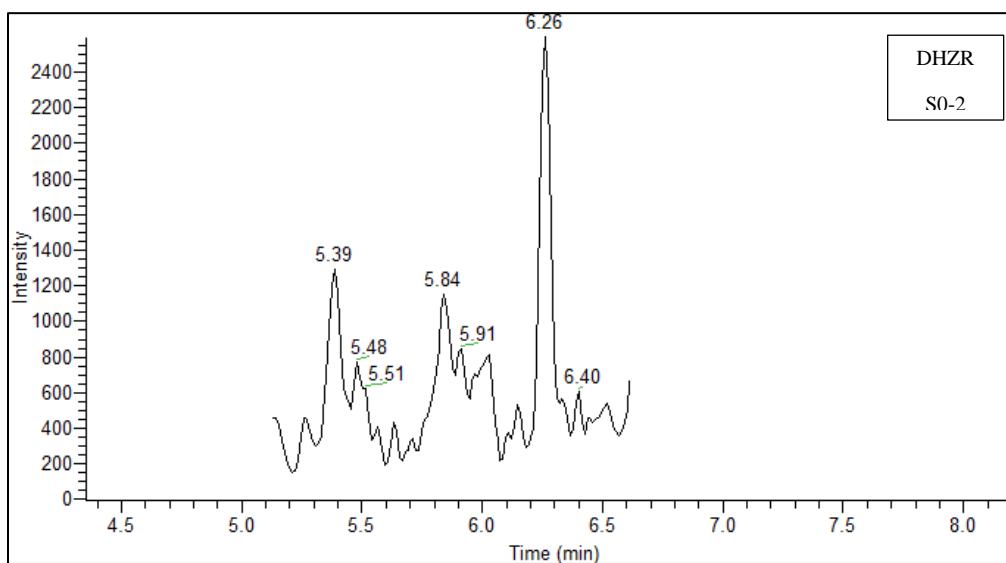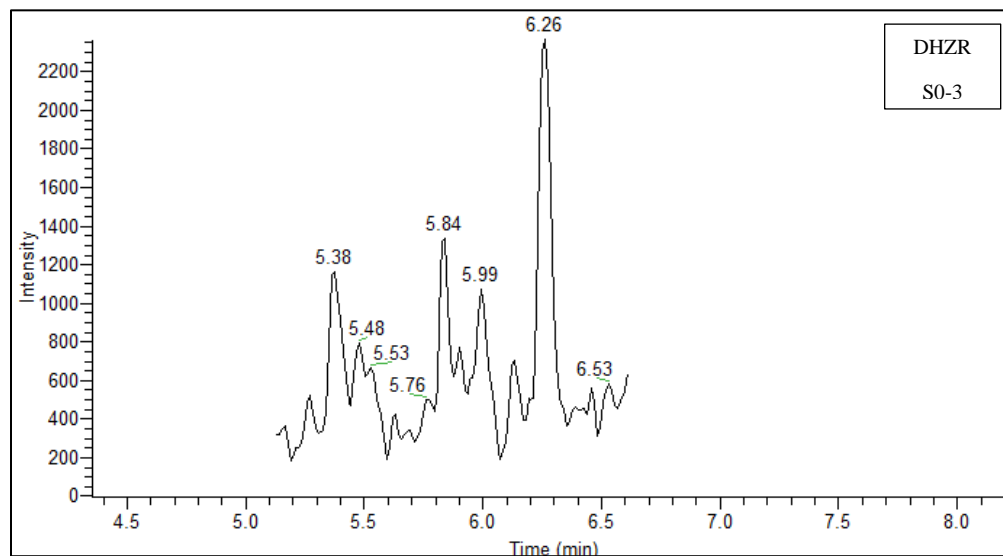

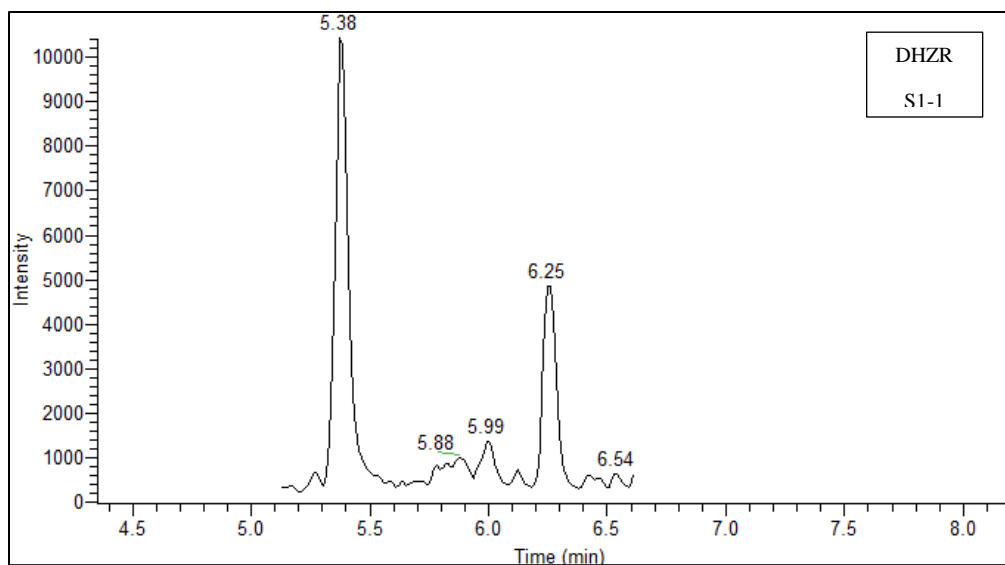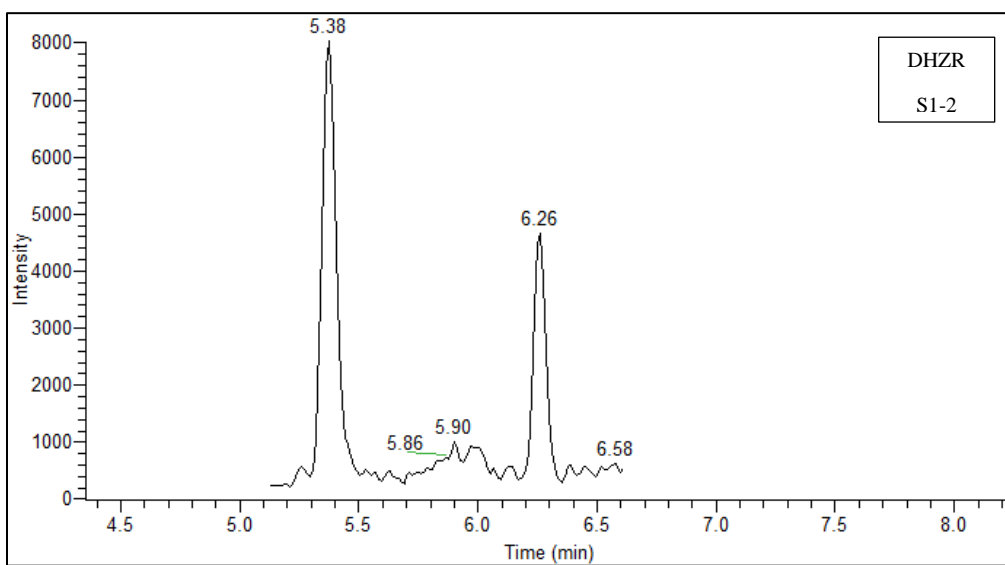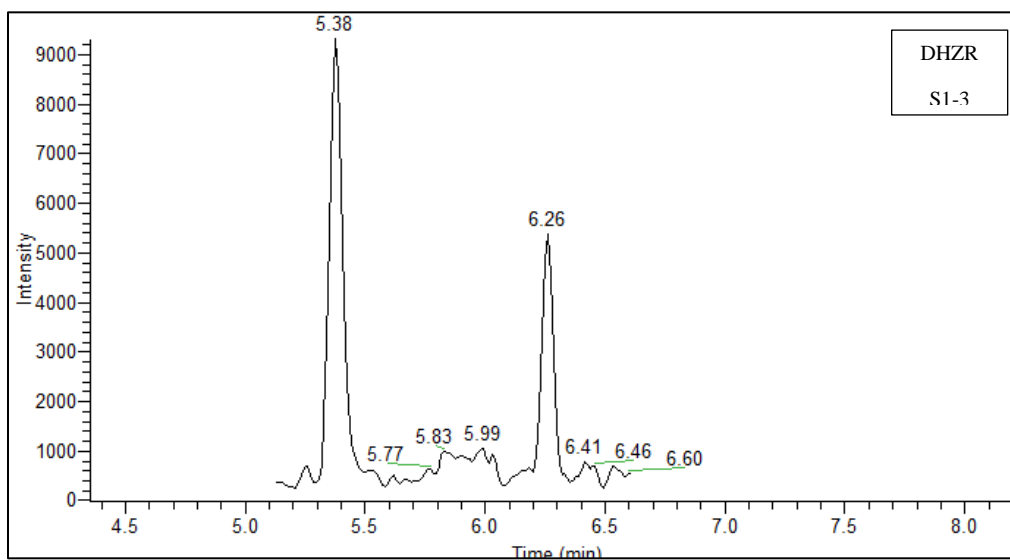

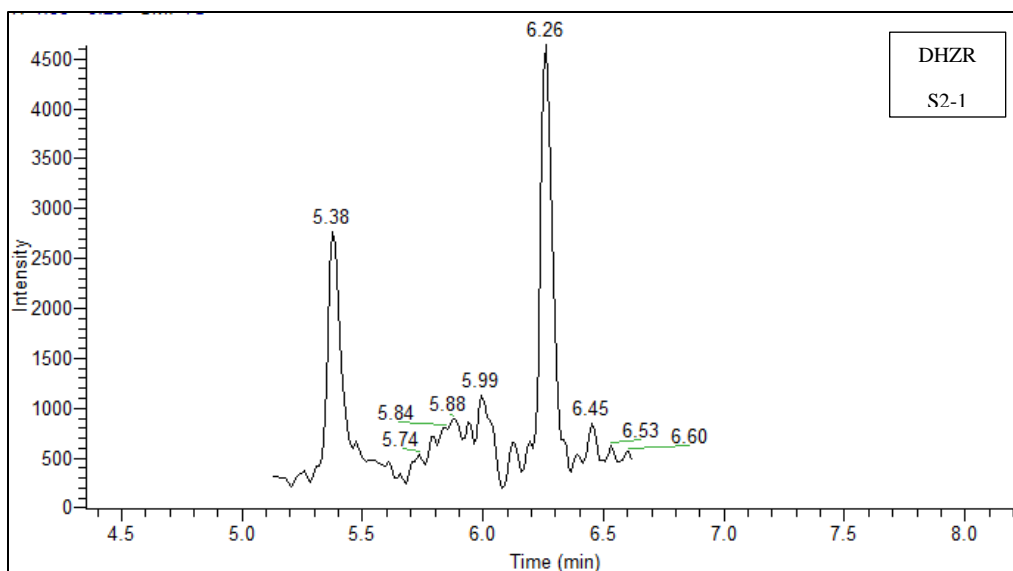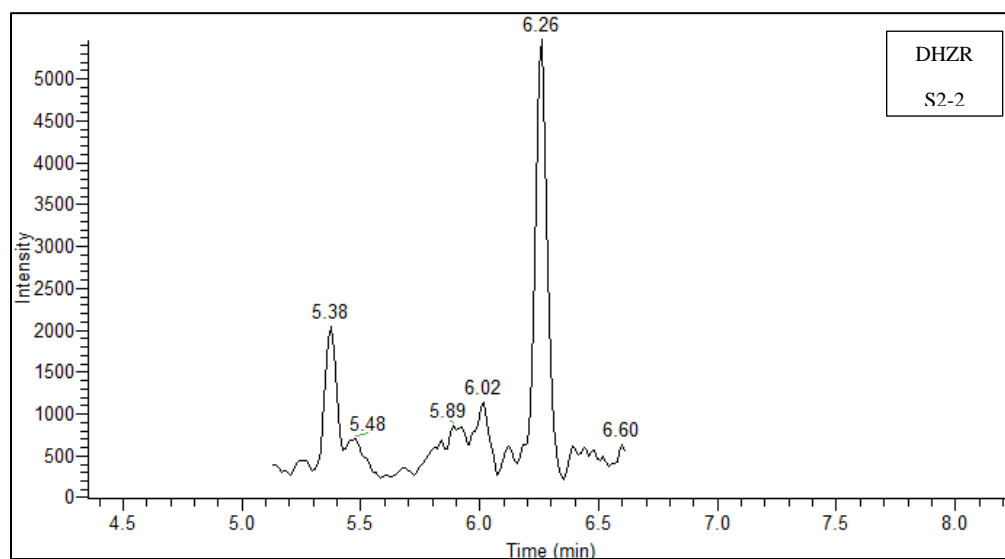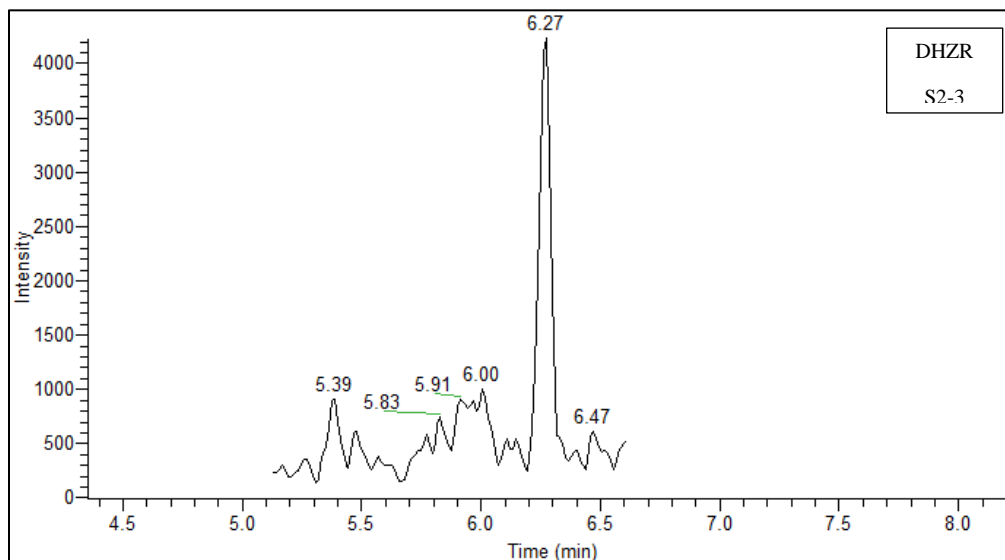

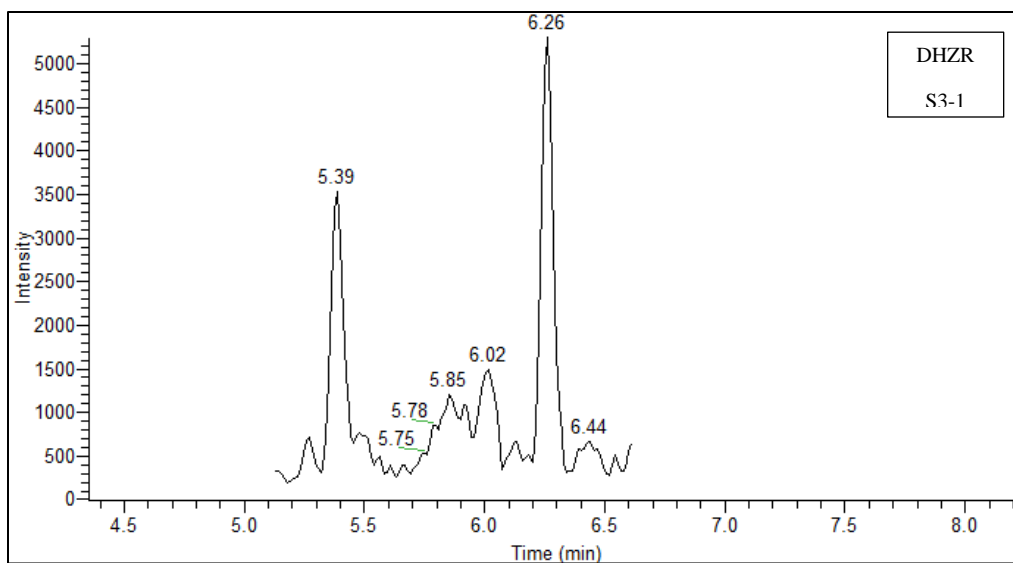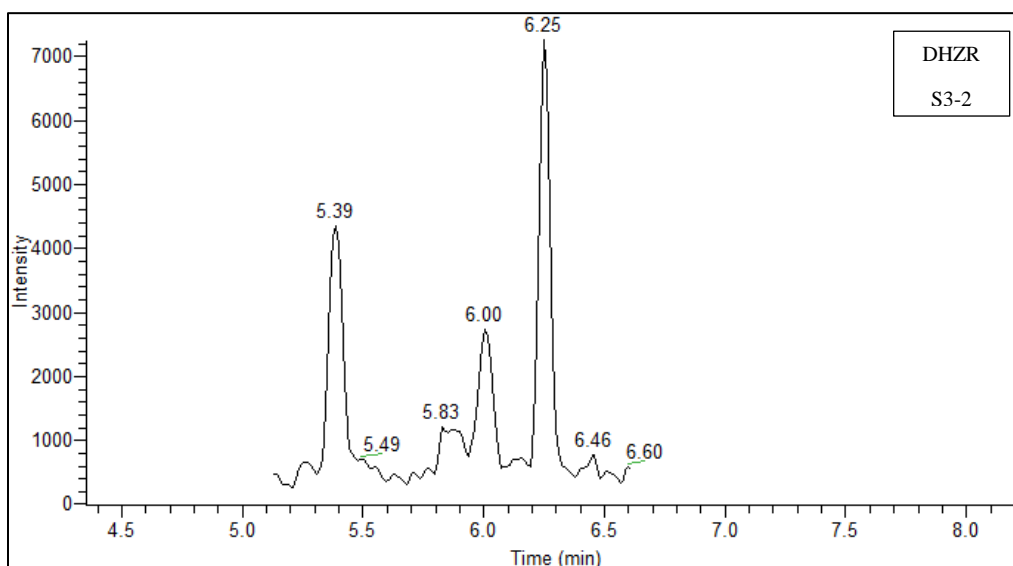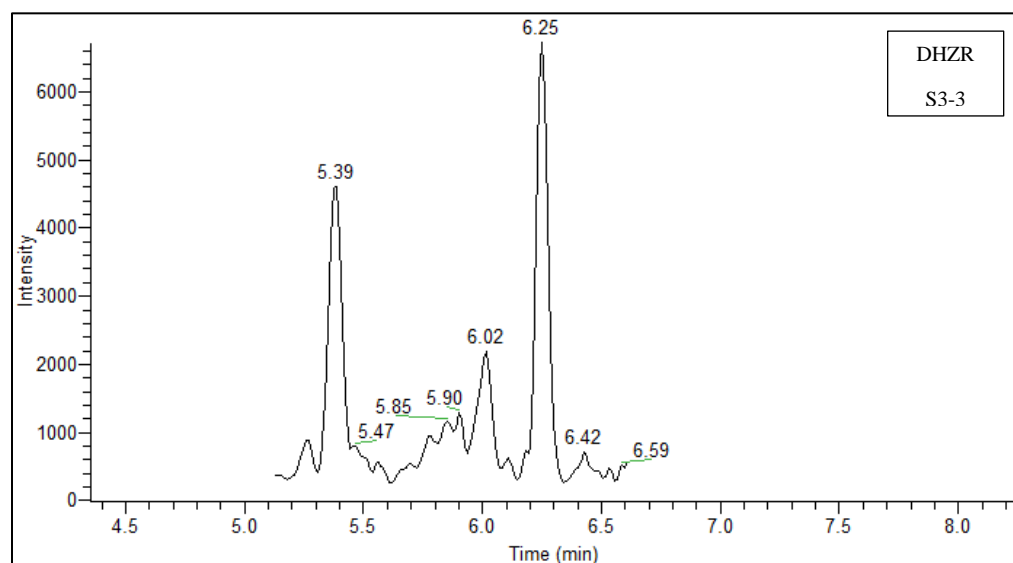

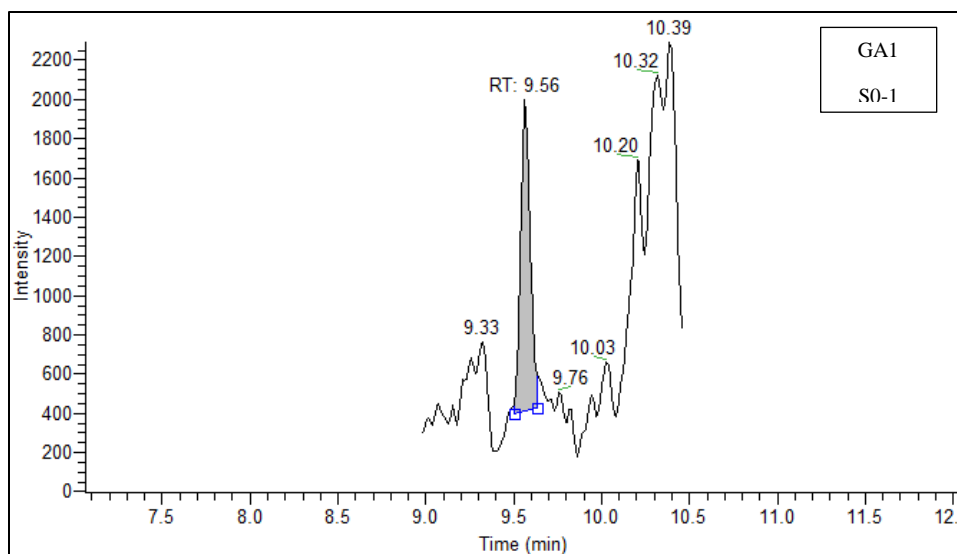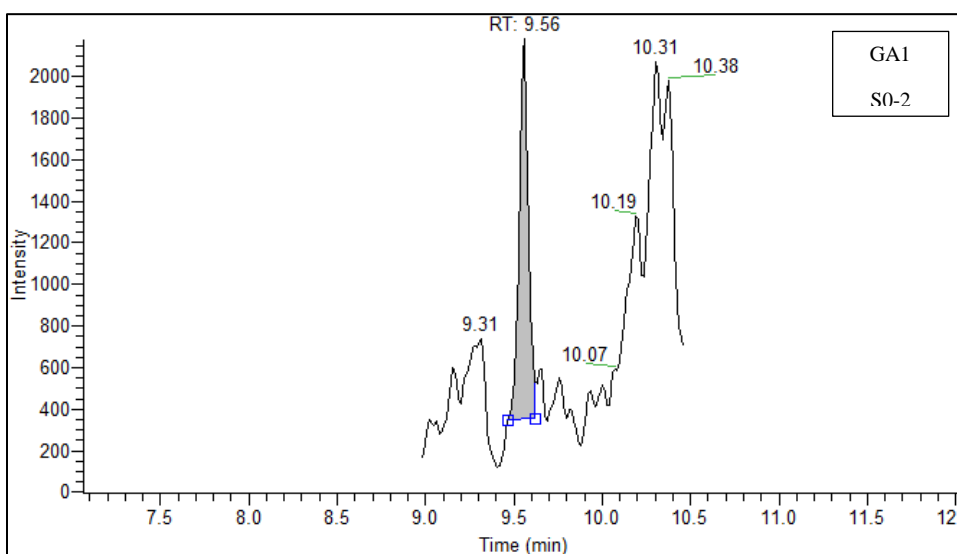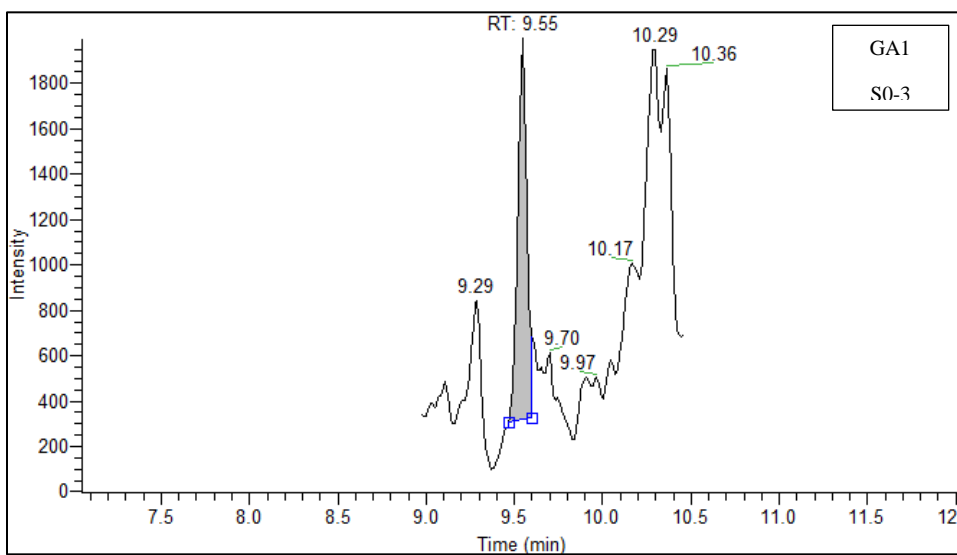

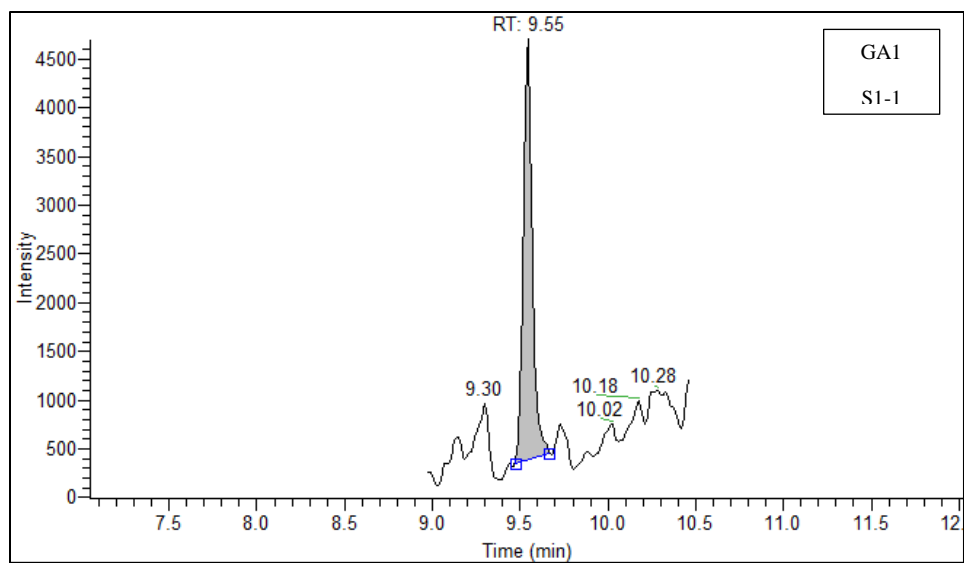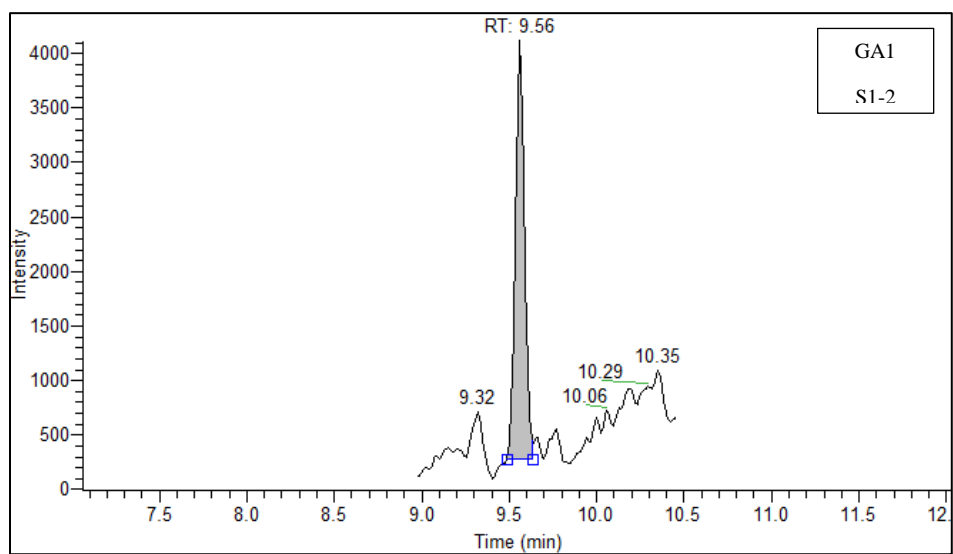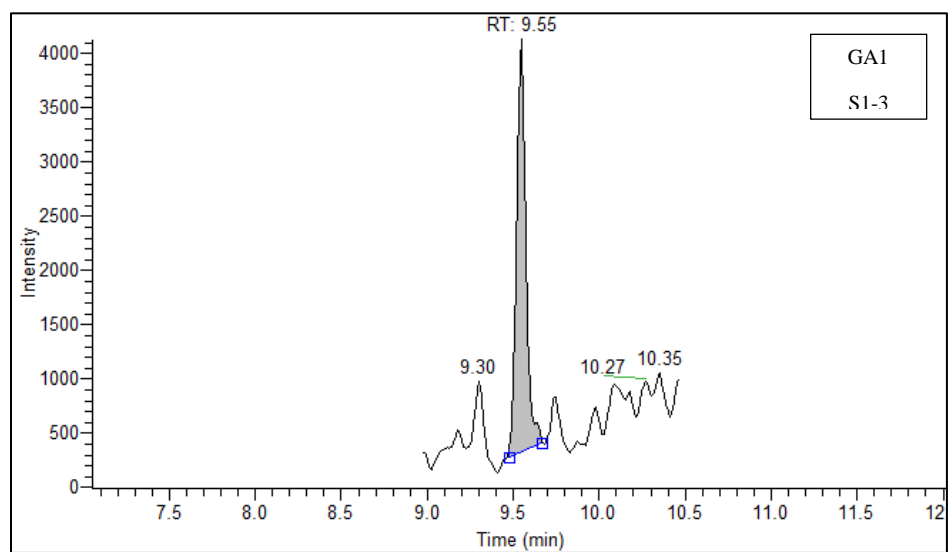

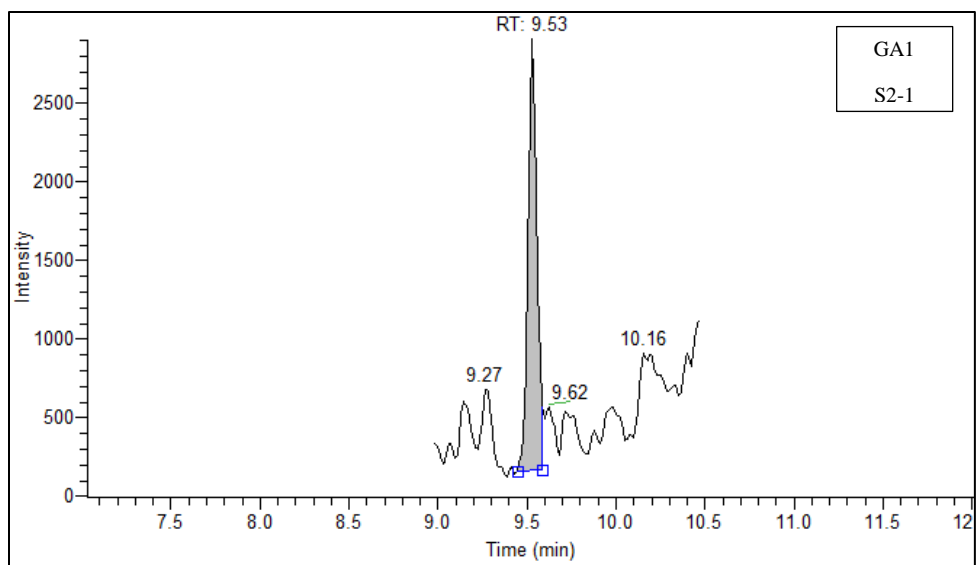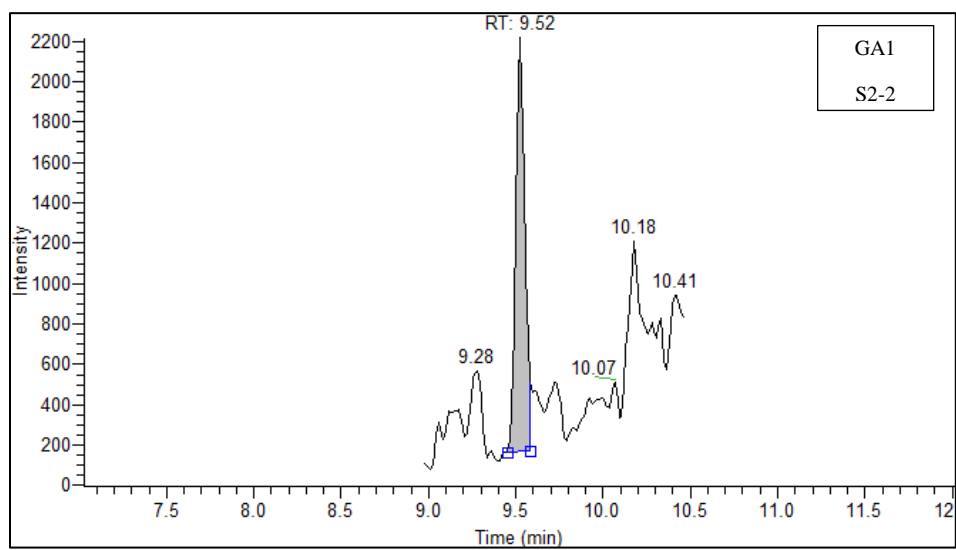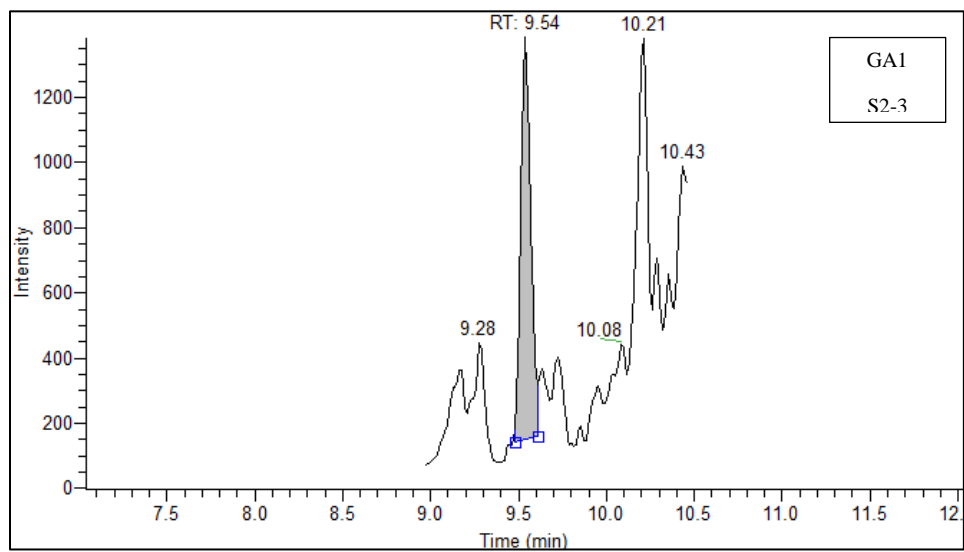

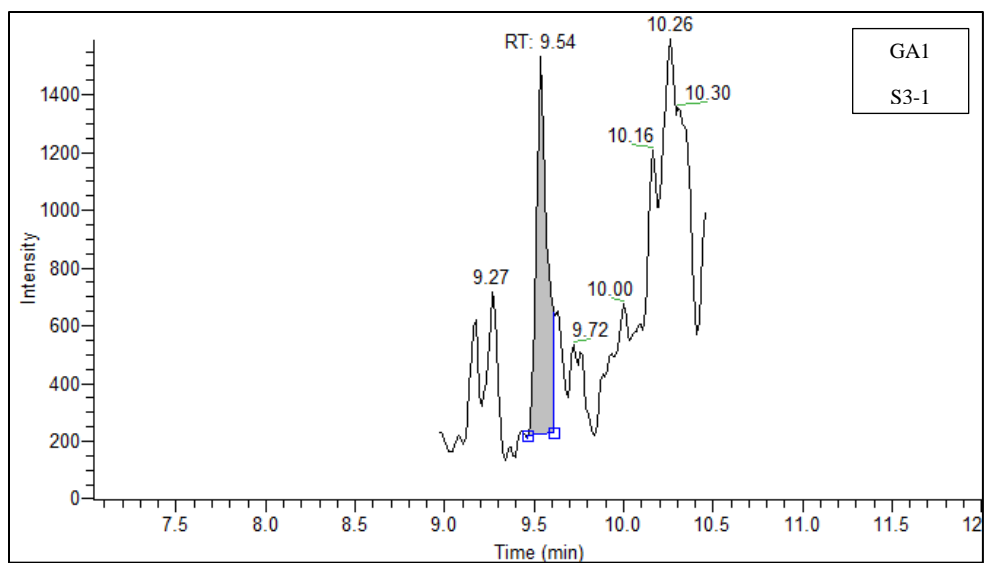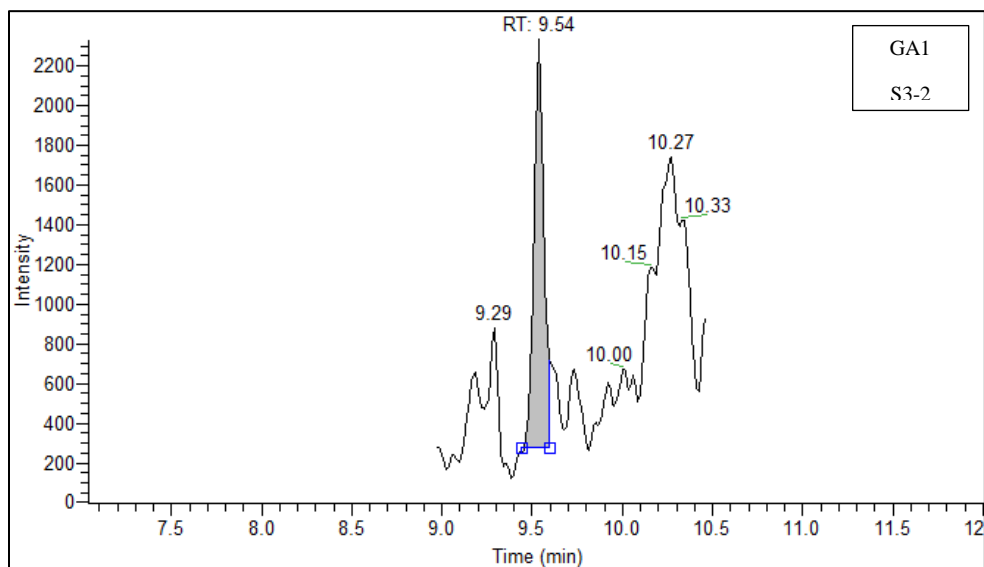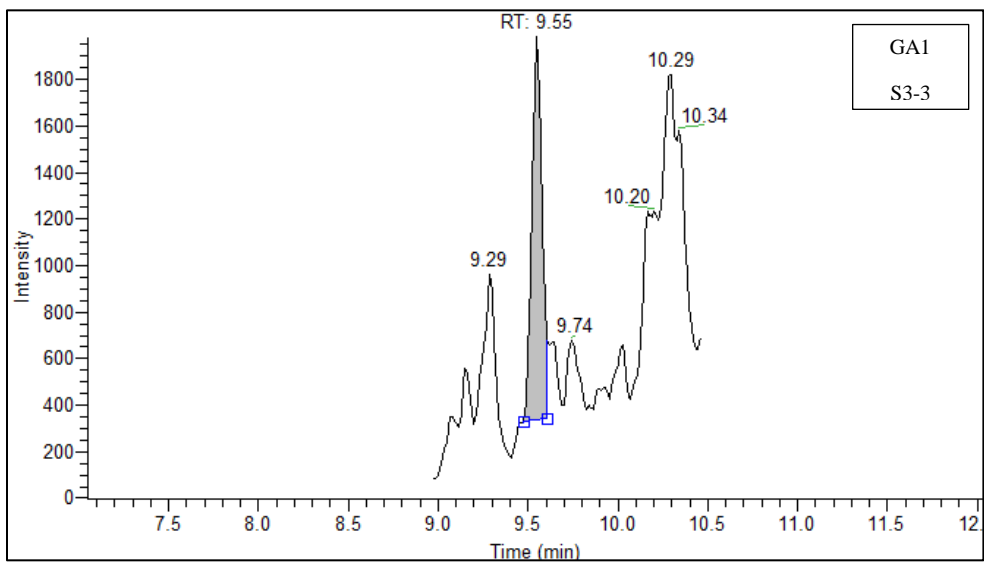

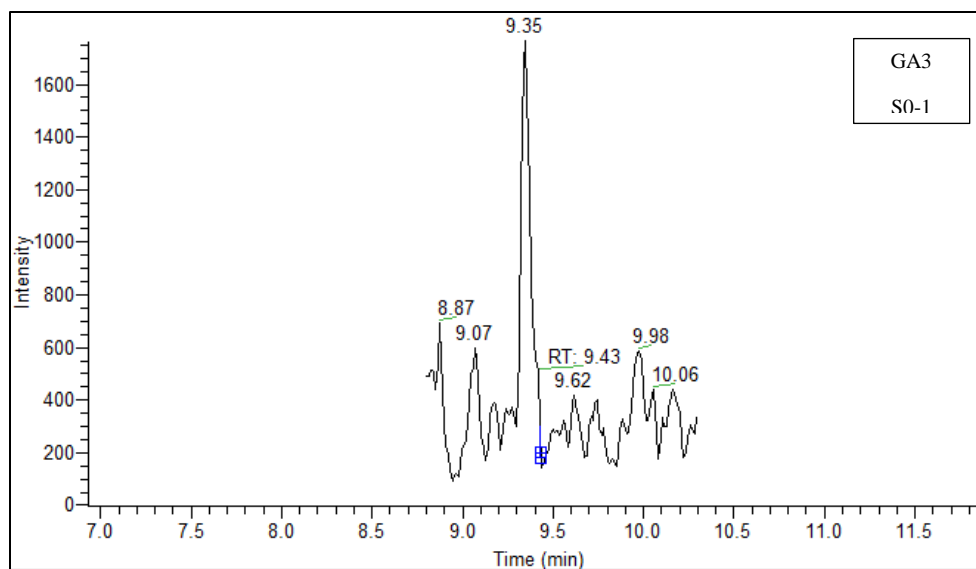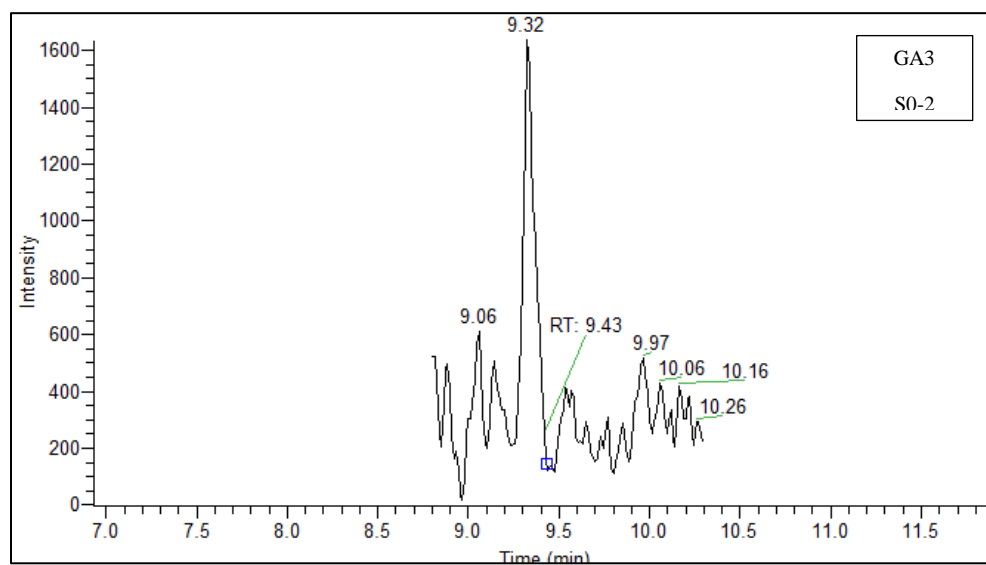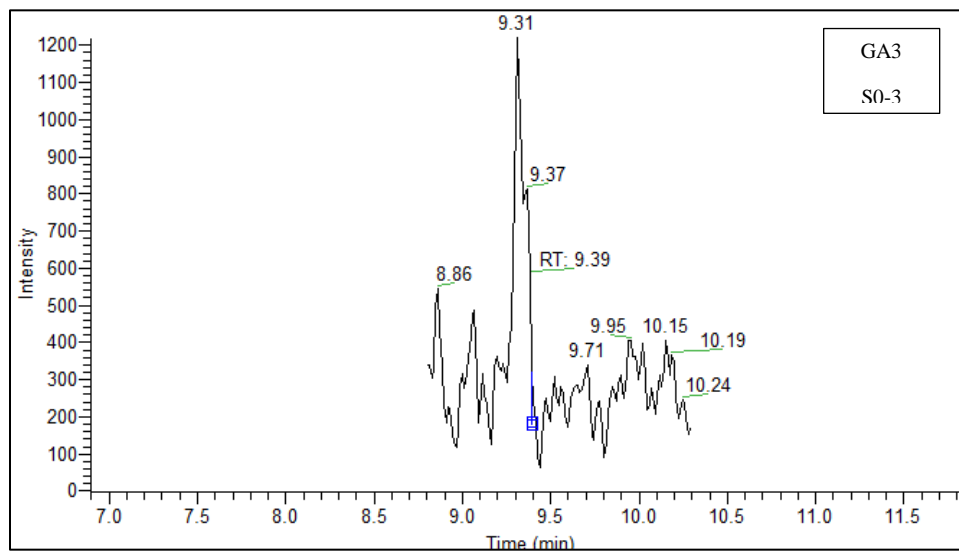

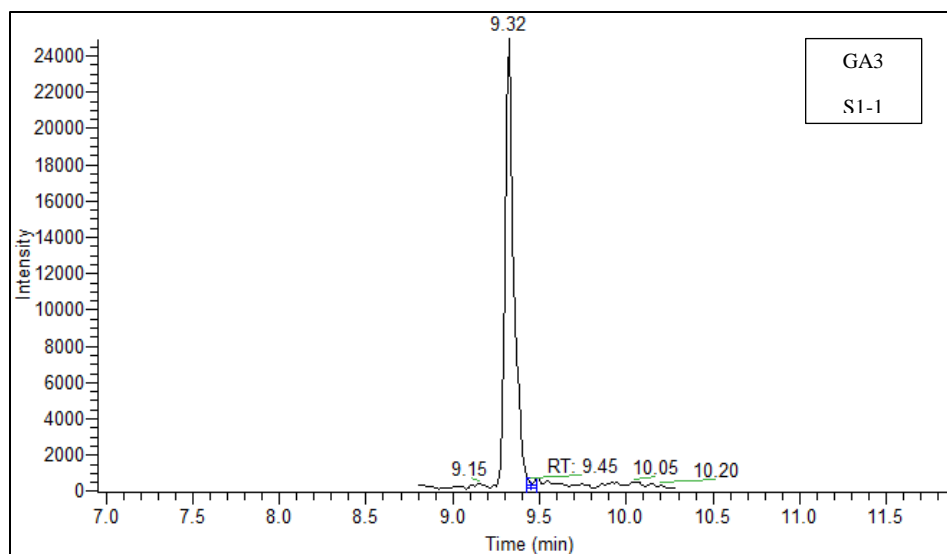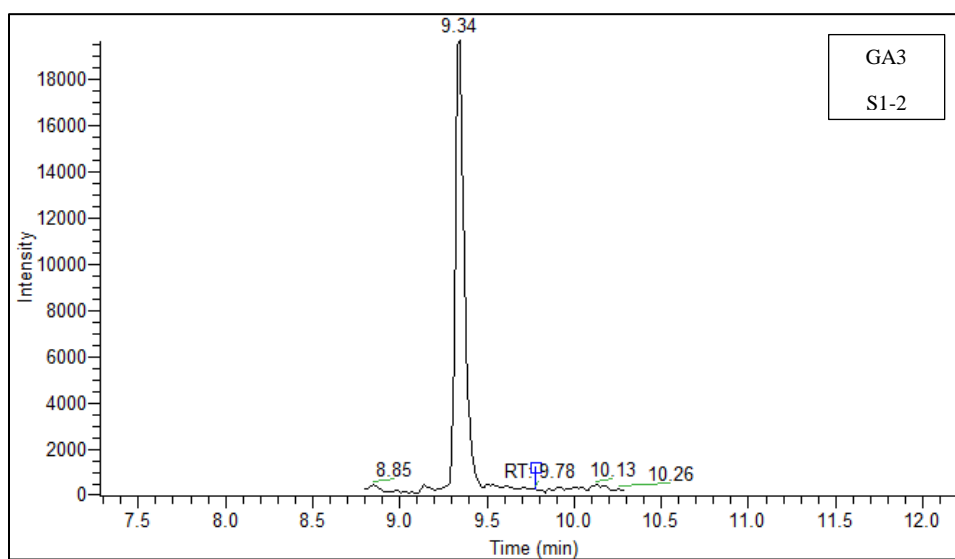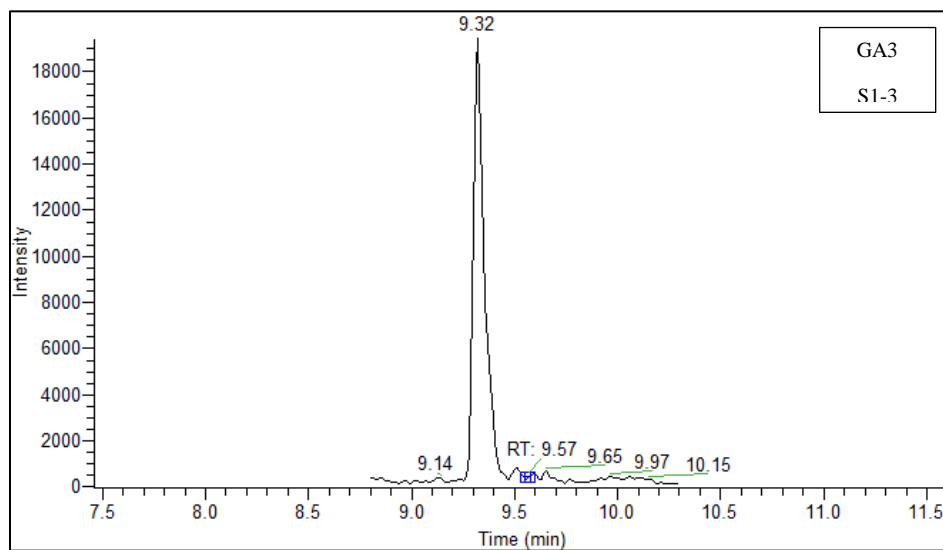

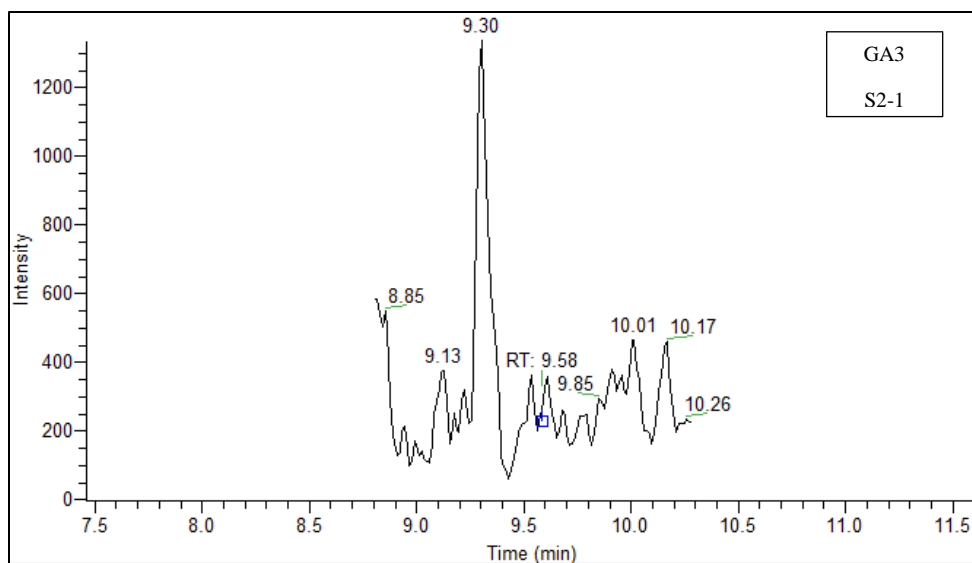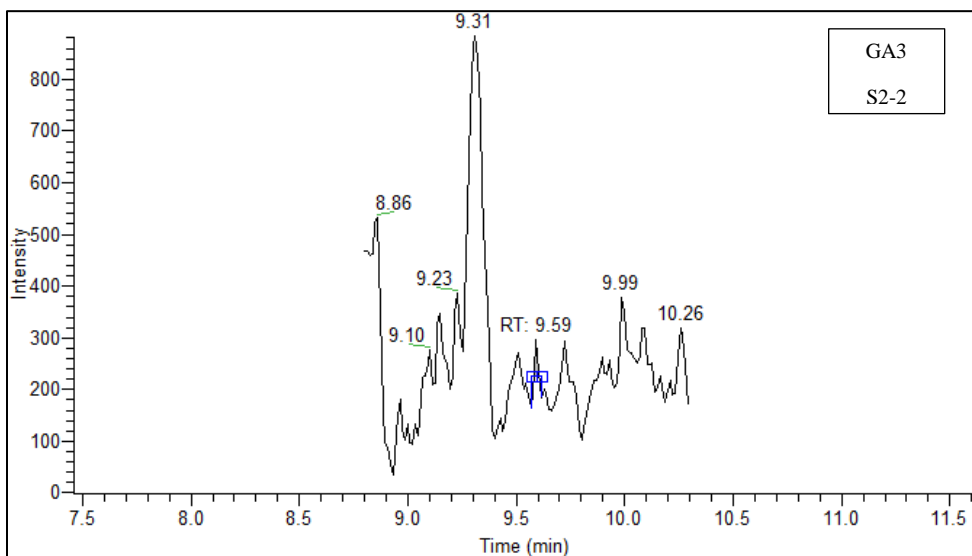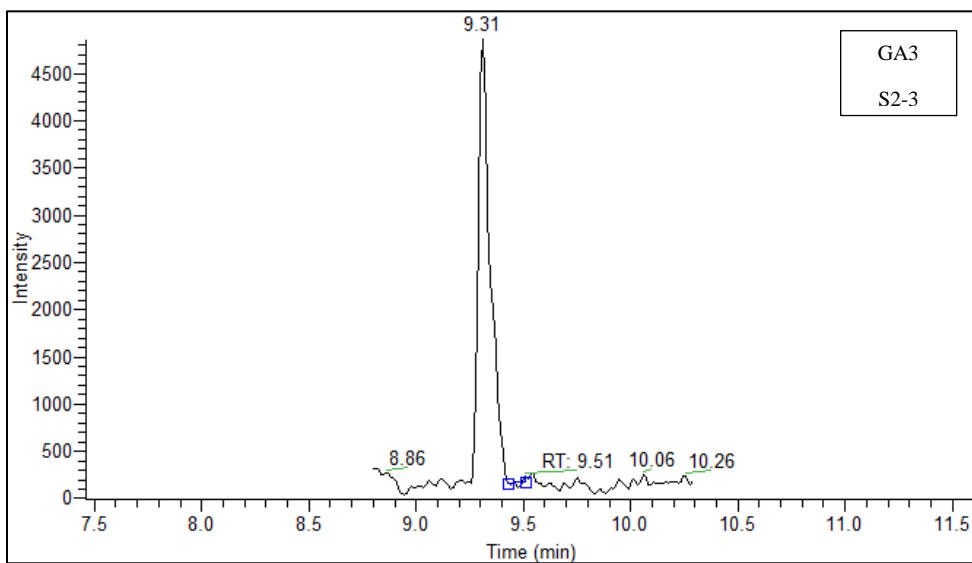

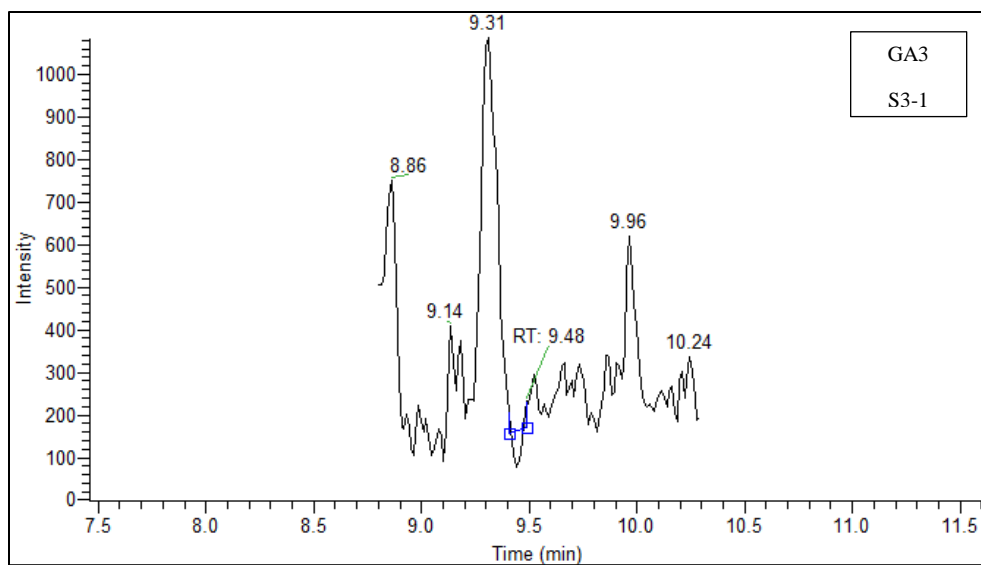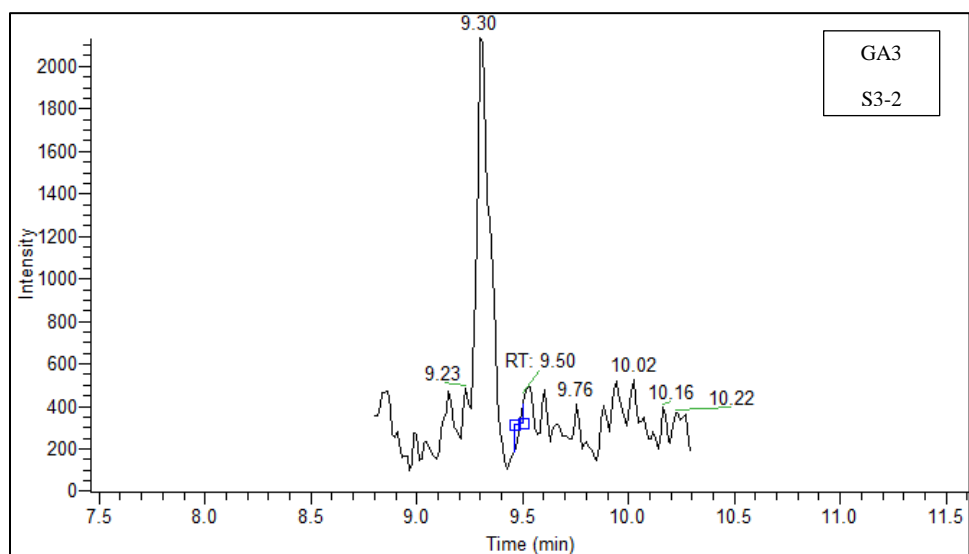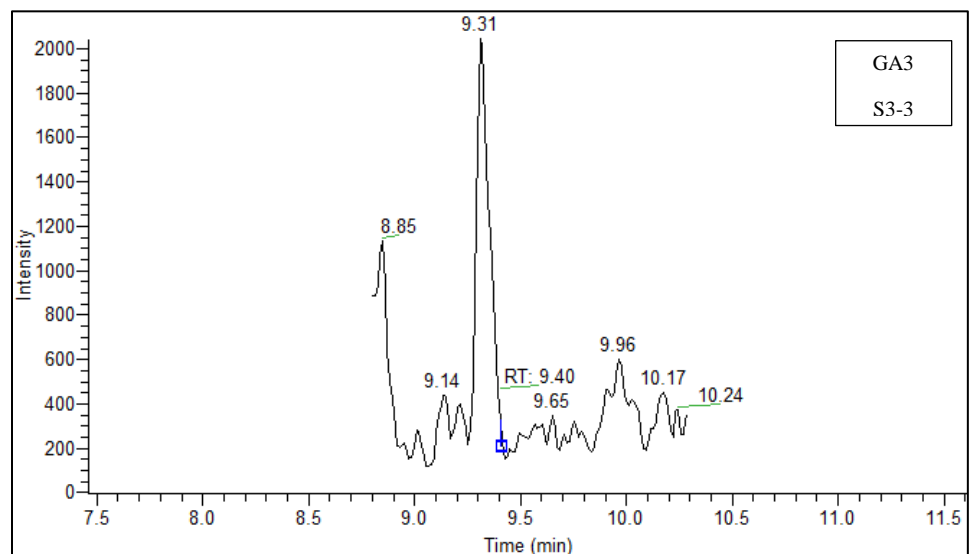

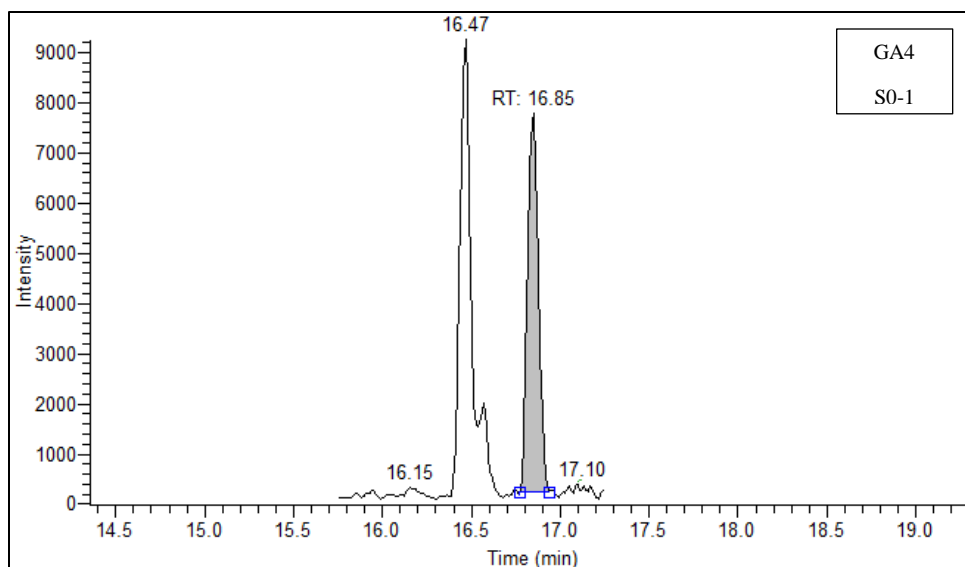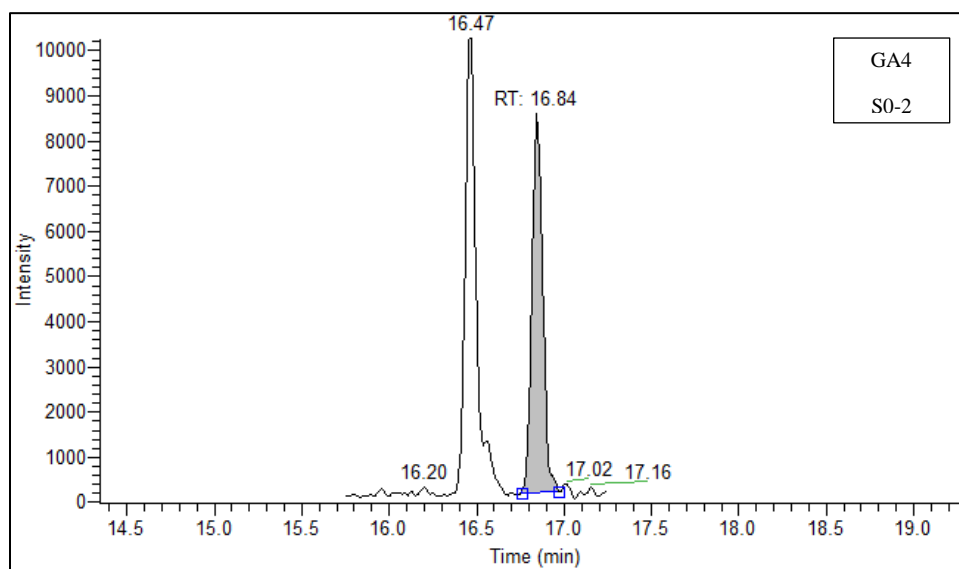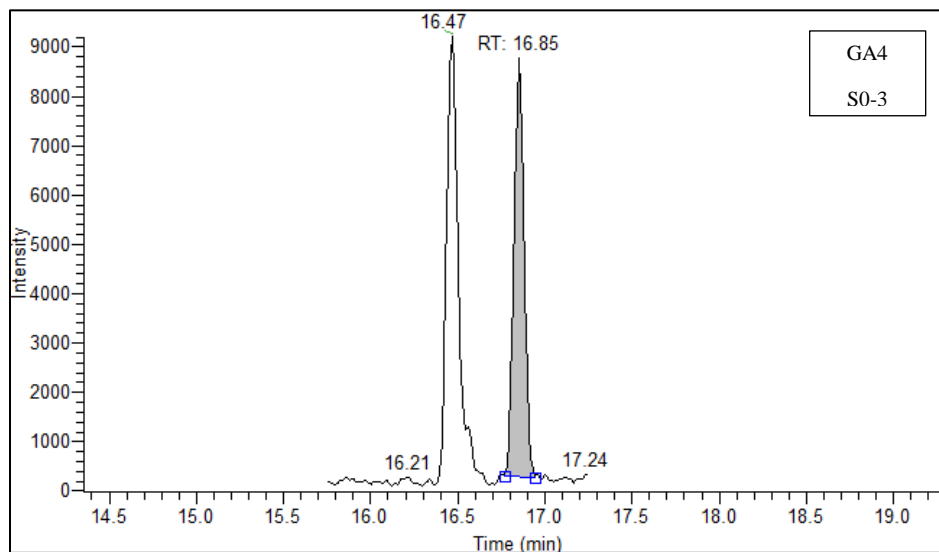

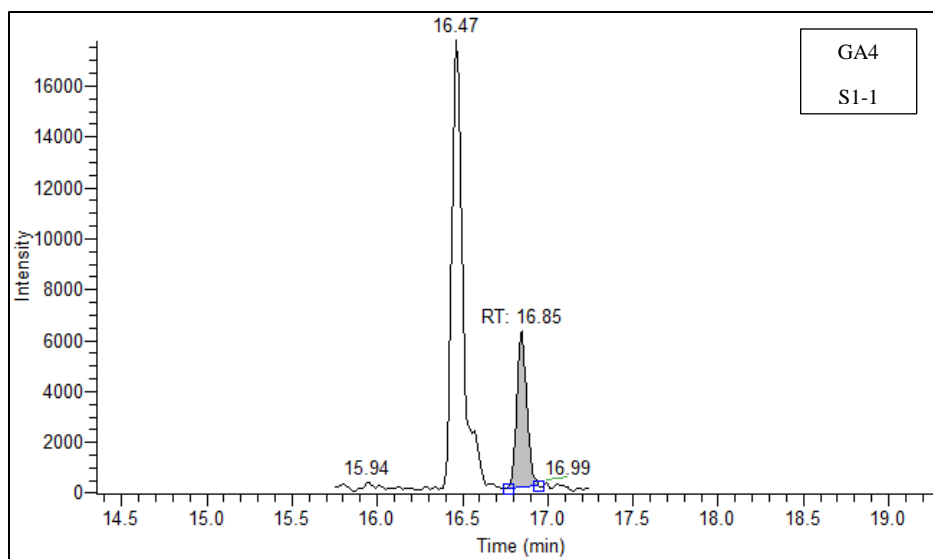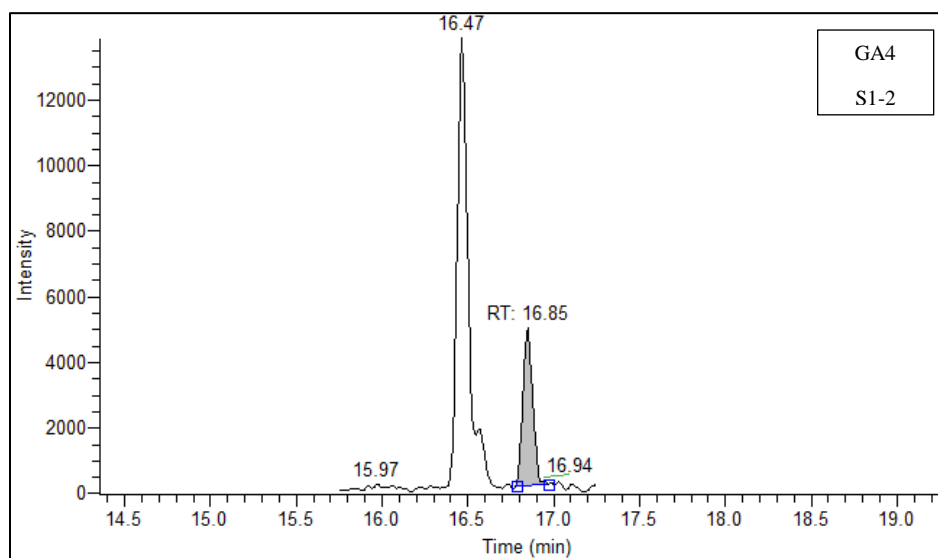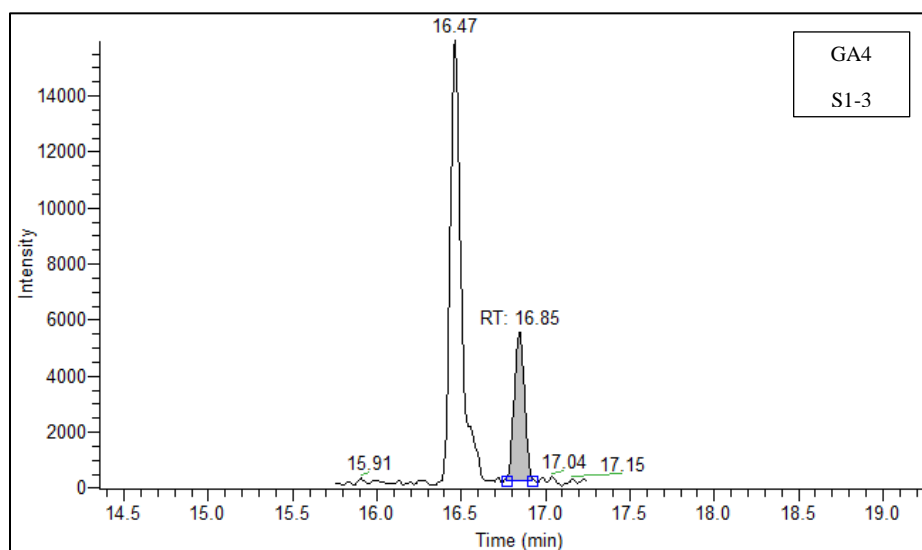

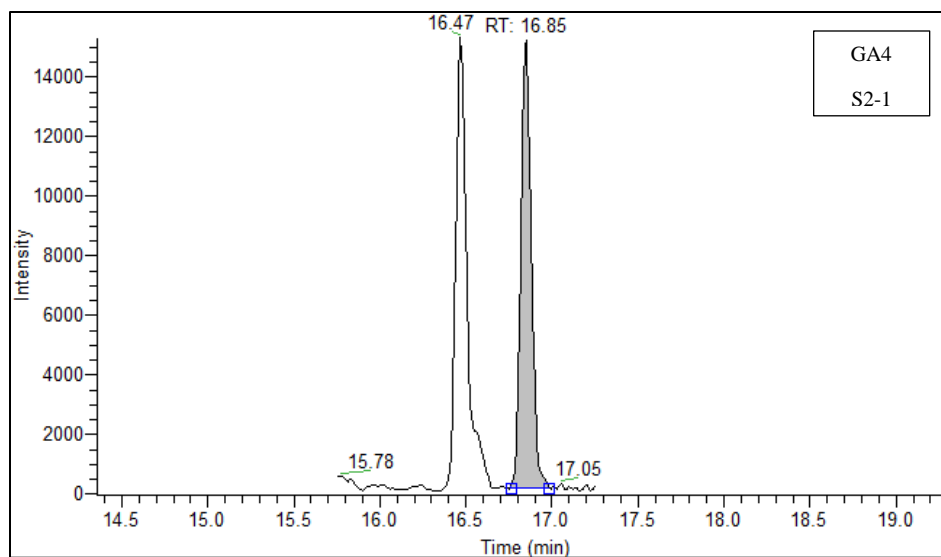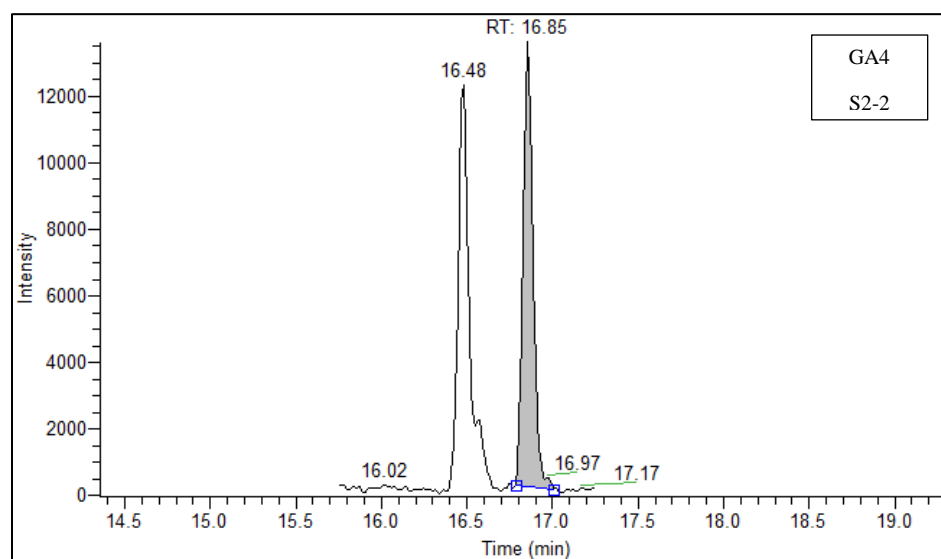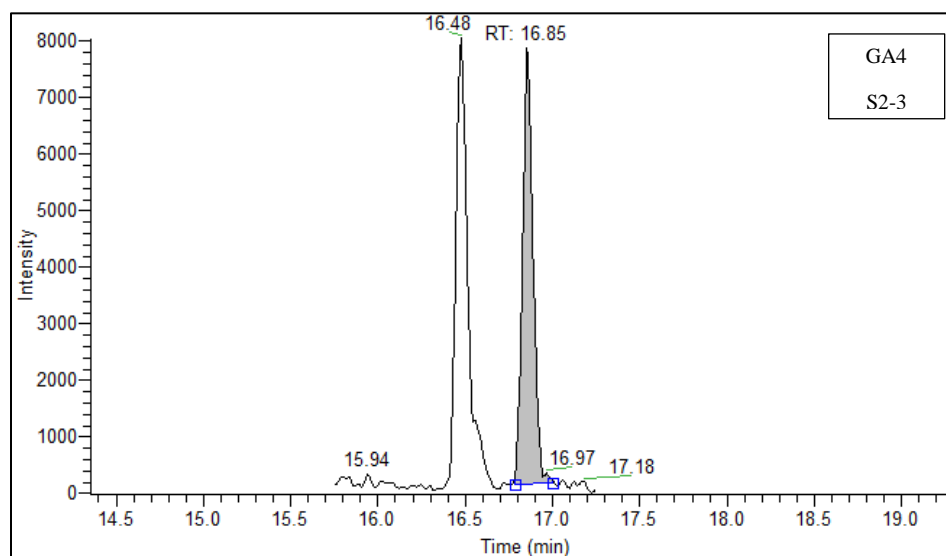

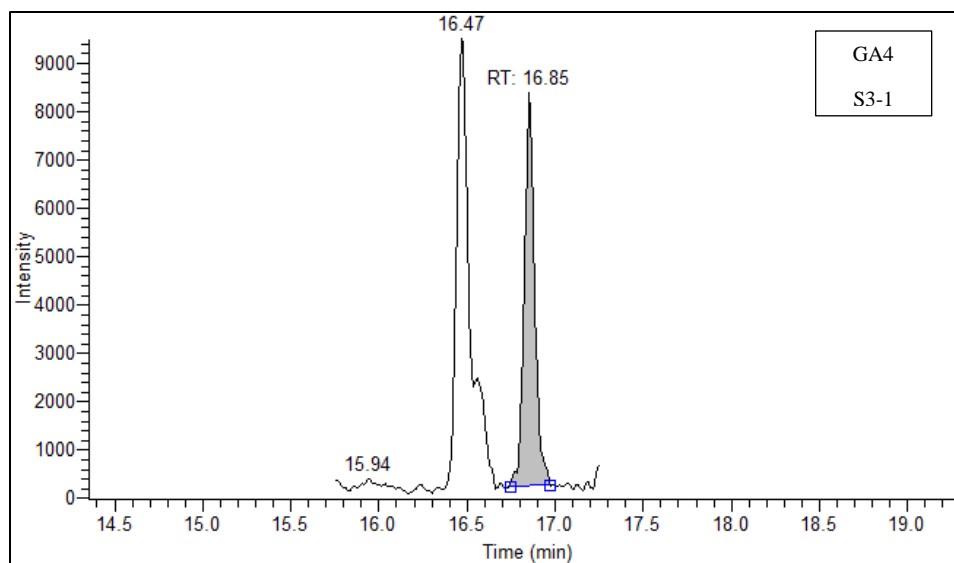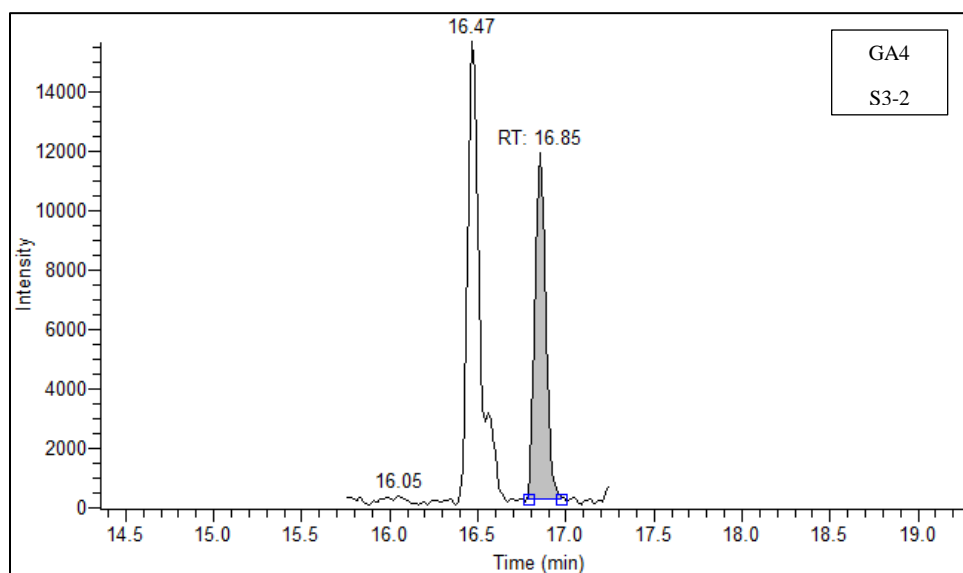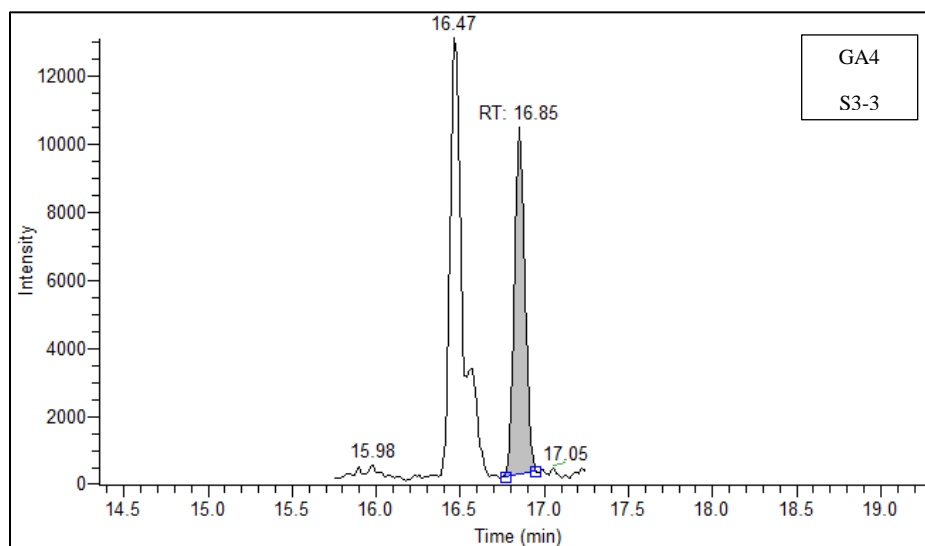

Supplement: Supplementary file 1 [file ijms-23-10461-s001.zip › Figure S1.pdf]
